# Understanding development of Mainstream US English lexical stress using semi-naturalistic stimuli

**DOI:** 10.1371/journal.pone.0345745

**Published:** 2026-04-29

**Authors:** Jill C. Thorson, Rachel Courter, Olivia H. Dempsey

**Affiliations:** Department of Communication Sciences and Disorders, University of New Hampshire, Durham, New Hampshire, United States of America; Macquarie University, AUSTRALIA

## Abstract

Lexical stress—the emphasis placed on syllables within words—is a key feature of spoken language and an important marker for diagnosing speech and language disorders. However, current assessments of prosody, including lexical stress, often rely on reading skills, lack of natural contexts, and have limited clinical utility. To address these challenges, we developed two novel lexical stress (NLS) tasks: (1) a receptive task where participants identified pictures based on the lexical stress of the word, and (2) an expressive task where participants produced target words during a picture description task. Both tasks used familiar multisyllabic words with either trochaic (strong-weak) or iambic (weak-strong) stress patterns. We tested the tasks with 40 neurotypical adults and 15 typically developing children aged 5–11 years who speak mainstream US English. Participants’ responses were judged perceptually, and expressive productions were also analyzed acoustically. Results showed that accuracy was higher on the NLS tasks compared to the PEPS-C, likely reflecting the use of familiar words embedded in meaningful contexts that reduce metalinguistic and task-related demands, thereby supporting greater ecological validity. Duration and intensity were the most reliable acoustic cues distinguishing stress patterns, while fundamental frequency (pitch) was less informative. These findings provide initial evidence that the NLS tasks are feasible, developmentally appropriate, and psychometrically promising for assessing lexical stress without literacy demands. By embedding items in meaningful contexts, the tasks may offer a more natural and clinically useful approach to evaluating prosody. Future work will expand testing with younger children and clinical populations.

## Introduction

Prosody is a critical component of speech and language, conveying both linguistic meaning and affective intent. Broadly, prosodic differences are observed across many developmental and acquired conditions, including Williams syndrome, Down syndrome, autism, and motor speech disorders (e.g., childhood apraxia of speech, dysarthria) [1,2]. However, current approaches to assessing prosody, particularly lexical stress, are limited by literacy requirements, the lack of pragmatic context, unnatural stimuli, and weak psychometrics, restricting their use for early identification and diagnosis. Developing assessments that are reliable, naturalistic, and clinically feasible is therefore a priority. The present study addresses these challenges by evaluating receptive and expressive prosody using semi-naturalistic stimuli in neurotypical individuals who are expected to have mastered these abilities [1,3].

Lexical stress contributes directly to speech naturalness and intelligibility by signaling relative prominence across syllables. While it interacts with higher-level prosodic features such as phrasal stress and focus, it serves a distinct role that warrants separate assessment in clinical contexts [4]. Unlike global prosodic disruptions, variations in lexical stress have been shown to be a diagnostic marker in specific motor speech disorders such as apraxia of speech (AOS), childhood apraxia of speech (CAS), and dysarthria. Importantly, prosodic patterns differ across disorders: reduced phrasal stress in hypokinetic dysarthria [5,6], excess and equal lexical stress in ataxic and spastic dysarthrias, and reduced lexical stress contrasts in CAS/AOS [7,8], making prosody a potentially valuable dimension for differential diagnosis. These disorder-specific patterns underscore the clinical value of assessing lexical stress as one dimension of prosody that can aid in differential diagnosis.

Lexical stress is a phonological construct conveyed through converging acoustic cues, primarily duration, intensity, and fundamental frequency (f0), with additional spectral differences arising from vowel quality shifts between stressed and unstressed syllables [9]. Stressed syllables are typically longer, louder, and higher in pitch than unstressed ones, and the combination of these cues enables listeners to distinguish trochaic (strong-weak, SW) from iambic (weak-strong, WS) patterns. For example, stress placement can change word meaning in English (e.g., *PROject*_*noun*_ vs. *proJECT*_*verb*_) [4,9–11]. While all cues contribute, evidence suggests that duration and intensity are more stable indicators of stress than f0, which can be variable across contexts and speakers [12,13]. These acoustic patterns provide the basis for both perceptual judgments and quantitative analysis of lexical stress.

Developmentally, sensitivity to stress patterns emerges early. Infants show biases for trochaic patterns during word segmentation and word learning [12,14–21]. By age three, children produce adult-like trochees, whereas iambic forms continue developing into the school years, reflecting their lower frequency in English and greater motor demands [12,17,22–24]. Preschoolers reliably perceive lexical stress when multiple acoustic cues converge (e.g., duration, intensity, and f0 acting together), but not when pitch alone signals the contrast, suggesting that some cues are less stable indicators of lexical stress [13].

Research on disordered populations is more limited and varies across diagnostic groups. In motor speech disorders such as dysarthria, prosodic impairments are well documented, with subtype-specific patterns in lexical and phrasal stress (e.g., reduced phrasal stress in hypokinetic dysarthria and excess or equal stress in ataxic and spastic dysarthrias) [6,25]. Children with CAS generally do not show broad perceptual impairments, though they may struggle with duration cues [26–28]. Children with developmental language disorder (DLD), by contrast, exhibit broader speech perception impairments, including lexical and phrasal stress [29]. In contrast, children with autism typically do not present with primary deficits in lexical stress perception, though subtle acoustic differences in production and overall prosodic naturalness have been observed [30,31]. These findings highlight both the importance of convergent cues in lexical stress perception and the need for assessment in naturalistic contexts, where multiple cues vary simultaneously.

Several assessments of prosody exist, but few are used in clinical practice. The Prosody Profile (PROP) [32], Perception of Prosody Assessment Tool (PPAT) [33], and Prosody-Voice Screening Profile (PVSP) each target specific populations or contexts but are limited by narrow scope, expressive-receptive imbalance, lack of standardization, or weak psychometrics [32–35]. A commercial tablet-based assessment is available, but it lacks published reliability and validity data and shows concerns with scoring flexibility, ceiling effects, and cultural specificity [36]. Additionally, the app lacks normative data, leaving clinicians to interpret performance independently. Given the challenges SLPs face in assessing prosody—exacerbated by the general absence of developmental norms—these limitations impact the app’s feasibility and clinical utility [37].

The most widely studied measure is the Profiling Elements of Prosody in Speech-Communication (PEPS-C), which assesses both prosodic form and function across multiple domains with receptive and expressive tasks [38–40]. Although the PEPS-C has more psychometric support than other tools, it still shows important limitations, including cultural and linguistic appropriateness, reliance on literacy, unnatural stimuli, task naturalness, and low performance even in neurotypical adults and children on lexical stress tasks [41–46]. These issues reduce confidence in its ability to capture expected developmental and clinical differences.

Reviews of existing assessments underscore variability in psychometric strength and lack of ecological validity, normative data, and developmental sensitivity [44,47]. Stronger tools must report descriptive samples, item analyses, reliability, validity, and sensitivity to developmental and cross-linguistic differences [48–50]. Given that prosodic patterns vary by language and dialect, flexibility for cultural and linguistic diversity is also critical but often overlooked [49,50].

Naturalistic and semi-naturalistic tasks may better reflect real-world communication by embedding items in meaningful contexts and reducing literacy demands. Such approaches are widely used in standardized speech and language assessments (e.g., Clinical Evaluation of Language Fundamentals, CELF-5; Preschool Language Scale, PLS-5; Goldman-Fristoe Test of Articulation, GFTA-3), where picture-based elicitation supports ecological validity, improves prediction of functional outcomes, and engages diverse populations [51–58]. Extending this design to prosody, tasks that embed lexical stress within naturalistic contexts may yield higher validity and reliability than highly controlled formats such as the PEPS-C, where task design has been linked to unexpectedly low accuracy scores [45,46]. Removing contextual components from an expressive task may influence a speaker’s prosodic ability, as noted in previous lexical stress work [45,46]. Similar effects have also been observed for receptive tasks. Quam et al. [59] showed that preschoolers with and without developmental language disorder performed differently on sound-to-meaning mapping depending on whether tasks provided a supportive context, highlighting the importance of ecological validity in both perception and production.

### Current study

The current study addresses these gaps by introducing two Novel Lexical Stress (NLS) tasks—one expressive and one receptive—that are developmentally appropriate, semi-naturalistic, and feasible for both children and adults. These tasks aim to overcome prior limitations by eliminating literacy requirements, embedding production in meaningful contexts, and including both expressive and receptive components to provide a fuller profile of ability.

Based on past research and the critiques of existing assessments, this study examines three guiding questions:

1. **Research Question 1 (RQ1)**. Are the NLS tasks feasible and ecologically valid for assessing lexical stress across ages?

- Hypothesis 1.1: Adults and children (as young as five years old) will successfully complete the NLS tasks. This is expected because the expressive task embeds target words in picture descriptions, and the receptive task uses picture-based contrasts, reducing literacy demands and providing developmentally appropriate, meaningful contexts.
- Hypothesis 1.2: Adults will perform at ceiling, while 5- to 11-year-old typically developing children will show high but not perfect accuracy. This prediction is based on evidence that lexical stress is largely acquired by age five, but developmental differences may still emerge when tasks are designed to be sensitive and naturalistic [12,15,16].

2. **Research Question 2 (RQ2)**. Do the NLS tasks demonstrate strong psychometric properties?

- Hypothesis 2.1: Item analyses will show consistent accuracy across stimuli for adults, but greater variability for children, reflecting differences in word difficulty (two vs. four syllable words). This prediction is based on evidence that neurotypical adults have fully acquired lexical stress patterns, whereas children’s performance is more sensitive to stimulus complexity and linguistic demands.
- Hypothesis 2.2: The NLS tasks will demonstrate developmental sensitivity and evidence of construct validity. This is particularly anticipated in the receptive tasks because the tasks capture converging acoustic cues in naturalistic contexts, which better align with known developmental patterns showing that by five years old children have intact processing of lexical stress when all acoustic cues are present [13].

3. **Research Question 3 (RQ3)**. How do performance patterns of neurotypical adults and children on the newly developed NLS tasks compare to those on the PEPS-C lexical stress tasks?

- Hypothesis 3.1: Neurotypical adults and typically developing children will exhibit higher accuracy on the NLS tasks than on the PEPS-C tasks, reflecting the advantages of naturalistic settings that provide a more ecologically valid representation of ability and addressing reported challenges with the PEPS-C lexical stress tasks (e.g., literacy demands, unnatural stimuli).
- Hypothesis 3.2: Acoustic analyses will reveal that duration and intensity, but not f0, differentiate lexical stress patterns, with greater developmental change for iambic words than trochaic words. This is grounded in prior work in Australian English showing that f0 is a more variable and less reliable cue to contrast lexical stress for children aged five years [12]. Additionally, this research shows that acoustic-phonetic realizations for trochees are adult-like by age three but iambs continue to develop until at least age seven as they require later developing motoric and phonological skills.

## Method

### Participants

There were two groups of participants: neurotypical adults and 5- to 11-year-old typically developing children. Adult participants were recruited from university courses (receiving course credit) or posted flyers in the community (receiving a monetary incentive). There were 41 adult participants (self-identifying as 9 males and 32 females) ranging from 18 to 66 years old (*M* = 22, *SD* = 8.98). Partial data from two adults were excluded due to technical malfunctions (one full exclusion and one excluded from the PEPS-C analysis only). The self-identified racial and ethnic breakdown for the included adults was: White (39) and Hispanic or Latino (1). All participants spoke US English as one of their first languages, with three individuals also speaking another language (Spanish (2); Russian (1)). All adult participants were raised in the northeast region of the USA: NH (20), MA (9), ME (3), CT (3), RI (2), NJ (2), and NY (1).

Fifteen child participants were recruited from the community (7 males and 8 females) and received a monetary incentive and a book for completion. Children ranged from 5 to 11 years old (*M* = 7.80, *SD* = 2.11). No child data were excluded. Four children were not able to read and thus were unable to complete the expressive PEPS-C tasks, which require literacy skills (three five-year-olds and one six-year-old). For analyses, children were grouped either altogether as one child group or split by broad age groups: older children (8- to 11-year-olds; n = 8) and younger children (5- to 7-year-olds; n = 7). When the data from the children who could not read were removed from the analyses, only three participants in the younger group remained for the expressive tasks, making this type of split very unbalanced for analyses.

The self-identified racial and ethnic breakdown for the children was: White (n = 14) and Black or African American and White (1). All child participants were being raised in either New Hampshire (13) or Maine (2). All adult and child participants met the inclusion criteria of no reported speech-language disorder, native speaker of US English, no other developmental, neurological, or genetic disorder, normal hearing, and normal or corrected-to-normal vision.

### Novel lexical stress (NLS) tasks

Two novel lexical stress (NLS) tasks were designed: the NLS receptive task and NLS expressive task. A child-friendly task was designed for the receptive NLS task, and a semi-naturalistic approach was employed to collect a spontaneous speech sample for the expressive NLS task. Both tasks were administered via PowerPoint on a computer screen.

**Fig 1:**
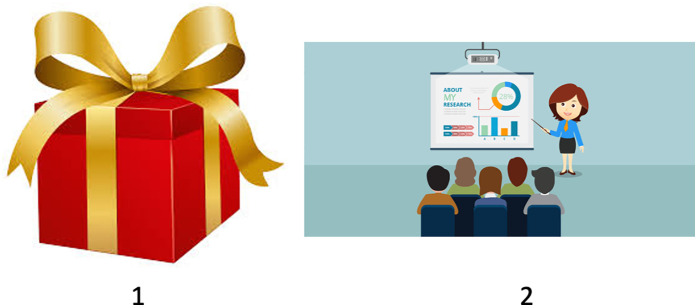
Example noun/verb picture pair for the NLS Receptive Task. PREsent (left) and preSENT (right).

In the receptive task, participants first completed a vocabulary review of 12 picture-word pairs to confirm familiarity with the pictured items and align intended labels with the target stimuli. During the task, they heard an auditory stimulus of a disyllabic word and chose between two images on a computer screen that differed by lexical stress pattern (Fig 1). Instructions were: “*You will hear a word, I want you to pick if you heard the word matches image number one [points], or image number two [points]. You can tell me or point to the screen to choose your answer.*” The experimenter recorded responses.

**Fig 2:**
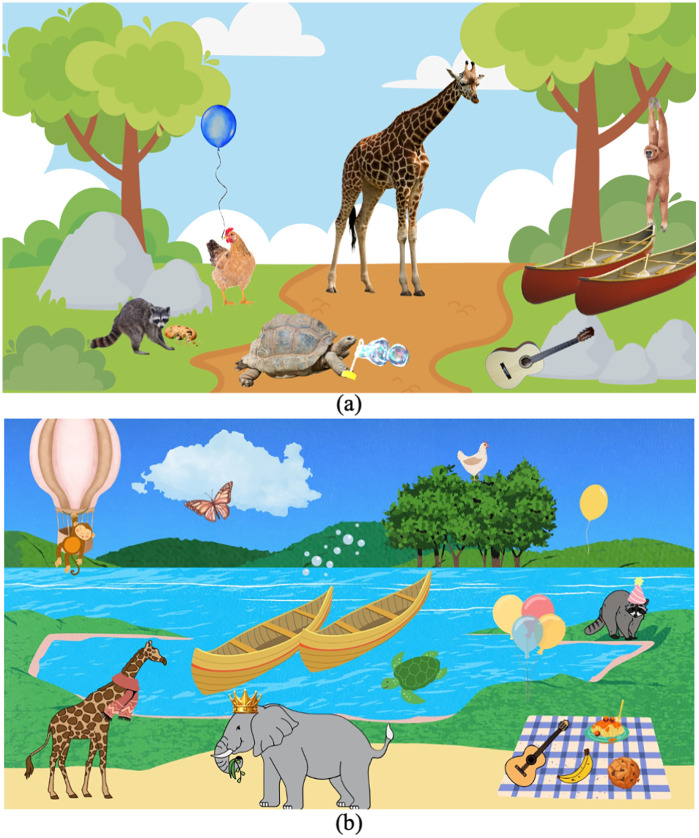
Visual scenes for the NLS expressive task. (A) adult version with 10 targets; (B) child version with 14 targets.

In the expressive task, a vocabulary check was performed first where participants reviewed 10 (adult version) or 14 (child version) target pictures to confirm item labels. Unlike the receptive review, labels were not modeled aloud to avoid influencing participant production (e.g., the examiner asked *what is this?* alongside a picture of the target item. If a child said a different label, they were asked if there was another name for the item. All images were identifiable for all participants). Participants then described a complex scene containing all target items (Fig 2). Adult instructions were: “Please describe everything that you see in this picture. Tell me as many details as possible. What are the animals doing?” whereas the child instructions were: “Tell me a story about this picture. Use as many details as possible.” After piloting, the child instructions were revised to encourage full sentences. If target words were omitted, the experimenter prompted: “Tell me more about what’s happening in this part of the picture,” without providing feedback or modeling. Target words were coded for utterance position (initial, medial, or final) and lexical stress (first syllable, second syllable, or ambiguous). Both the receptive and expressive tasks were not scored automatically to provide flexibility to the examiner (e.g., having a different lexical stress target due to potential dialectal variations).

Expressive productions were assessed both perceptually and acoustically. Perceptual ratings remain the clinical gold standard (e.g., for motor speech disorders) [60], while acoustic analyses can reveal contrasts not always perceived by listeners (e.g., covert contrasts) [25,61,62]. Measures such as the Pairwise Variability Index (PVI) for duration, f0, and intensity provide complementary detail [25,63,64]. Using both methods captures nuances that might be missed when relying on either approach alone, leading to a more complete picture of ability [45,65]. Details of the acoustic analysis are presented in Acoustic Analysis and Reliability section.

#### Stimuli.

Receptive stimuli were disyllabic words in mainstream US English that shift stress by syntactic class (e.g., noun-verb pairs such as *PROject* vs. *proJECT*). This set is limited in size but offers phonological similarity with controlled stress variation (with some vowel differences due to reduced stress). Only visually imageable nouns/verbs were selected, eliminating items difficult to depict (e.g., *INsight/inCITE*). Images of the stimuli, rather than orthography, were used to prompt responses to avoid literacy demands. Because many of these items are acquired later, full age-appropriateness could not be achieved. However, the average Age of Acquisition (AoA) was 7.97 years old (*SD =* 2.34; *range* = 4.83–12 years old) [66]. Only one of the NLS receptive items (*PREsent*_*noun*_) appears on the MacArthur-Bates CDI (MCDI) Words and Sentences for 16–30 months ([67]), accounting for 8.33% of the stimuli. The Words and Sentences (Form B) was used as a reference because it assesses a broader expressive vocabulary than the Words and Gestures form (Form A), while still including early-acquired items relevant to younger children.

A vocabulary review familiarized participants with the items but did not exclude stimuli. This design choice was made to assess sensitivity to lexical stress patterns independent of full lexical mastery. Brief vocabulary reviews are sufficient for children to align labels with referents and to fast-map unfamiliar items, particularly when supported by visual context [68–70]. Excluding items based on prior lexical knowledge would reduce the ecological validity of the task, as children routinely encounter and interpret prosodic cues in partially known or novel words in everyday communication. Moreover, English-learning children show sensitivity to stress regularities (e.g., the predominance of trochaic patterns), which supports their ability to interpret stress contrast even when lexical knowledge is incomplete [18]. Table 1 lists the receptive stimuli. Since the Kuperman et al. (2012) AoA norms are reported at the word form level (rather than by syntactic class), homographic noun-verb pairs are assigned a single AoA value; this reflects the most salient or frequent interpretation available to raters.

**Table 1:** NLS receptive stimuli.

Item	IPA	CV	Syllable Stress	Syntactic Class	AoA Kuperman *M (SD)*
CONverse	/ˈkɔnvɚs/	CVCCVC	1	N	12.00 (2.90)
conVERSE	/kənˈvɜ˞s/	CVCCVC	2	V	12.00 (2.90)
PROject	/ˈprɑdʒɛkt/	CVCVCC	1	N	9.00 (3.97)
proJECT	/prəˈdʒɛkt/	CVCVCC	2	V	9.00 (3.97)
PROduce	/ˈprodus/	CCVCVC	1	N	7.75 (3.11)
proDUCE	/prəˈdus/	CCVCVC	2	V	7.75 (3.11)
PREsent	/ˈprɛzɪnt/	CCVCVCC	1	N	4.83 (2.01)
preSENT	/prəˈzɛnt/	CCVCVCC	2	V	4.83 (2.01)
REcord	/ˈrɛkɚd/	CVCVC	1	N	6.30 (2.60)
reCORD	/rəˈkɔrd/	CVCVC	2	V	6.30 (2.60)
OBject	/ˈɑbdʒɛkt/	VCCVCC	1	N	7.94 (3.04)
obJECT	/əbˈdʒɛkt/	VCCVCC	2	V	7.94 (3.04)
				***Mean (SD*)**	**7.97 (0.97)**

Notes: English orthography, IPA transcription, CV sequence, syllable stress location, syntactic class, and mean age of acquisition (AoA) following Kuperman (and SD) for each item.

Expressive stimuli consisted of picture scenes containing images of 10 two-syllable nouns (five trochaic, five iambic) in the adult version and 14 in the child version (with four additional three-syllable nouns). Two-syllable items followed common phonological patterns (trochees: CVCV(C); iambs: CVCVC) to reduce variability. All items were visually depictable, age-appropriate, and largely familiar to children: 79% appear on the MCDI Words and Sentences for 16–30 months Form B ([67]). Expressive items had an average AoA of 4.42 years old (*SD =* 0.97; *range* = 3.26–6.79 years old) [66], and all available imageability and concreteness ratings exceeded 550 on a scale of 100–700 (with higher numbers as more imageable and concrete) [71]. Table 2 lists the expressive stimuli.

**Table 2:** NLS expressive stimuli.

Item	IPA*	CV	Syllable Stress	# of Syllables	% Sonorant	AoA Kuperman *M (SD)*	MCDI Form B
cookie	/ˈkʊki/	CVCV	1	2	50.00	3.37 (1.86)	Y
chicken	/ˈtʃɪkən/	CVCVC	1	2	60.00	3.26 (1.73)	Y
turtle	/ˈtɝɾəl/	CVCVC	1	2	60.00	4.17 (1.58)	Y
monkey	/ˈmʌŋki/	CVCCV	1	2	80.00	4.21 (1.40)	Y
bubbles	/ˈbʌbəlz/	CVCVCC	1	2	50.00	3.79 (1.78)	Y
balloon	/bəˈlun/	CVCVC	2	2	80.00	4.37 (1.95)	Y
canoes	/kəˈnuz/	CVCVC	2	2	60.00	6.63 (1.54)	N
giraffe	/dʒəˈræf/	CVCVC	2	2	50.00	5.00 (3.25)	Y
guitar	/gɪˈtɑr/	CVCVC	2	2	60.00	5.32 (1.20)	N
raccoon	/ræˈkun/	CVCVC	2	2	80.00	6.79 (4.79)	N
*Only in child picture:*							
butterfly	/ˈbʌɾɚflɑɪ/	CVCVCCV	1	3	57.14	3.67 (2.14)	Y
elephant	/ˈɛləfɪnt/	VCVCVCC	1	3	71.43	4.80 (1.74)	Y
banana	/bəˈnænə/	CVCVCV	2	3	83.33	3.78 (1.17)	Y
spaghetti	/spəˈgɛɾi/	CCVCVCV	2	3	42.86	4.33 (1.19)	Y
			***Mean (SD*)**	**2.29 (0.50)**	**63.20 (0.13)**	**4.54 (1.09)**	**78.57%**

English orthography, IPA transcription, CV sequence, syllable stress location, number of syllables, percent sonorant (# of sonorant sounds/# of total sounds), mean age of acquisition (AoA) following Kuperman (and SD), and inclusion on MCDI Form B Words and Sentences (Y = yes on form; N = not on form) for each item.

*Note: This is the IPA transcription for mainstream US English. Transcriptions and syllable stress location may vary by dialect.

Stimuli were designed for mainstream US English speakers, though dialectal variation is expected. Administrators should adjust target stress patterns where dialectal differences occur (e.g., African American English and some Southern US dialects might shift some or all the target stress patterns to trochaic that are marked as iambic in the current version). This flexibility allows adaptation to diverse cultural and linguistic backgrounds [49,50]. Further details, including sound files and comparisons with the PEPS-C lexical stress stimuli, are provided in the Appendix.

#### Procedure.

The study was conducted in accordance with the Declaration of Helsinki and obtained ethics approval from the University of New Hampshire Institutional Review Board for IRB-FY2023–5 (10/15/2022 to 04/15/2023) and IRB-FY2024–2 (11/07/2023 to 09/05/2024). Adult participants and legal guardians of child participants provided written informed consent. Children gave verbal assent using age-appropriate language, witnessed by a second researcher and legal guardian, and documented in a secure Qualtrics form as ‘obtained’ or ‘not obtained.’

Demographic forms were completed by adult participants or the caregivers of the child participants. All participants passed a pure tone hearing screening (500, 1000, 2000, 4000 Hz at 25 dB HL in at least one ear) and a vision screening (adults: reading a sentence at arm’s length distance; children: describing a picture at arm’s length). Children also completed the Clinical Evaluation of Language Fundamentals, Fifth Edition (CELF-5) screener and were required to score within 1 SD of the age-appropriate criterion [55]. Auditory stimuli were presented through two Creative Inspire T12 speakers placed equal distance from the participant. Participants were seated ~18 inches from the computer and speakers, with volume held constant. Responses were audio recorded using a Shure WH20 headset microphone and Zoom H4n Pro Handy Recorder (44.1 kHz sampling rate).

The receptive and expressive lexical stress subtests of the PEPS-C were administered first, followed by the NLS tasks. The PEPS-C was administered first because participants sometimes struggled to complete it at the end of the session (see limitations). A vocabulary review presented images and auditory stimuli, and participants heard each item produced by the examiner before test items, providing a model for future productions. The PEPS-C receptive and expressive lexical subtests use the same set of lexical items, differing only in task demands. In the receptive task, participants heard a disyllabic word and chose between two orthographic representations with corresponding visual lexical stress cues (i.e., smaller and bigger circles above the syllables indicating stress such as with *IMprint/imPRINT*). Stimuli included standard lexical stress-contrast pairs (e.g., *INsight/inCITE*, *IMport/imPORT*, *INcrease/inCREASE*) and one pair that contrasted a word vs. prepositional phrase (*INtern/in TURN*). The expressive task used the same items in written form, with stressed syllables in larger font and indicated by larger circles. Participants read each written item aloud. Full administration details are available in the PEPS-C manual [40].

Tasks were administered by two second year speech-language pathology master’s students, supervised by a faculty member with expertise in prosody. Both examiners were native speakers of US English (New Hampshire), had normal hearing, undergraduate training in communication sciences and disorders, and over two years of experience analyzing prosody through coursework and clinical practica. Their limited but growing clinical experience provides a realistic baseline for future SLPs who may administer these tasks.

#### Perceptual analysis.

For expressive tasks (NLS and PEPS-C), the examiner rated productions perceptually as 1 for first syllable stress, 2 for second syllable stress, or A for ambiguous, following PEPS-C conventions for those tasks. NLS items were embedded in continuous speech, whereas the PEPS-C items were produced in isolation. For receptive tasks, responses were scored as correct (1) or incorrect (0): the PEPS-C was scored automatically by the software, and the NLS was scored based on the picture selected, using New England pronunciation targets. For analysis, ambiguous responses were collapsed with incorrect.

To analyze receptive and expressive task responses, signal-detection theory was applied by converting mean accuracy to A-prime (A’), which accounts for both hit rates and false alarms, providing a more accurate representation of sensitivity than mean accuracy alone [72,73]. This is particularly important given potential response biases and unequal trial distributions, ensuring a more robust assessment of participants’ performance. A’ is the non-parametric alternative to d-prime, making it suitable for non-normally distributed data and small datasets. A’ was calculated for each participant following Grier’s formula [74]. A’ scores range from 0.5 (chance-level performance) to 1.0 (perfect sensitivity), with values above 0.5 indicating sensitivity greater than chance.

#### Acoustic analysis and reliability.

Acoustic analyses were completed using Praat [75]. Productions were transcribed and segmented by utterance, word, and vowel in TextGrids using auditory and visual information. Trained research assistants who met in-lab reliability standards completed all coding. A Praat script extracted duration (ms), maximum f0 (Hz), and maximum vocal intensity (dB) for each target vowel (utterance and word were extracted to align vowel values with the full utterance and corresponding word for the vowel). Maximum/peak f0 and intensity were selected to ensure comparability with previous lexical stress studies [12,64]. F0 in Hertz was converted to the equivalent rectangular bandwidth (ERB-rate) scale, which more closely reflects psychoacoustic perception than raw Hz [76].

Normalized PVIs were calculated for duration, intensity, and f0 using the equation: PVI = 100 x [(d_k_ – d_k+1_)/ ((d_k_ + d_k+1_)/2)], where d is the duration/intensity/f0 of the k^th^ syllable [77]. Normalized PVI controls for individual differences in speech rate and f0 variability (e.g., sex, gender, or age). Positive PVIs indicate trochaic (SW) patterns, negative PVIs indicate iambic (WS) patterns, and larger absolute values reflect stronger stress contrastivity.

NLS expressive items were coded as sentence-medial or sentence-final, since utterance position affects acoustic measures (e.g., phrase final lengthening, boundary rises). For comparability, analyses focused on NLS sentence-final productions and PEPS-C isolated words, both occurring at prosodic boundaries. Pitch rises were observed in ~50 NLS sentence-final tokens. Re-running models without these data did not alter the results, so all data were retained.

A second rater coded a randomly selected 15% of samples to assess acoustic reliability. Agreement on vowel boundary placement was evaluated using intraclass correlation coefficients (ICC) and absolute average point-to-point differences (APD). ICC(2,1) across 224 items was 0.89 (95% CI [0.87, 0.91], *p* < 0.001), indicating high inter-rater reliability. APD for vowel duration was 15.05 ms (*SD* = 32.30 ms). F0 values were extracted within these boundaries. Perceptual scoring reliability is reported separately in the Results section alongside psychometric analyses.

#### Statistical analyses.

All analyses were conducted in R [78] using *tidyverse*, *dplyr*, *lme4*, *ggplot2*, *emmeans,* and *viridis* (for color-blind friendly palettes) [79–84]. Assumptions were checked for each model, ensuring model appropriateness. Analytic approaches varied by research question and included descriptive analyses, reliability statistics, correlation analyses, and mixed effects modeling.

For research question 1 (feasibility of the NLS tasks), no inferential statistics were conducted. For research question 2 (psychometric properties), internal consistency was examined using Kuder-Richardson Formula 20, appropriate for dichotomous data. Alpha (α) values of ≤ 0.5 indicate low internal consistency, 0.5 ≤ α ≤ 0.7 moderate, 0.7 ≤ α < 0.9 reasonable, and α ≥ 0.9 high. Analyses were conducted separately for each subtest and version (adult, child). Inter- and intra-rater reliability were assessed with Cohen’s Kappa, which accounts for chance agreement and is preferred over simple percent agreement.

For research question 3 (concurrent validity), Kendall’s tau (τ) correlations were used to examine relationships between NLS and PEPS-C accuracy (A’) by mode (expressive, receptive) and group (adults, children). Kendall’s tau was chosen due to tied ranks.

For accuracy, linear mixed effects models assessed the effects of task (NLS, PEPS-C), age group (adults, younger children, older children), and stress pattern (trochaic, iambic) on A’. Mixed effects models were specified with participant as a random intercept to account for repeated measures. Although this structure produced singularity for some models, indicating minimal variance attributable to participants, retaining the random intercept preserves the independence structure of the data. To assess model robustness, we verified that results were unchanged when the random effect was removed. Since including the participant intercept did not alter the results and maintains consistency with the repeated measures design, we report the models with the random intercept structure here. Models were fit with restricted maximum likelihood (REML), with Satterthwaite’s method for t-tests. Multicollinearity was checked using adjusted Generalized Variance Inflation Factors (GVIF^(1/(2*df))) due to the presence of categorical variables (< 5 for all models). Missing data were excluded (e.g., children unable to read PEPS-C items). Planned pairwise comparisons were Bonferroni-adjusted.

For acoustic analyses, generalized linear mixed effects models examined the relationship between PVI measures (duration, f0, intensity) and stress pattern, with random intercepts for participant. Models used a binomial logit link function and were evaluated using the Akaike Information Criterion (AIC) and the Bayesian Information Criterion (BIC). All variance inflation factors (VIF) were < 5, indicating no multicollinearity. The model had a reasonable fit with an AIC of 1092.80 and a BIC of 1117.70, with no substantial variance in the random intercept for participant (σ² = 0.00). Analyses were run on the full dataset and separately on final/isolated items; results were consistent, so the full dataset is reported (supplemental analyses provided in the S2 File).

To more closely analyze each PVI measure, three linear mixed effects models predicted duration, intensity, and f0 PVIs with fixed effects of task, group (adults, children), and stress pattern, and random intercepts for participant and item. To match across tasks, only isolated PEPS-C items and sentence-final NLS items were included. Multicollinearity was addressed via stepwise reduction when VIF > 5 [85]. All models converged with REML criteria reported in the results. Model fit indices indicated reasonable convergence. Additional models tested sentence position (medial, final) within NLS data and results are presented in the S3 File.

## Results

A total of 3022 observations were collected across both child and adult participants (n_exp_ = 1487; n_rec_ = 1539). For the adult expressive tasks, 1033 items were analyzed (396 from NLS; 637 from PEPS-C). For the adult receptive tasks, 1119 items were analyzed (479 from NLS; 640 from PEPS-C). For the child expressive tasks, 450 items were analyzed (210 from NLS; 240 from PEPS-C). For the child receptive tasks, 420 items were analyzed (180 from NLS; 240 from PEPS-C).

### RQ1: Are the NLS tasks feasible and ecologically valid for assessing lexical stress across ages?

All participants, including children as young as five years old, successfully completed both NLS receptive and expressive tasks, supporting feasibility (Hypothesis 1.1). Adults performed at ceiling across both tasks (expressive A’ = 0.995; receptive A’ = 0.990), while children showed near-ceiling performance on the expressive task (A’ = 0.989) and slightly lower accuracy on the receptive task (A’ = 0.910). These findings align with Hypothesis 1.2, suggesting that while lexical stress production is largely established by age five, receptive sensitivity continues to show developmental variability when assessed using naturalistic methods.

Contextual differences further underscore the ecological validity of the NLS tasks. In the expressive NLS tasks, most productions occurred in sentence-medial position (67% adults; 88% children), with the remainder sentence-final. By contrast, all PEPS-C expressive stimuli were produced in isolation (100%), reflecting the task’s elicited, decontextualized production format rather than sentence-level use. This integration of target words into sentence contexts in the NLS tasks more closely approximates everyday speech and highlights their ecological validity relative to the PEPS-C.

### RQ2: Do the NLS tasks demonstrate strong psychometric properties?

#### Internal consistency.

Item analyses showed low-to-moderate internal consistency for the adult NLS tasks (expressive α = 0.25; receptive α = 0.42) and somewhat higher values for the revised child tasks (expressive α = 0.599; receptive α = 0.388). The low internal consistency estimates, particularly for the adults, are likely driven by near-ceiling performance and the resulting restriction of variance, which attenuates reliability estimates for short tests with dichotomous outcomes. Consistent with Hypothesis 2.1, adults showed uniformly high accuracy across items, while children exhibited relatively greater dispersion in item-level accuracy, though this variability was limited by overall high performance. Accuracy of each NLS stimulus item is presented in the Appendix.

#### Reliability.

A randomly selected 25% of the NLS and PEPS-C expressive task data were re-rated for perceptual judgments of lexical stress by a second rater to calculate inter-rater reliability, and by the primary rater to calculate intra-rater reliability. Reliability analyses showed excellent agreement. Inter- and intra-rater reliability exceeded κ = 0.89 across all datasets. For NLS expressive tasks, inter-rater reliability was nearly perfect (κ = 0.986, *p* < 0.001), higher than for PEPS-C expressive tasks (κ = 0.818, *p* < 0.001). Intra-rater reliability for children was also excellent (κ = .974, *p* < 0.001). These high reliability values support Hypothesis 2.2. by ensuring that observed developmental patterns are not confounded by rater variability. Ceiling effects likely lowered internal consistency in adults. Future clinical testing is expected to yield more informative values.

### RQ3: How do performance patterns on the NLS tasks compare to the PEPS-C lexical stress tasks?

#### Concurrent validity.

**Fig 3:**
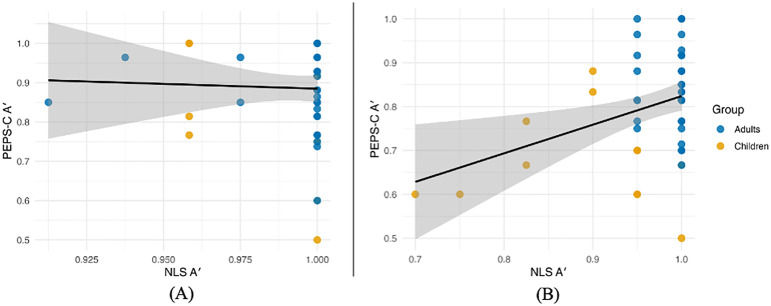
Scatterplots of NLS A’ (x-axis) and PEPS-C A’ (y-axis) values. Presented by participant for (A) expressive and (B) receptive accuracy.

Concurrent validity analyses examined the relationship between the NLS and PEPS-C tasks using Kendall’s tau, chosen to accommodate tied ranks. Across both expressive and receptive tasks, no significant correlations were observed (*expressive*: τ = −0.014, *z* = −0.12, *p* = 0.90; *receptive*: τ = 0.193, *z* = 1.74, *p* = 0.082) (see Fig 3). When analyzed by group, neither adults (*expressive*: τ = 0.036, *z* = 0.26, *p* = 0.80; *receptive*: τ = −0.077, *z* = −0.56, *p* = 0.57) nor children (*expressive*: τ = −0.169, *z* = −0.73, *p* = 0.47; *receptive*: τ = 0.033, *z* = 0.16, *p* = 0.88) showed reliable associations.

These weak correlations should be interpreted with caution. Because participants performed at or near ceiling on the NLS tasks, variability was substantially reduced, and correlation coefficients were correspondingly attenuated. Ceiling-level accuracy produces many tied scores, which limits the sensitivity of rank-based statistics such as Kendall’s tau. Thus, the nonsignificant results potentially reflect a statistical artifact of restricted range rather than evidence against validity. Importantly, adults also showed ceiling accuracy on the NLS tasks but greater variability on the PEPS-C, suggesting that the latter may be more influenced by extraneous task demands (e.g., literacy, orthographic presentation) than by lexical stress ability per se.

Future work will extend these analyses to younger children (under age five) and to clinical populations where greater variability is expected, thereby providing more robust tests of criterion validity. In the current sample, the absence of correlations is best viewed as a limitation of the sample’s restricted range rather than a limitation of the NLS tasks themselves.

#### Expressive task performance.

**Fig 4:**
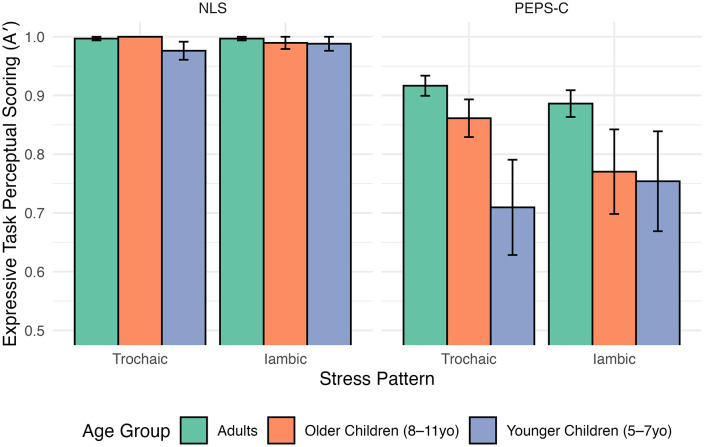
Bar graph depicting A’ values from the perceptual scoring of the expressive NLS and PEPS-C lexical stress tasks. Graph is broken down by stress pattern (trochaic, iambic) and broad age group (adults, older children, younger children). Error bars show standard error.

Means, standard deviations, skewness, and kurtosis for each task (NLS, PEPS-C) are presented in Table 3. Linear mixed models revealed a significant main effect of task: participants achieved higher accuracy on NLS expressive tasks compared to PEPS-C expressive tasks (β = −0.080, *SE* = 0.021, *t*(150.1) = −3.89, *p* < 0.001). No main effect of age group (older: β = 0.003, *SE* = 0.04, *t*(196.1) = 0.09, *p* = 0.933; younger: β = −0.021, *SE* = 0.04, *t*(195.1) = −0.53, *p* = 0.597) was observed, nor were any in*t*eractions significant (all *p* > 0.2), suggesting a limited moderating effect of age group on task accuracy. See Fig 4 for a visual of perceptual scoring as represented by A’.

**Table 3:** Mean, standard deviations, skewness, and kurtosis for A’ values by task, mode, and group.

Task	Mode	Group	*M* A’	*SD* A’	Skewness A’	Kurtosis A’
*NLS*	Expressive	Adults	0.995	0.022	−0.058	−2.69
*NLS*	Expressive	Children	0.989	0.029	−0.068	−2.82
*NLS*	Receptive	Adults	0.990	0.030	−0.033	−2.90
*NLS*	Receptive	Children	0.910	0.114	−0.057	−2.79
*PEPS-C*	Expressive	Adults	0.901	0.126	−0.016	−2.95
*PEPS-C*	Expressive	Children	0.848	0.172	−0.031	−2.92
*PEPS-C*	Receptive	Adults	0.842	0.137	−0.008	−2.97
*PEPS-C*	Receptive	Children	0.701	0.144	−0.005	−2.94

Planned post-hoc comparisons showed that for the NLS tasks, there were no significant differences between adults, older children, and younger children, consistent with expectations that each group can successfully complete the naturalistic NLS expressive tasks. In contrast, for the PEPS-C expressive tasks, adults (trochaic: *M* = 0.917, *SE* = 0.015; iambic: *M* = 0.886, *SE* = 0.015) outperformed younger children (*M* = 0.709, *SE* = 0.055, *t*(198) = 3.64, *p* = 0.001) for trochaic words, but not iambic (*M* = 0.753, *SE* = 0.055, *t*(198) = 2.33, *p* = 0.063). Conversely, adults outperformed older children (*M* = 0.770, *SE* = 0.034, *t*(195) = 3.14, *p* = 0.006) for iambic words, but not trochaic (*M* = 0.861, *SE* = 0.034, *t*(195) = 1.50, *p* = 0.409). These results support Hypothesis 3.1 by showing that the NLS tasks capture lexical stress more consistently across groups, and that the PEPS-C may introduce extraneous literacy and task design demands that may place additional demands on child participants. However, because the child sample was small (n = 15, split across two subgroups), statistical power to detect developmental effects was low. Post-hoc power analyses indicated that only very large effects could be reliably detected, meaning that smaller but theoretically meaningful developmental effects may have gone undetected. Accordingly, the developmental findings should be considered preliminary, with full power analysis details provided in the Supplemental Material.

#### Acoustic correlates.

**Fig 5:**
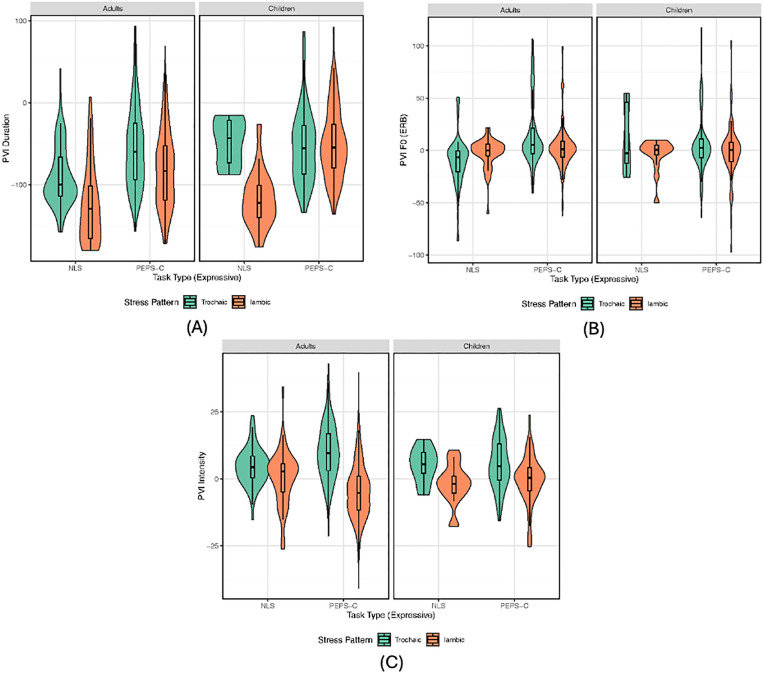
Violin plots showing the distribution of each PVI metric. Plots are (a) top left: duration; (b) top right: f0; and (c) bottom: intensity. Violin plots along with box plots depicting the medians and interquartile ranges broken down by task (NLS, PEPS-C), stress pattern (trochaic = green, iambic = orange), and group (adults, children-5-11yo) in each panel. Only isolated and sentence-final productions are plotted.

Acoustic analyses of accurate expressive productions demonstrated that duration (β = −0.013, *SE* = 0.002, *z* = −8.66, *p* < 0.001) and intensity (β = −0.14, *SE* = 0.01, *z* = −13.06, *p* < 0.001) significantly predicted stress pattern classification, while f0 did not (β = 0.003, *SE* = 0.004, *z* = −0.79, *p* = 0.427). These effects were consistent across models limited to sentence-final productions and across adult and child groups (see Supplemental Material). Linear mixed effects models by PVI metric (Table 4) further confirmed significant item- and participant-level variability, with duration and intensity robustly distinguishing trochaic and iambic stress (Fig 5). These findings support Hypothesis 3.2 and replicate prior cross-linguistic evidence that duration and intensity are the most reliable cues to lexical stress in child speech, while f0 serves multiple prosodic functions and is less stable in early development.

**Table 4:** Linear mixed effects models examining the effects of task, age group, and stress pattern for the three PVI metrics.

	Est.	SE	df	*t*	Pr(<|t|)
**PVI duration**					
*Intercept*	−98.64	11.47	31.11	−8.59	< 0.001^***^
*taskPEPSC*	34.63	15.35	20.85	2.26	= 0.035^*^
*groupChild*	30.67	10.33	362.03	2.97	= 0.003^**^
*stress_patternIamb*	−20.27	2.52	896.67	−8.06	< 0.001^***^
*taskPEPSC:groupChild*	−13.04	9.45	776.78	−1.38	= 0.168
**PVI f0**					
*Intercept*	−3.32	3.17	27.97	−1.05	= 0.305
*taskPEPSC*	16.10	3.32	16.89	4.85	< 0.001^***^
*groupChild*	9.64	6.23	429.17	1.55	= 0.123
*stress_patternIamb*	−9.22	1.88	311.52	−4.91	< 0.001^***^
*taskPEPSC:groupChild*	−14.67	6.37	680.99	−2.31	= 0.021^*^
**PVI intensity**					
*Intercept*	7.23	2.04	35.25	3.55	= 0.001^**^
*taskPEPSC*	1.72	2.49	20.42	0.69	= 0.499
*groupChild*	1.82	2.47	338.90	0.74	= 0.461
*stress_patternIamb*	−13.04	0.64	884.87	−20.39	< 0.001^***^
*taskPEPSC:groupChild*	−1.85	2.30	599.46	−0.81	= 0.421

Fixed factors include task (NLS, PEPS-C), age group (adults, children), stress pattern (trochaic, iambic) and the interaction of task by group for the three PVI metrics (duration, f0, intensity), along with random intercepts for participant and item.

*** = *p* < 0.001, ** = *p* < 0.01, * = *p* < 0.05

#### Receptive task performance.

**Fig 6:**
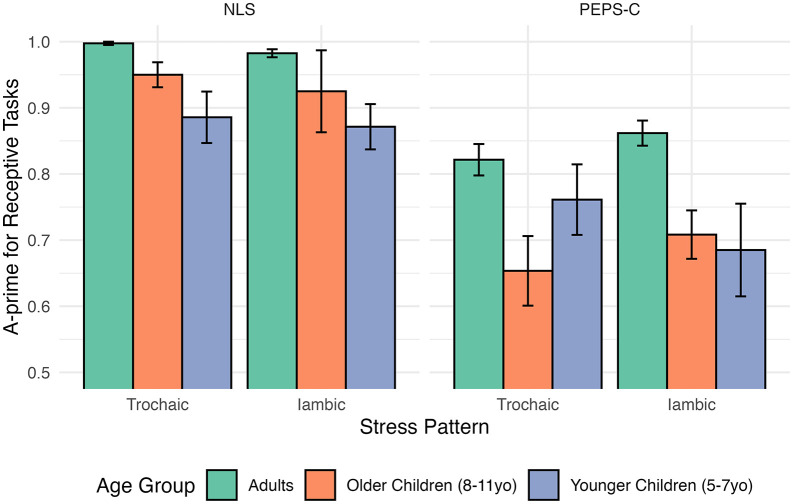
Bar graph depicting A’ values from the perceptual scoring of the receptive NLS and PEPS-C lexical stress tasks. Graph is broken down by stress pattern (trochaic, iambic) and broad age group (adults, older children, younger children). Error bars show standard error.

For receptive tasks, a linear mixed model revealed a significant task by age group interaction showing that older children did not perform as well as adults on the PEPS-C (β = −0.121, *SE* = 0.058, *t*(156.0) = −2.079, *p* = 0.039). A main effect of task indicated that accuracy was higher on NLS than PEPS-C tasks (β = −0.176, *SE* = 0.024, *t*(156.0) = −7.438, *p* < 0.001). Age-related differences were again more apparent in the PEPS-C than in NLS. Adults significantly outperformed younger children on PEPS-C items (β = −0.112, *SE* = 0.044, *t*(207.2) = −2.532, *p* = 0.012), whereas differences be*t*ween adults and children were smaller or absent on NLS tasks (Fig 6). These findings support Hypothesis 3.1 by showing that the NLS receptive tasks reduce age-related disparities compared to the PEPS-C, aligning more closely with developmental expectations.

## General discussion

The current study addressed critical challenges in existing standardized prosodic assessments through the development of novel lexical stress (NLS) tasks. By embedding words in meaningful sentence contexts and removing literacy demands, the NLS tasks provide a more naturalistic and developmentally appropriate assessment of prosody. The results confirmed that the tasks are feasible for children as young as five, demonstrate strong reliability, and align with developmental expectations more closely than the PEPS-C.

### RQ1: Development and ecological validity

The NLS expressive and receptive tasks were explicitly designed to overcome difficulties identified in existing tools [36,44,47]. Unlike the PEPS-C, which elicits isolated productions and requires orthographic knowledge, the NLS tasks elicit words in connected speech and rely on picture-based contrasts [51]. This yields productions that more closely resemble real-world communication. The revised child version increased item complexity (adding three-syllable words) and successfully elicited more sentence-medial productions, reducing prosodic edge effects. Additionally, since lexical stress cues do not remain static and vary due to factors such as sentence position, accentuation, and intonation, having a task that elicits words in multiple contexts allows the NLS task to capture this variability, providing a more comprehensive and ecologically valid profile of ability.

### RQ2: Psychometric properties

The psychometric properties of the NLS tasks were evaluated through analyses of internal consistency and rater reliability, in alignment with recommended guidelines for assessment development [47,86]. Overall, results indicate that the NLS tasks demonstrate strong reliability, with some expected limitations in internal consistency due to sample characteristics.

Internal consistency estimates were low to moderate, particularly in the adult sample. This pattern is best explained by ceiling-level performance, which restricts variability across items and attenuates reliability estimates for short assessments with dichotomous scoring [87]. As such, these values are more appropriately interpreted as a statistical artifact of uniformly high accuracy rather than evidence of weak measurement properties. This interpretation is consistent with prior work showing that internal consistency metrics such as KR-20 are sensitive to restricted range and may underestimate reliability under ceiling conditions. As variability increases in populations with less reliable lexical stress representations (e.g., younger children or clinical groups), these estimates are expected to become more informative.

In contrast, both inter- and intra-rater reliability were excellent, indicating that perceptual judgments of lexical stress can be applied consistently across raters. Notably, reliability was higher for the NLS expressive tasks than for the PEPS-C, suggesting that productions elicited in meaningful contexts may be easier to evaluate than isolated tokens produced under more constrained conditions. These findings support the feasibility of implementing the NLS tasks in clinical and research settings, even when administered and scored by clinicians with typical levels of training in prosody.

The psychometric findings suggest that the NLS tasks provide a stable and reliable measure of lexical stress, while also highlighting the importance of considering sample characteristics when interpreting internal consistency metrics. Future work will extend these analyses to populations with greater expected variability, to further characterize these properties.

Finally, cultural and linguistic considerations are essential for valid assessment. Many standardized tools are limited in their ability to accommodate dialectal variation [88], with alternatives including dynamic assessment and/or language sample analysis. The NLS tasks address this limitation by allowing examiner flexibility in scoring, rather than relying on rigid, automated criteria. This adaptability is crucial for dialects with distinct prosodic profiles, such as African American English (AAE) or Southern English varieties, where initial stress is common in multisyllabic words (e.g., *HOtel*, *NOvember*) [50,89]. By permitting dialect-sensitive scoring, the NLS tasks avoid misidentifying typical variation as impairment and offer a more inclusive approach for assessing prosody across diverse Englishes [88].

### RQ3: Validity and comparative insights

This section integrates perceptual and acoustic findings across tasks to evaluate the validity of the NLS tasks relative to the PEPS-C. The overarching hypothesis was that the NLS and PEPS-C tasks would show unique profiles of lexical stress abilities, with higher accuracy on the NLS tasks than the PEPS-C (Hypothesis 3.1) and acoustic analyses revealing duration and intensity, but not f0, as primary cues to lexical stress, with greater developmental change for iambs than trochees (Hypothesis 3.2). Reduced age-group disparities on the NLS compared to the PEPS-C were also predicted to provide evidence of developmental sensitivity, linking to Hypothesis 2.2 in RQ2. Notably, despite being available for over a decade, the PEPS-C lexical stress subtests have seen limited uptake in the research literature. With recent studies avoiding these tasks, this underscores the need for alternative approaches to assessing lexical stress.

Results across expressive and receptive tasks largely supported these predictions, though with important task-specific differences. NLS tasks yielded higher scores overall, consistent with lexical stress being largely acquired by age five to six years [12], while PEPS-C scores were lower and showed unexpected developmental differences. Acoustic analyses further highlighted contrasts. In the NLS productions, children reflected adult-like patterns for duration and f0 but not intensity, whereas in the PEPS-C, adults and children showed minimal duration and f0 differences but exaggerated intensity contrasts. For receptive tasks, the NLS showed a developmental trend, with adults outperforming the younger children. In comparison, the PEPS-C did not show clear developmental patterns.

#### Expressive tasks (perceptual ratings).

As predicted in Hypothesis 3.1, both adults and children exhibited higher accuracy on the NLS expressive tasks than on the PEPS-C. Adults performed at or near ceiling on NLS, consistent with expectations that lexical stress is mastered by adulthood, and children across the 5–11 age range also showed near ceiling scores, aligning with the hypothesis that minimal developmental change would be observed and that lexical stress is in place by approximately age five when perceptually assessed [12]. In contrast, the PEPS-C introduced apparent developmental differences (e.g., adults outperforming younger children for trochees and older children for iambs), which may reflect task artifacts rather than true differences in lexical stress ability (see RQ1). This pattern supports the hypothesis that the naturalistic NLS task more accurately captures expressive lexical stress ability than the PEPS-C. Importantly, however, the relatively small child sample limits strong conclusions about developmental trajectories. To more directly examine whether the NLS tasks capture developmental change in expressive lexical stress, future work will need to administer the task to larger samples, including younger children (below age five) who are predicted to not yet have acquired full lexical stress contrasts when perceptually assessed.

#### Expressive tasks (acoustic ratings).

Consistent with Hypothesis 3.2, results from the acoustic ratings showed that the PVI measures for duration and intensity were significant predictors differentiating stress patterns across both adults and children, while PVI (of peak) f0 was not. These findings are consistent with previous research showing that f0 does not differentiate trochaic from iambic words in Australian-English speaking children [12], validating that the NLS expressive task elicits productions that replicate known developmental patterns. This may reflect the multiple roles that f0 fulfills in prosody, including marking focus and intonational contrasts. Ballard et al. [12] discussed how recognizing the multiple roles of f0 may evolve across development and observed that children under age five differ in how they use f0 to signal lexical stress, suggesting that future studies should examine younger populations. Research in tonal languages (i.e., Cantonese) further shows that f0 is impacted in disordered speech (e.g., CAS, AOS) [90,91], highlighting the potential value of alternative analytic methods (e.g., growth curve modeling) alongside PVI.

Closer inspection of individual PVI measures revealed task- and age-related differences. PVI duration and PVI f0 (but not intensity) were higher for PEPS-C than NLS productions, with only PVI duration showing a significant age group effect, where children produced larger contrast values than adults. All three PVI measures varied by stress pattern, with lower values in the expected direction for iambs relative to trochees. PVI f0 showed an interaction of task and group, with greater adult-child differences in the PEPS-C task than NLS, though visual inspection suggested minimal group differences overall. Notably, PEPS-C PVI intensity effects were exaggerated for iambic words in adults, likely due to the task’s visual elicitation format (e.g., larger print/circles for stressed syllables), which may have prompted unnatural overemphasis. This issue was not present in the NLS tasks, underscoring its advantage in eliciting more naturalistic productions. Future studies may benefit from having naïve listeners rate production naturalness to further evaluate task effects.

#### Receptive tasks.

Receptive tasks provided a complementary test of Hypotheses 3.1 and 2.2, showing that the NLS task yielded higher A’ scores than the PEPS-C, especially in younger children. This supports the prediction that children (5–11 years old) would perform better on the picture-based NLS task, which reduces literacy demands. An alternative explanation is that the familiarity of the items may have facilitated higher accuracy on the NLS task, though the words were not especially frequent in English. By using pictures instead of orthography, the NLS tasks remove the literacy requirement and provide children with an opportunity to demonstrate their abilities independent of reading skills.

Adults performed at near ceiling for both the NLS and PEPS-C, consistent with mature lexical stress perception. Evidence of developmental sensitivity (Hypothesis 2.2) was partially supported, with trends indicating differences between adults and younger children for both tasks, but not between older children and younger children. The direction of this trend was in the expected direction for the NLS task, with increasing accuracy values from the younger children to older children to adults. However, the small child sample size precludes strong claims about developmental trajectories. For the PEPS-C, younger children unexpectedly outperformed older children for the trochaic stimuli, which are argued to be the earlier acquired perceptual pattern as evidenced from early word learning [92,93]. While intriguing, this pattern may reflect task-specific factors or variability in the small sample rather than robust differences. In contrast, the NLS pattern more closely follows an anticipated developmental direction, suggesting that it more accurately may capture lexical stress perception than the PEPS-C, which likely reflects literacy and task-format demands discussed earlier.

Taken together, these findings support Hypothesis 3.1 by showing that NLS tasks yield higher accuracy than the PEPS-C, particularly in children, and partially support Hypothesis 2.2 by suggesting that developmental sensitivity may be more apparent in receptive than in expressive tasks, though larger samples will be needed to clarify these patterns. Alongside the expressive findings, these receptive results highlight the different profiles captured by the NLS and PEPS-C tasks, setting the stage for a broader discussion of their validity as prosodic assessments.

#### Implications for validity measures.

Construct validity refers to whether a test truly measures the theoretical construct it is intended to capture, in this case lexical stress ability, which was examined by testing whether tasks reflected expected developmental patterns. The near-ceiling performance on the NLS expressive task aligns with prior evidence of early lexical stress mastery [15,16]. While this ceiling accuracy supports construct validity, it also restricts variability, which in turn limits the usefulness of certain psychometric analyses (e.g., internal consistency, correlations). Thus, the nonsignificant correlations with the PEPS-C should be interpreted as reflecting a restricted range rather than evidence against validity.

Criterion validity was addressed via concurrent comparison with the PEPS-C [39,40]. No significant correlations were found for either the expressive or the receptive tasks. The PEPS-C expressive task may rely heavily on reading skills (four younger children could not complete it), and its elicitation format likely encouraged unnatural overemphasis, as reflected in inflated intensity values. Additionally, aspects of the PEPS-C administration and stimulus design may compromise expressive validity, including potential priming effects from task order and intonational contours that alter the percept and acoustics of the word. Together, these issues limit the utility of the PEPS-C for expressive lexical stress assessment.

For receptive tasks, weak positive trends suggested some overlap between the NLS and the PEPS-C. However, adults performed variably on the PEPS-C despite ceiling performance on the NLS task, raising doubts about the PEPS-C’s ability to measure adult lexical stress perception accurately. While the NLS and PEPS-C demonstrate similarities in terms of structure and types of words assessed, orthographic presentation and unfamiliar lexical sets remain limitations for the PEPS-C. By contrast, the NLS task offers a more refined and developmentally appropriate tool, particularly for children, and demonstrates stronger construct validity.

### Limitations and future directions

For this initial development of the NLS tasks, there were limitations in terms of the sample and the design. First, a homogenous sample from New England was used in order to make comparisons across groups. As prosody is diverse and varies by dialect, it is important that future work includes a more diverse dialectal sample [50]. This would impact scoring by providing a broader baseline along with guidance for how to approach the scoring of items that may differ across dialects.

Additionally, there was a relatively small sample of children included in the age range from 5 to 11 years old. This range was selected partially to be able to compare to the PEPS-C tasks, which are designed to begin at age four. Since the PEPS-C requires reading for its lexical stress tasks, this younger age group was difficult to assess. In the end, the age range sampled provides a limited view of the expected developmental progression for the NLS tasks since it is most likely before age five where children demonstrate more variation, particularly in terms of perceptually and acoustically assessed expressive productions [12]. Future work plans to increase the sample size and also lower the age range (< 5 years old) to assess how younger children perform on the NLS tasks. Currently, there are no prosodic assessments that are designed for children younger than four years old.

Although adults and older children performed at or near ceiling on the NLS tasks, which was expected based on the developmental literature, this naturally limits the ability to detect fine-grained age differences across the 5–11-year-old age range. We argue that this reflects work showing that lexical stress is largely in place by age five, and that apparent age effects on the PEPS-C are more likely due to literacy and task design demands rather than genuine developmental differences. Detailed post-hoc sensitivity analyses (see Supplemental Material) further confirm that the current child sample was only sufficiently powered to detect large effects, highlighting the need for larger and more diverse samples in future work.

There are aspects in the design and procedure of the NLS tasks that may be adapted for future versions. For the design, one potential limitation was the stimuli utilized in the NLS tasks. Although internal consistency increased from the adult to the child version of the expressive task (due partially to the addition of more complex stimuli), the overall number of stimuli remains limited, constraining reliability. To continue to improve the internal consistency of items, the number of stimuli could be further increased. For the NLS receptive task, however, options for improvement are more constrained because of the limited ways the task can be structured to assess lexical stress perception. One possible approach would be to increase the number of items, but this also risks lowering familiarity of the stimuli. As such, it is difficult to disentangle whether lower performance reflects true challenges with lexical stress perception or simply reduced familiarity with the words. Finally, although all children completed a brief vocabulary review to align the intended labels with the stimuli, the small set of English noun-verb pairs that vary by lexical stress necessarily included items with higher age-of-acquisition values. Thus, vocabulary knowledge remains a potential confound, a general limitation of prosodic assessments of this type.

Another limitation was in the presentation of the tasks, which was set with the PEPS-C first and the NLS second due to the length of the PEPS-C tasks and difficulty completing them during piloting. Still, using one order may have increased the risk of task effects and fatigue. However, since the NLS tasks were completed by all participants and their performance was quite high, this suggests that fatigue most likely was not an influencing factor. Task effects due to stimuli exposure (which were different across the two approaches) or familiarity to testing could still have been present and future steps in the development of these tasks aim to counter-balance the order of presentation.

Lexical stress is a phonological construct and can be difficult to reduce to only particular acoustic metrics. Here, we show how the acoustic measurements relate to production, and how this may vary based on a reading task versus one that is more spontaneously produced. For perception, depending on the position and context, the cues to lexical stress will vary naturally. In English, vowel reduction in the unstressed syllable is a particularly important cue that helps listeners identify lexical stress placement. In some cases, a task may be deliberately designed to control the cues to lexical stress, for instance, the PEPS-C sometimes avoids vowel reduction in weak syllables so that this cue is not available to the listener. Here, we take a more naturalistic approach and use words that may vary in vowel quality between syllables, as is expected in mainstream US English. In the development of these NLS tasks, a preference was made for the examination of lexical stress as it occurs naturally, but this preference may differ depending on the goals of the examiner.

An important consideration when developing a new assessment is whether it measures ability or performance. Ability is typically assessed under highly controlled conditions to estimate potential under ideal conditions. In contrast, performance reflects functional communication in real-world contexts, where factors such as fatigue, emotional state, and social-pragmatic demands influence production. Just as an articulation assessment would not evaluate /p/ only in simple onset position, eliciting lexical stress exclusively in isolated words limits the evaluation of how stress is produced across linguistic contexts. Since prosody is inherently sensitive to pragmatic context and communicative intent [94,95], the NLS tasks were designed to balance between meaningful context (performance) and structured elicitations in contexts devoid of meaning (ability). The semi-naturalistic expressive task elicits target words in spontaneous speech without imposing additional social demands, making the NLS tasks uniquely suited for capturing complex prosodic skills as they manifest in real-world communication.

### Using the NLS tasks in the clinic

Given the potential clinical utility of the NLS tasks, it is important to highlight how they can be feasibly implemented in real-world contexts. Although this study employed a controlled laboratory setup (e.g., two speakers, detailed acoustic analyses) to enable rigorous testing, the expressive NLS task itself is simple and clinically accessible. In practice, a speech-language pathologist would need only the picture stimuli, a short set of instructions, and the scoring rubric. The task requires no literacy and can be completed quickly, making it feasible in school or clinic contexts. To facilitate clinical use, we provide supplemental materials including stimulus examples, scoring guidelines, and group-level reference values, which can serve as a starting point for clinicians interested in incorporating the NLS tasks into practice. Similarly, the receptive NLS tasks can be administered on a standard computer with built-in speakers in a quiet setting, with pictures and mainstream US English target stimuli also included in the Supplemental Material. In this way, the NLS tasks balance experimental rigor and clinical feasibility, advancing the broader goal of developing ecologically valid tools for assessing prosody in both research and practice.

## Conclusion

The findings from this study contribute to the development of prosodic assessment by introducing novel lexical stress (NLS) tasks, examining their psychometric properties, and evaluating performance in neurotypical adults and typically developing children aged 5–11 years in comparison to the PEPS-C lexical stress tasks [40]. Given that lexical stress is a diagnostic feature in several speech disorders, including dysarthria and AOS/CAS [25,96], improving how this aspect of prosody is assessed in more naturalistic contexts is clinically important. In addition to perceptual judgments, acoustic analyses provided objective, fine-grained insight into lexical stress production. Future work will focus on refining the NLS tasks, extending their use with children under age five (where the most developmental variability is expected), and evaluating their sensitivity to prosodic differences in clinical populations. Although NLS performance approached ceiling in neurotypical adults and children, this pattern is consistent with developmental evidence that lexical stress is mastered by early childhood. By integrating meaningful context with semi-structured elicitation, the NLS tasks represent a promising step toward more ecologically valid prosodic assessment and provide a foundation for advancing both clinical tools and theoretical understanding of prosodic development.

## Supporting information

S1 Appendix This contains information about the stimuli. (PDF)

S2 File This contains additional analyses and the clinician quick guide. (PDF)

S3 Files This zipped folder contains the information related to each task including the sound files, picture stimuli, record forms, and task procedure. (ZIP)
