# Supplementary material for: Understanding development of Mainstream US English lexical stress using semi-naturalistic stimuli
Source: PLoS One. 2026 Apr 29;21(4):e0345745. doi: 10.1371/journal.pone.0345745 (PMC13128110; doi:10.1371/journal.pone.0345745)
Supplement: S1 Appendix — (PDF) [file pone.0345745.s002.pdf]

S1 Appendix
Stimuli lists for the NLS and PEPS-C lexical stress tasks
in “Understanding development of Mainstream US English lexical stress using semi-naturalistic stimuli”

Description: This Appendix presents details on all receptive (Table A.1) and expressive stimuli (Table A.3) in the Novel Lexical Stress (NLS) tasks and the Profiling Elements of Prosody in Speech Communication (PEPS-C) tasks. Two-sided t-tests are provided comparing features of stimuli from the two tasks (NCF vs. PEPS-C) (Tables A.2 and A.4). Finally, the accuracy per stimulus item is provided in Table A.5.

*A supplemental folder containing the sound files for the receptive stimuli is attached as “NLS-ReceptiveTask-AudioFiles.zip” along with the experimental presentation that includes their visual stimuli.

Table A.1. Receptive stimuli for the NLS and PEPS-C Tasks

Item	IPA	CV	Syll Stress	Syn. Class	Word Freq (COCA)	Word Freq (NOW)$^{+}$	AoA Kuperman *M (SD)*	MCDI Form B (W&S)	Phonological Neighborhood Density	% Sonorant	Image -ability	Concrete -ness
NLS Receptive												
CONverse	/ˈkɔnvɚs/	CVCCVC	1	N	625	26188	12.00 (2.90)	N	2	50.00		
conVERSE	/kənˈvɝs/	CVCCVC	2	V	2586	26188	12.00 (2.90)	N	5	50.00		
PROject	/ˈprɑdʒɛkt/	CVCVCC	1	N	177,076	5149983	9.00 (3.97)	N	2	42.86		
proJECT	/prəˈdʒɛkt/	CVCVCC	2	V	17755	5149983	9.00 (3.97)	N	5	42.86		
PROduce	/ˈprodus/	CCVCVC	1	N	6051	1428794	7.75 (3.11)	N	1	50.00	396	432
proDUCE	/prəˈdus/	CCVCVC	2	V	154082	1428794	7.75 (3.11)	N	4	50.00		
PREsent	/ˈprɛzɪnt/	CCVCVCC	1	N	37433	2509478	4.83 (2.01)	Y	1	57.14	481	389
preSENT	/prəˈzɛnt/	CCVCVCC	2	V	111731	2509478	4.83 (2.01)	N	1	57.14		
REcord	/ˈrɛkɚd/	CVCVC	1	N	187057	3858739	6.30 (2.60)	N	3	60.00	591	558
reCORD	/rəˈkɔrd/	CVCVC	2	V	51375	3858739	6.30 (2.60)	N	1	60.00		
OBject	/ˈɑbdʒɛkt/	VCCVCC	1	N	73304	325084	7.94 (3.04)	N	3	40.00	408	487
obJECT	/əbˈdʒɛkt/	VCCVCC	2	V	13303	325084	7.94 (3.04)	N	6	50.00		
		Mean (SD)			**69364 (70571)**	**2216377 (1925121)**	**7.97 (2.33)**	**8.33%**	**2.83 (1.80)**	**50.00 (7.41)**	**469.00 (89.59)**	**466.50 (73.00)**

*PEPS-C Lexical Stress Receptive**											
DIffer	/ˈdɪfɚ/	CVCV	1	N	13787	9.00 (2.58)	N	16	50.00		
deFER	/dəˈfɝ/	CVCV	2	V	2237	11.88 (2.26)	N	12	50.00		
DIScard	/ˈdɪskɑrd/	CVCCVCC	1	N	610	10.42 (3.59)	N	2	42.86		
disCOUNT	/dɪsˈkount/	CVCCVCC	2	V	4828	9.50 (2.83)	N	1	42.86		
IMport	/ˈɪmport/	VCCVCC	1	N	12336	10.37 (2.75)	N	2	66.67	361	320
imPORT	/ɪmˈport/	VCCVCC	2	V	8974	10.37 (2.75)	N	2	66.67	361	320
imPRINT	/ɪmˈprɪnt/	VCCCVCC	1	V	1214	11.35 (4.06)	N	1	71.42	332	421
INsight	/ˈɪnsɑit/	VCCVC	2	N	18701	10.95 (3.96)	N	9	60.00	329	270
inCITE	/ɪnˈsɑit/	VCCVC	1	V	3126	13.12 (4.43)	N	9	60.00		
INcrease	/ˈɪnkris/	VCCCVC	2	N	71248	7.42 (2.81)	N	1	66.67	356	315
inCREASE	/ɪnˈkris/	VCCCVC	1	V	137709	7.42 (2.81)	N	1	66.67	356	315
inDENT	/ɪnˈdɛnt/	VCCVCC	2	V	268	10.26 (2.88)	N	2	66.67		
INsert	/ˈɪnsɚt/	VCCVC	1	N	2580	8.20 (2.42)	N	5	60.00		
inSERT	/ɪnˈsɝt/	VCCVC	2	V	14249	8.20 (2.42)	N	5	60.00		
INsult	/ˈɪnsəlt/	VCCVCC	1	N	9298	8.63 (2.43)	N	3	66.67	477	375
INtern	/ˈɪntɚn/	VCCVC	2	N	5040	14.00 (3.57)	N	5	80.00		
in TURN**	/ɪn ˈtɝn/	CVCV	1	AdvP	21240		N	16	80.00		
		Mean (SD)			**19261 (34747)**	**10.14 (1.97)**	**0.00%**	**4.75 (4.51)**	**62.19 (10.91)**	**367.43 (50.14)**	**333.71 (49.09)**

Notes:
*The PEPS-C receptive task does not always include pairs of words that vary by syntactic class and lexical stress.
**The PEPS-C receptive task lists '*in TURN'* two times (listed once here in this list) and includes one item that is two words ('*in turn'*) instead of one and is considered an adverbial phrase (AdvP).
+The NOW corpus does not differentiate between nouns and verbs that differ by lexical stress only. Thus, the numbers reflected here are the same for each word in the pair.

Table A.2. Two-sided independent samples t-tests comparing the NLS receptive stimuli to the PEPS-C across a variety of variables of interest. Variables, t-value, degrees of freedom, means and standard deviations (sd) for each task, 95% confidence intervals of the difference, and p-values reported. Equal variances tested with Levene's Test for Equality of Variances. Equal variances tests reported unless noted with a $^{+}$ which indicates that Levene's test was < 0.05 and equal variances could not be assumed and thus the unequal variances version of the t-test was conducted.

Variable of Interest	Task	*M(SD)*	t-value	df	95% CI	Two-sided *p*-value
**Word frequency-COCA corpus**$^{+}$	*NLS*	69364 (70571)	2.273	14.79	[2054, 97153]	**= 0.017***
	PEPS-C	19261 (34747)				
**Word frequency-NOW corpus**	*NLS*	2216377 (1925121)				
	PEPS-C					
**Phonological neighborhood density**$^{+}$	*NLS*	2.83 (1.80)	-1.54	20.76	[-4.49, 0.67]	= 0.138
	PEPS-C	4.75 (4.51)				
**Percent Sonorant**	*NLS*	50.00 (7.41)	-3.39	27	[-0.20, -0.05]	**= 0.002****
	PEPS-C	62.19 (10.91)				
**Age of Acquisition - Mean** (in years)	*NLS*	7.97 (2.33)	-2.61	26	[-3.75, -0.44]	**= 0.015***
	PEPS-C	10.14 (1.97)				
**Age of Acquisition - SD** (in years)	*NLS*	2.94 (0.62)	-0.39	26	[-.60, -0.41]	= 0.700
	PEPS-C	3.03 (0.67)				
**Imageability** (range = 100-700; 700 = higher imageability)	*NLS*	469.00 (25.85)	2.46	9	[8.04, 195.10]	**= 0.036***
	PEPS-C	367.43 (50.14)				
**Concreteness** (range = 100-700; 700 = more concrete)	*NLS*	466.50 (73.00)	3.64	9	[50.31, 215.26]	**= 0.005****
	PEPS-C	333.71 (49.09)				

Table A.3. Expressive stimuli for the NLS and PEPS-C Tasks

Item	IPA	CV	Syll Stress	# of Syllables	Word Freq (COCA)	Word Freq (NOW)$^{+}$	AoA Kuperman *M (SD)*	MCDI Form B (W&S)	Phonological Neighborhood Density	% Sonorant	Image -ability	Concrete -ness
NLS Expressive												
cookie	/ˈkʊki/	CVCV	1	2	10687	1045844	3.37 (1.86)	Y	14	50.00	600	634
chicken	/ˈtʃɪkən/	CVCVC	1	2	47083	530215	3.26 (1.73)	Y	4	60.00	619	614
turtle	/ˈtɝɾəl/	CVCVC	1	2	7094	70857	4.17 (1.58)	Y	9	60.00	564	644

monkey	/ˈmʌŋki/	CVCCV	1	2	11442	100039	4.21 (1.40)	Y	10	80.00	588	566
bubbles	/ˈbʌbəlz/	CVCVCC	1	2	6226	83320	3.79 (1.78)	Y	8	50.00		
balloon	/bəˈlun/	CVCVC	2	2	8019	117897	4.37 (1.95)	Y	7	80.00	583	623
canoes	/kəˈnuz/	CVCVC	2	2	1237	11002	6.63 (1.54)	N	1	60.00		
giraffe	/dʒəˈræf/	CVCVC	2	2	1139	19847	5.00 (3.15)	Y	1	50.00		
guitar	/gɪˈtɑr/	CVCVC	2	2	18092	255267	5.32 (1.20)	N	1	60.00		
raccoon	/ræˈkun/	CVCVC	2	2	1609	16458	6.79 (3.79)	N	1	80.00		
butterfly	/ˈbʌɾɚflaɪ/	CVCVCCV	1	3	6293	85876	3.67 (2.14)	Y	1	57.14	624	593
elephant	/ˈɛləfɪnt/	VCVCVCC	1	3	9243	176547	4.80 (1.74)	Y	1	71.43	616	628
banana	/bəˈnænə/	CVCVCV	2	3	7758	117272	3.78 (1.17)	Y	2	83.33	644	633
spaghetti	/spəˈgɛɾi/	CCVCVCV	2	3	3953	38035	4.33 (1.19)	Y	1	42.86		
		***Mean* (*SD*)**			**9991 (11607)**	**190605 (280364)**	**4.54 (1.09)**	**78.57%**	**4.36 (4.40)**	**63.20 (13.40)**	**604.75 (25.85)**	**616.88 (25.68)**
PEPS-C Lexical Stress Expressive												
IMprint	/ˈɪmprɪnt/	VCCCVCC	1	N	2872		11.35 (4.06)	N	1	71.42	332	421
imPRINT	/ɪmˈprɪnt/	VCCCVCC	2	V	1214		11.35 (4.06)	N	1	71.42	332	421
DIScard	/ˈdɪskɑrd/	CVCCVCC	1	N	610		10.42 (3.59)	N	2	42.86		
disCARD	/dɪsˈkɑrd/	CVCCVCC	2	V	8245		10.42 (3.59)	N	1	42.86		
INsult	/ˈɪnsəlt/	VCCVCC	1	N	9298		8.63 (2.43)	N	3	66.67	477	375
inSULT	/ɪnˈsʌlt/	VCCVCC	2	V	8816		8.63 (2.43)	N	3	66.67	477	375
DIffer	/ˈdɪfɚ/	CVCV	1	N	13787		9.00 (2.58)	N	12	50.00		
deFER	/dəˈfɝ/	CVCV	2	V	2237		11.88 (2.26)	N	16	50.00		
INcrease	/ˈɪnkris/	VCCCVC	1	N	71248		7.42 (2.81)	N	1	66.67	356	315
inCREASE	/ɪnˈkris/	VCCCVC	2	V	137709		7.42 (2.81)	N	1	66.67	356	315
DIscount	/ˈdɪskɑunt/	CVCCVCC	1	N	13415		9.50 (2.83)	N	2	42.86		
disCOUNT	/dɪsˈkɑunt/	CVCCVCC	2	V	4828		9.50 (2.83)	N	1	42.86		
INsert	/ˈɪnsɚt/	VCCVC	1	N	2580		8.20 (2.42)	N	5	60.00		
inSERT	/ɪnˈsɝt/	VCCVC	2	V	14249		8.20 (2.42)	N	5	60.00		

INdent	/ˈɪndɛnt/	VCCVCC	1	N	60	10.26 (2.88)	N	2	66.67		
inDENT	/ɪnˈdɛnt/	VCCVCC	2	V	268	10.26 (2.88)	N	2	66.67		
INsight	/ˈɪnsɑit/	VCCVC	1	N	18701	10.95 (3.96)	N	9	60.00		
inCITE	/ɪnˈsɑit/	VCCVC	2	V	3126	13.12 (4.43)	N	9	60.00	329	270
		Mean			**17403**	**9.81**	**0.00%**	**4.22**	**58.57**	**379.86**	**356.00**
		(SD)			**(53498)**	**(1.58)**		**(4.41)**	**(10.48)**	**(67.30)**	**(57.63)**

+The NOW corpus does not differentiate between nouns and verbs that differ by lexical stress only. Thus, the numbers reflected here are the same for each word in the pair.

Table A.4. Two-sided independent samples t-tests comparing the NLS expressive stimuli to the PEPS-C across a variety of variables of interest. Variables, t-value, degrees of freedom, means and standard deviations (sd) for each task, 95% confidence intervals of the difference, and p-values reported. Equal variances tested with Levene's Test for Equality of Variances. Equal variances tests reported unless noted with a + which indicates that Levene's test was < 0.05 and equal variances could not be assumed and thus the unequal variances version of the t-test was conducted.

Variable of Interest	Task	*M(SD)*	t-value	df	95% CI	Two-sided *p*-value
**Word frequency-COCA corpus**	*NLS*<br>*PEPS-C*	9991 (11607)<br>17403 (43126)	-0.776	30	[-26917, 12092]	= 0.444
**Word frequency-NOW corpus**	*NLS*<br>*PEPS-C*	190605 (280364)				
**Phonological neighborhood density**	*NLS*<br>*PEPS-C*	4.36 (4.40)<br>4.22 (4.41)	0.086	30	[-3.08, 3.34]	= 0.932
**Percent Sonorant**	*NLS*<br>*PEPS-C*	63.14 (13.33)<br>58.59 (10.78)	1.053	29	[-4.30, 13.04]	= 0.301
**Age of Acquisition - Mean** (in years)	*NLS*<br>*PEPS-C*	4.54 (1.09)<br>9.81 (1.58)	-10.62	30	[-6.28, -4.26]	**< 0.001*****
**Age of Acquisition - SD** (in years)	*NLS*<br>*PEPS-C*	1.94 (0.96)<br>3.07 (0.69)	-3.87	30	[-1.72, -0.53]	**< 0.001*****
**Imageability+** (range = 100-700; 700 = higher imageability)	*NLS*<br>*PEPS-C*	604.75 (25.85)<br>379.86 (67.03)	8.32	7.54	[161.89, 287.89]	**< 0.001*****
**Concreteness+** (range = 100-700; 700 = more concrete)	*NLS*<br>*PEPS-C*	616.88 (25.68)<br>356.00 (57.63)	11.05	8.06	[206.52, 315.23]	**< 0.001*****

Table A.5. Accuracy rates by item across task (NLS, PEPS-C) and mode (expressive, receptive).

Task	Mode	Group	Item	Accuracy_Rate
NLS	Exp	A	balloon	1
NLS	Exp	A	bubbles	0.975
NLS	Exp	A	canoes	1
NLS	Exp	A	chicken	1
NLS	Exp	A	cookie	1
NLS	Exp	A	giraffe	1
NLS	Exp	A	guitar	1
NLS	Exp	A	monkey	1
NLS	Exp	A	raccoon	0.975
NLS	Exp	A	turtle	1
NLS	Exp	C	balloon	0.933333333
NLS	Exp	C	banana	1
NLS	Exp	C	bubbles	1
NLS	Exp	C	butterfly	0.933333333
NLS	Exp	C	canoes	1
NLS	Exp	C	chicken	1
NLS	Exp	C	cookie	1
NLS	Exp	C	elephant	1
NLS	Exp	C	giraffe	1
NLS	Exp	C	guitar	1
NLS	Exp	C	monkey	1
NLS	Exp	C	raccoon	0.933333333
NLS	Exp	C	spaghetti	1
NLS	Exp	C	turtle	0.933333333
NLS	Rec	A	converse	0.987341772
NLS	Rec	A	object	1
NLS	Rec	A	present	1

NLS	Rec	A	produce	0.925
NLS	Rec	A	project	1
NLS	Rec	A	record	0.9875
NLS	Rec	C	converse	0.7
NLS	Rec	C	object	0.966666667
NLS	Rec	C	present	0.933333333
NLS	Rec	C	produce	0.733333333
NLS	Rec	C	project	0.933333333
NLS	Rec	C	record	0.966666667
PEPSC	Exp	A	defer	0.923076923
PEPSC	Exp	A	differ	0.942307692
PEPSC	Exp	A	discard	0.807692308
PEPSC	Exp	A	discount	0.846153846
PEPSC	Exp	A	incite	0.8
PEPSC	Exp	A	increase	0.844155844
PEPSC	Exp	A	indent	0.833333333
PEPSC	Exp	A	insert	0.858974359
PEPSC	Exp	A	insight	0.875
PEPSC	Exp	A	insult	0.818181818
PEPSC	Exp	C	defer	0.066666667
PEPSC	Exp	C	differ	0.466666667
PEPSC	Exp	C	discard	0.366666667
PEPSC	Exp	C	discount	0.4
PEPSC	Exp	C	incite	0.266666667
PEPSC	Exp	C	increase	0.5
PEPSC	Exp	C	indent	0.4
PEPSC	Exp	C	insert	0.366666667
PEPSC	Exp	C	insight	0.666666667
PEPSC	Exp	C	insult	0.466666667

PEPSC	Rec	A	converse	1
PEPSC	Rec	A	defer	0.925
PEPSC	Rec	A	differ	0.9
PEPSC	Rec	A	discard	0.7
PEPSC	Rec	A	discount	0.925
PEPSC	Rec	A	import	0.675
PEPSC	Rec	A	incite	0.475
PEPSC	Rec	A	increase	0.75
PEPSC	Rec	A	insert	0.825
PEPSC	Rec	A	insight	0.615384615
PEPSC	Rec	A	insult	0.6
PEPSC	Rec	A	intern	0.85
PEPSC	Rec	A	in turn	0.825
PEPSC	Rec	C	defer	0.266666667
PEPSC	Rec	C	differ	0.733333333
PEPSC	Rec	C	discard	0.466666667
PEPSC	Rec	C	discount	0.533333333
PEPSC	Rec	C	import	0.633333333
PEPSC	Rec	C	incite	0.733333333
PEPSC	Rec	C	increase	0.533333333
PEPSC	Rec	C	insert	0.433333333
PEPSC	Rec	C	insight	0.666666667
PEPSC	Rec	C	insult	0.466666667
PEPSC	Rec	C	intern	0.4
PEPSC	Rec	C	in turn	0.433333333
