# Supplementary material for: Understanding development of Mainstream US English lexical stress using semi-naturalistic stimuli
Source: PLoS One. 2026 Apr 29;21(4):e0345745. doi: 10.1371/journal.pone.0345745 (PMC13128110; doi:10.1371/journal.pone.0345745)
Supplement: S2 File — (PDF) [file pone.0345745.s003.pdf]

S2 Supplemental Material
for "Understanding development of Mainstream US English lexical stress using semi-naturalistic stimuli"

<u>The following is included in the Supplemental Material:</u>

Topic	Page
1. Clinician Quick Guide for Using the NLS Tasks	2
2. Statistical Model Details & R Code	4
3. Sensitivity (Post-Hoc) Power Analyses	11

Quick Guide: Using the Natural Lexical Stress (NLS) Tasks in Clinical Practice

Overview: This guide is designed as practical reference for speech-language pathologists who wish to administer the NLS tasks. It highlights the essential materials, procedures, and scoring needed to implement the expressive and receptive tasks in a clinical or school setting.

NLS Expressive Task

Materials Needed

- Picture stimuli (provided in Supplemental Material)
- Scoring sheet/rubric
- Recording device (optional for later analysis; not required clinically)

Administration

1. Present the child with the stimulus image
2. Prompt: "Tell me a story about the picture."
3. Target words are embedded in natural speech through picture descriptions.

Scoring

- Using your ear, mark stress pattern as:
  - 1 = first syllable stress
  - 2 = second syllable stress
  - A = ambiguous/unscorable
- For clinical use, ambiguous responses can be converted to "incorrect"
- It may be helpful to also note if the word was produced in sentence-medial or sentence-final position; it may be the case that this is a mediating factor for accurate production
- Reference values for typical performance across age groups are provided

Clinical Notes

- Literacy is not required
- Task is brief (5-10 minutes)
- Produces naturalistic samples suitable for both perceptual scoring and optional acoustic follow up

---

NLS Receptive Task

Materials Needed

- Computer or table with built-in speakers
- Picture pairs (provided in Supplemental Material as a PowerPoint)
- Response sheet

Administration

1. Play the PowerPoint presentation, the sounds should automatically play as the slides are advanced
    a. There is a vocabulary review to start, followed by the test stimuli
    b. The vocabulary review is intended to familiarize the client, not remove items
2. Ask the participant to point to or select the picture that matches the word they hear

Scoring

- 1 = correct picture selected
- 2 = incorrect picture selected
- Accuracy can be calculated per task or collapsed into a total score
- Group-level reference values are provided

Clinical Notes

- Designed to reduce literacy demands
- Developmentally appropriate for children as young as 5 years old
- Can be completed in ~5 minutes

Reference Values (Summary)

- *Adults*: Near ceiling performance on both tasks
- *Children (ages 5-11 years)*: High accuracy on expressive task, more variability for the receptive task for the younger 5–7-year-old children
- See Table 3 for overall accuracy scores and Supplemental Table A.5 for accuracy by item for adults vs. children

Additional statistical models and R code
Description: Additional lmm/glm models are referenced in the paper, but due to space and study goals, they are presented here in the supplemental material.

1. R code and statistical model for only final/isolated target words that were scored as accurate (in Aim 3 of Statistical Analyses section): A generalized linear mixed-effects model was fitted to all accurately scored data and is presented in the paper. Here, we present a second glmer model that limited the response variable to only the *final/isolated words* that were accurately scored. This model mirrored the results of the model presented in the paper and is presented here:

R Code:
```
glmer(stress_pattern ~ pvi_dur + pvi_f0_erb + pvi_int + (1 | ID),
           data = df_exp_finalonly %>% filter(accuracy == "1"),
           family = binomial(link = "logit"))

#singularity, run without random effect structure
glm(stress_pattern ~ pvi_dur + pvi_f0_erb + pvi_int,
           data = df_exp_finalonly %>% filter(accuracy == "1"),
           family = binomial(link = "logit"))
```
Model Summaries:
```
Generalized linear mixed model fit by maximum likelihood (Laplace
Approximation) ['glmerMod']
Family: binomial  ( logit )
Formula: stress_pattern ~ pvi_dur + pvi_f0_erb + pvi_int + (1 | ID)
Data: df_exp_finalonly %>% filter(accuracy == "1")

   AIC      BIC      logLik   deviance   df.resid
   613.6    636.1   -301.8    603.6        655

Scaled residuals:
Min        1Q      Median    3Q         Max
-4.7460   -0.5531  0.0649    0.5603    20.9157

Random effects:
Groups Name            Variance      Std.Dev.
ID     (Intercept)     1.749e-14     1.323e-07
Number of obs: 660, groups:  ID, 53

Fixed effects:
              Estimate   Std. Error      z value     Pr(>|z|)
(Intercept)  -3.082e-01  1.934e-01      -1.593      0.111049
pvi_dur       -7.886e-03  2.157e-03      -3.656      0.000256 ***
pvi_f0_erb     1.528e-05  4.595e-03      0.003       0.997347
pvi_int      -1.732e-01  1.455e-02      -11.905     < 2e-16 ***
---
Signif. codes:  0 '***' 0.001 '**' 0.01 '*' 0.05 '.' 0.1 ' ' 1
SIMPLIFIED MODEL:
glm(formula = stress_pattern ~ pvi_dur + pvi_f0_erb + pvi_int,
    family = binomial(link = "logit"), data = df_exp_finalonly %>%
        filter(accuracy == "1"))
```

```
Correlation of Fixed Effects:
           (Intr) pvi_dr pv_f0_
pvi_dur     0.846
pvi_f0_erb -0.145 -0.109
pvi_int    -0.045  0.058 -0.172
optimizer (Nelder_Mead) convergence code: 0 (OK)
boundary (singular) fit: see help('isSingular')

Coefficients:
             Estimate    Std. Error  z value   Pr(>|z|)
(Intercept)  -3.082e-01  1.934e-01   -1.593    0.111049
pvi_dur      -7.886e-03  2.157e-03   -3.656    0.000256 ***
pvi_f0_erb    1.528e-05  4.595e-03    0.003    0.997347
pvi_int      -1.732e-01  1.455e-02   -11.906   < 2e-16 ***
---
Signif. codes:  0 '***' 0.001 '**' 0.01 '*' 0.05 '.' 0.1 ' ' 1

(Dispersion parameter for binomial family taken to be 1)

Null deviance: 914.95  on 659  degrees of freedom
Residual deviance: 603.60  on 656  degrees of freedom
(117 observations deleted due to missingness)
AIC: 611.6
Number of Fisher Scoring iterations: 5
```

2. R code and statistical model for only accurately scored target words, but separated by task (in Aim 3 of Statistical Analyses section): Two generalized linear mixed-effects models were fitted to all accurately scored data, but separated by task (NLS and PEPS-C). These models mirror the results of the model presented in the paper, which combines the two tasks together:

R Code:

```
#For only accurate productions from NLS:
glmer(stress_pattern ~ pvi_dur + pvi_f0_erb + pvi_int + (1 | ID),
           data = df_exp %>% filter(accuracy == "1", task == "NLS"),
           family = binomial(link = "logit"))

#due to singularity, run without random effect structure
glm(stress_pattern ~ pvi_dur + pvi_f0_erb + pvi_int,
           data = df_exp %>% filter(accuracy == "1", task == "NLS"),
           family = binomial(link = "logit"))

#For only accurate productions from PEPS-C:
glmer(stress_pattern ~ pvi_dur + pvi_f0_erb + pvi_int + (1 | ID),
           data = df_exp %>% filter(accuracy == "1", task == "PEPSC"),
           family = binomial(link = "logit"))


#due to singularity, run without random effect structure
glm(stress_pattern ~ pvi_dur + pvi_f0_erb + pvi_int,
           data = df_exp %>% filter(accuracy == "1", task == "PEPSC"),
           family = binomial(link = "logit"))
```

Model Summaries:

#NLS MODEL:

```
Generalized linear mixed model fit by maximum likelihood (Laplace
Approximation) ['glmerMod']
Family: binomial  ( logit )
Formula: stress_pattern ~ pvi_dur + pvi_f0_erb + pvi_int + (1 | ID)
Data: df_exp %>% filter(accuracy == "1", task == "NLS")

   AIC       BIC    logLik    deviance   df.resid
   642.5    664.3   -316.2    632.5      569

Scaled residuals:
Min      1Q      Median   3Q      Max
-2.8831  -0.7390 -0.1918  0.6853  5.1375

Random effects:
Groups Name        Variance Std.Dev.
ID     (Intercept) 0        0
Number of obs: 574, groups:  ID, 55

Fixed effects:
             Estimate   Std. Error z value  Pr(>|z|)
(Intercept) -1.235677   0.182424   -6.774   1.26e-11 ***
pvi_dur     -0.017614   0.001983    -8.884  < 2e-16 ***
pvi_f0_erb   0.003170   0.005546   0.572    0.568
pvi_int     -0.067883   0.014499   -4.682   2.84e-06 ***
---
Signif. codes:  0 '***' 0.001 '**' 0.01 '*' 0.05 '.' 0.1 ' ' 1

Correlation of Fixed Effects:
           (Intr) pvi_dr pv_f0_
pvi_dur     0.834
pvi_f0_erb  0.096 -0.010
pvi_int    -0.219 -0.117 -0.083
optimizer (Nelder_Mead) convergence code: 0 (OK)
boundary (singular) fit: see help('isSingular')
```

#SIMPLIFIED NLS MODEL:

```
glm(formula = stress_pattern ~ pvi_dur + pvi_f0_erb + pvi_int,
      family = binomial(link = "logit"),
      data = df_exp %>% filter(accuracy == "1", task == "NLS"))

Coefficients:
             Estimate   Std. Error z value Pr(>|z|)
(Intercept) -1.235677   0.182424   -6.774  1.26e-11 ***
pvi_dur     -0.017614   0.001983   -8.883  < 2e-16 ***
pvi_f0_erb   0.003170   0.005546    0.572  0.568
pvi_int     -0.067883   0.014499   -4.682  2.84e-06 ***
---
Signif. codes:  0 '***' 0.001 '**' 0.01 '*' 0.05 '.' 0.1 ' ' 1
```

```
(Dispersion parameter for binomial family taken to be 1)
Null deviance: 795.71  on 573  degrees of freedom
Residual deviance: 632.49  on 570  degrees of freedom
(26 observations deleted due to missingness)
AIC: 640.49
Number of Fisher Scoring iterations: 4
```

#PEPS-C model:

```
Generalized linear mixed model fit by maximum likelihood (Laplace
Approximation) ['glmerMod']
Family: binomial  ( logit )
Formula: stress_pattern ~ pvi_dur + pvi_f0_erb + pvi_int + (1 | ID)
Data: df_exp %>% filter(accuracy == "1", task == "PEPSC")

AIC      BIC     logLik    deviance   df.resid
401.6    422.9   -195.8    391.6      512

Scaled residuals:
Min        1Q      Median     3Q      Max
-3.4586   -0.3671  0.0626    0.4154  6.1510

Random effects:
 Groups Name        Variance Std.Dev.
 ID     (Intercept) 0.3565   0.5971
Number of obs: 517, groups:  ID, 50

Fixed effects:
             Estimate Std. Error z value Pr(>|z|)
(Intercept) -0.011626   0.254786  -0.046   0.9636
pvi_dur     -0.007241   0.002952  -2.453   0.0142 *
pvi_f0_erb  -0.010426   0.006450  -1.616   0.1060
pvi_int     -0.229590   0.023895  -9.608   <2e-16 ***
---
Signif. codes:  0 '***' 0.001 '**' 0.01 '*' 0.05 '.' 0.1 ' ' 1

Correlation of Fixed Effects:
           (Intr) pvi_dr pv_f0_
pvi_dur     0.762
pvi_f0_erb -0.132 -0.015
pvi_int    -0.127 -0.001 -0.042
```

#SIMPLIFIED PEPS-C MODEL:

```
Call:
glm(formula = stress_pattern ~ pvi_dur + pvi_f0_erb + pvi_int,
      family = binomial(link = "logit"),
      data = df_exp %>% filter(accuracy == "1", task == "PEPSC"))

Coefficients:
             Estimate Std. Error z value Pr(>|z|)
(Intercept) -0.065521   0.222362  -0.295  0.76825
pvi_dur     -0.007461   0.002756  -2.707  0.00679 **
pvi_f0_erb  -0.010396   0.006091  -1.707  0.08788 .
pvi_int     -0.209744   0.019163 -10.945  < 2e-16 ***
```

```
---
Signif. codes:  0 '***' 0.001 '**' 0.01 '*' 0.05 '.' 0.1 ' ' 1

(Dispersion parameter for binomial family taken to be 1)

    Null deviance: 716.28  on 516  degrees of freedom
Residual deviance: 394.31  on 513  degrees of freedom
  (106 observations deleted due to missingness)
AIC: 402.31

Number of Fisher Scoring iterations: 6
```

3. R code and statistical models that predict PVI measures by both sentence position and stress pattern (in Aim 3 of Statistical Analyses section): Three linear mixed effect models were run with an additional fixed factor included for sentence position (medial, final). This was alongside a fixed factor for stress pattern (trochaic, iambic) and a random intercept for participant ID. These models highlight differences in the production of lexical stress pattern (trochaic vs. iambic) due to sentence position. Results show that sentence position was a mediating factor on duration and f0 measures, but not intensity.

R Code:

```
m.dur.pos <- lmer(pvi_dur ~ position * stress_pattern + (1 | ID),
      data = df_exp_posfilter)

m.f0.pos <- lmer(pvi_f0_erb ~ position * stress_pattern + (1 | ID),
      data = df_exp_posfilter)

m.int.pos <- lmer(pvi_int ~ position * stress_pattern + (1 | ID),
      data = df_exp_posfilter)
```

Model Summaries:

```
#summary(m.dur.pos)
Linear mixed model fit by REML. t-tests use Satterthwaite's method
['lmerModLmerTest']
Formula: pvi_dur ~ position * stress_pattern + (1 | ID)Data:
df_exp_posfilter

REML criterion at convergence: 6294.6

Scaled residuals:
Min      1Q      Median   3Q      Max
-2.7002  -0.7195 -0.1160  0.5985  3.3910

Random effects:
 Groups   Name        Variance Std.Dev.
 ID       (Intercept) 159.7    12.64
Residual             2272.8   47.67
Number of obs: 595, groups:  ID, 55

Fixed effects:
```

```
                          Estimate   Std.Err df       t value  Pr(>|t|)
(Intercept)               -83.957    5.491   451.890  -15.291  < 2e-16 ***
positionM                 50.712     6.237   578.183   8.131   2.61e-15 ***
stress_pattern2           -39.016    7.770   561.789  -5.021   6.91e-07 ***
positionM:stress_pattern2-24.899    9.057   568.867  -2.749   0.00616 **
---
Signif. codes:  0 '***' 0.001 '**' 0.01 '*' 0.05 '.' 0.1 ' '
Correlation of Fixed Effects:
            (Intr) postnM strs_2
positionM   -0.805
strss_pttr2 -0.635  0.564
pstnM:str_2  0.549 -0.679 -0.864
```

#summary(m.f0.pos)

```
Linear mixed model fit by REML. t-tests use Satterthwaite's method
['lmerModLmerTest']
Formula: pvi_f0_erb ~ position * stress_pattern + (1 | ID)
Data: df_exp_posfilter

REML criterion at convergence: 4857.8

Scaled residuals:
Min      1Q       Median   3Q       Max
-5.0049  -0.3665  0.0388  0.3672  3.7172

Random effects:
 Groups   Name        Variance Std.Dev.
 ID       (Intercept)  22.32    4.725
 Residual             290.21   17.036
Number of obs: 569, groups:  ID, 55

Fixed effects:
                          Estimate Std. Error      df t value Pr(>|t|)
(Intercept)                 -8.394      2.039 436.177  -4.118 4.58e-05 ***
positionM                    7.033      2.296 549.824   3.063   0.0023 **
stress_pattern2              5.308      2.881 531.391   1.842   0.0660 .
positionM:stress_pattern2   -7.363      3.338 536.839  -2.206   0.0278 *
---
Signif. codes:  0 '***' 0.001 '**' 0.01 '*' 0.05 '.' 0.1 ' ' 1

Correlation of Fixed Effects:
            (Intr) postnM strs_2
positionM   -0.810
strss_pttr2 -0.634  0.567
pstnM:str_2  0.550 -0.676 -0.868
```

#summary(m.int.pos)

```
Linear mixed model fit by REML. t-tests use Satterthwaite's method
['lmerModLmerTest']
Formula: pvi_int ~ position * stress_pattern + (1 | ID)
```

```
Data: df_exp_posfilter

REML criterion at convergence: 4182.2

Scaled residuals:
Min      1Q       Median   3Q      Max
-5.1819  -0.5063  0.0344   0.5836  4.1853


Random effects:
 Groups   Name        Variance Std.Dev.
 ID       (Intercept)  0.06196 0.2489
 Residual             67.80732 8.2345
Number of obs: 594, groups:  ID, 55

Fixed effects:
                          Estimate Std. Error      df t value Pr(>|t|)
(Intercept)                  4.661      0.894 533.935   5.214 2.65e-07 ***
positionM                   -1.554      1.058 589.984  -1.469 0.142466
stress_pattern2             -4.964      1.329 575.400  -3.734 0.000207 ***
positionM:stress_pattern2   -0.899      1.545 583.344  -0.582 0.560817
---
Signif. codes:  0 '***' 0.001 '**' 0.01 '*' 0.05 '.' 0.1 ' ' 1

Correlation of Fixed Effects:
            (Intr) postnM strs_2
positionM   -0.844
strss_pttr2 -0.671  0.567
pstnM:str_2  0.578 -0.685 -0.861
```

Sensitivity (post-hoc) Power Analysis for Developmental Comparisons

All effects two-tailed, α = 0.05

1. Developmental group comparisons (expressive and receptive tasks)

a. Child subgroup comparisons (n = 15 → younger: n = 7 vs. older: n = 8)
SE for standardized mean difference = Sqrt(1/7 + 1/8) = 0.518
Minimum detectable effect (MDE) (80% power): d ≈ 2.80 * 0.518 = 1.45
Power for smaller effects (illustrative): d = 0.50 → ~30% power; d = 0.80 → 55-60% power
Interpretation: Within-child comparisons are underpowered; only very large effects (d ≈ 1.45) would be detectable reliably.

b. Adult (n = 40) vs. all children (n = 15)
SE = Sqrt(1/40 + 1/15) = 0.303
MDE (80% power): d ≈ 2.80 * 0.303 = 0.85 (≈0.98 for 90% power)
Interpretation: Adult-child contrasts have moderate sensitivity; small effects (d ≈ 0.3-0.5) are underpowered

2. Correlations (criterion validity within children, n = 15)
Using Fisher z: required |z| ≈ (1.96+0.84)/sqrt(15-3) = 0.81 → |r| ≈ 0.67
Approximate mapping to Kendall's tau (ties ignored): |τ| ≈ 0.47
Interpretation: With many tied (ceiling) scores, effective power is even lower; null/weak correlations are expected under restricted range

Acoustic models (expressive)
Despite many tokens, effective power is governed by the number of participants/items under the mixed-effects structure. The robust main effects for PVI_duration and PVI_intensity indicate adequate sensitivity to large cue effects; task*group interactions within children should be treated as exploratory given n = 7/8.
