# Supplementary material for: Understanding development of Mainstream US English lexical stress using semi-naturalistic stimuli
Source: PLoS One. 2026 Apr 29;21(4):e0345745. doi: 10.1371/journal.pone.0345745 (PMC13128110; doi:10.1371/journal.pone.0345745)
Supplement: S3 Files — (ZIP) [file pone.0345745.s001.zip › S3_NLSTaskFiles/NLS-ReceptiveTaskWithSounds.pptx]

## Slide 1
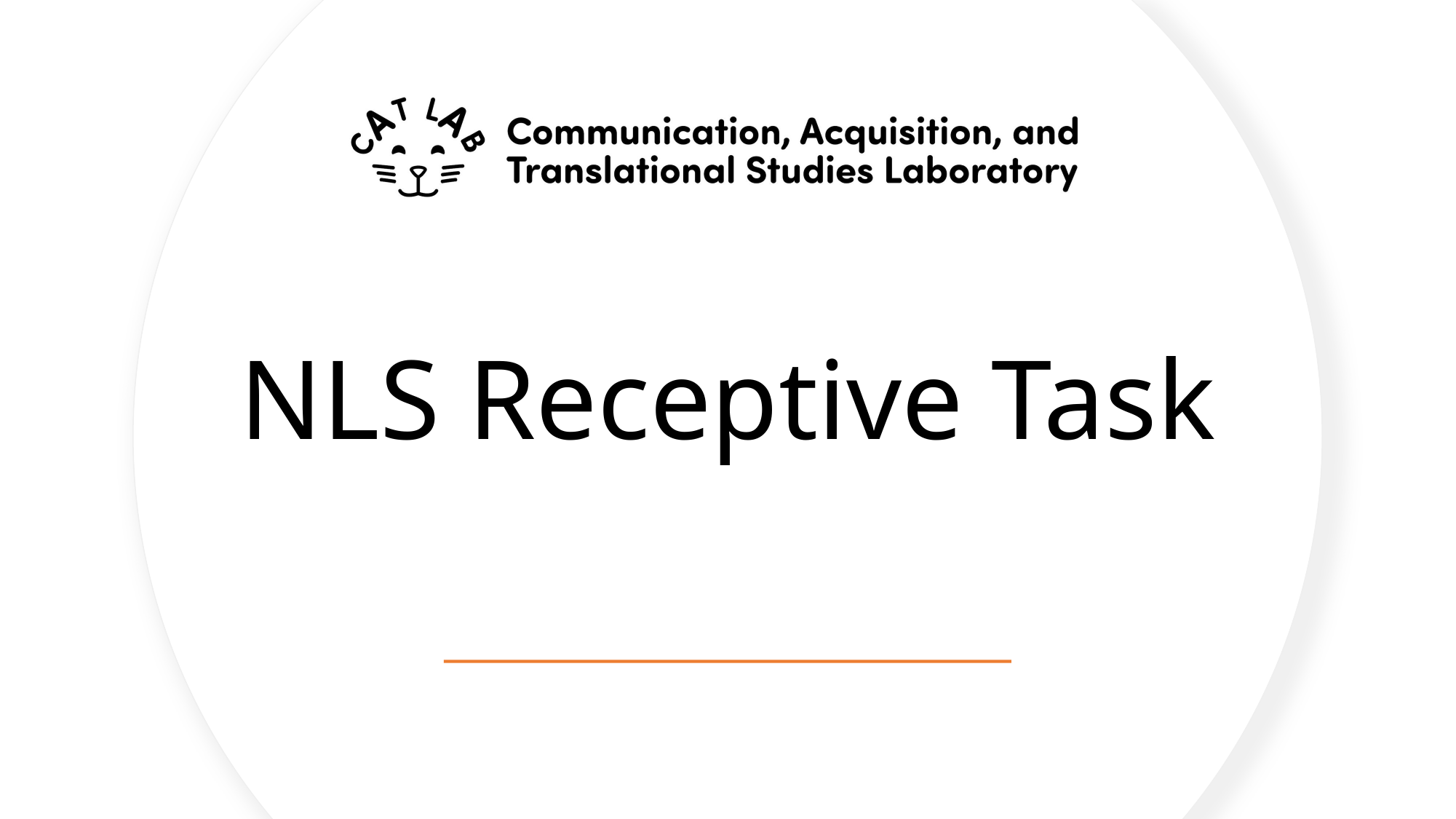

# NLS Receptive Task

## Slide 2
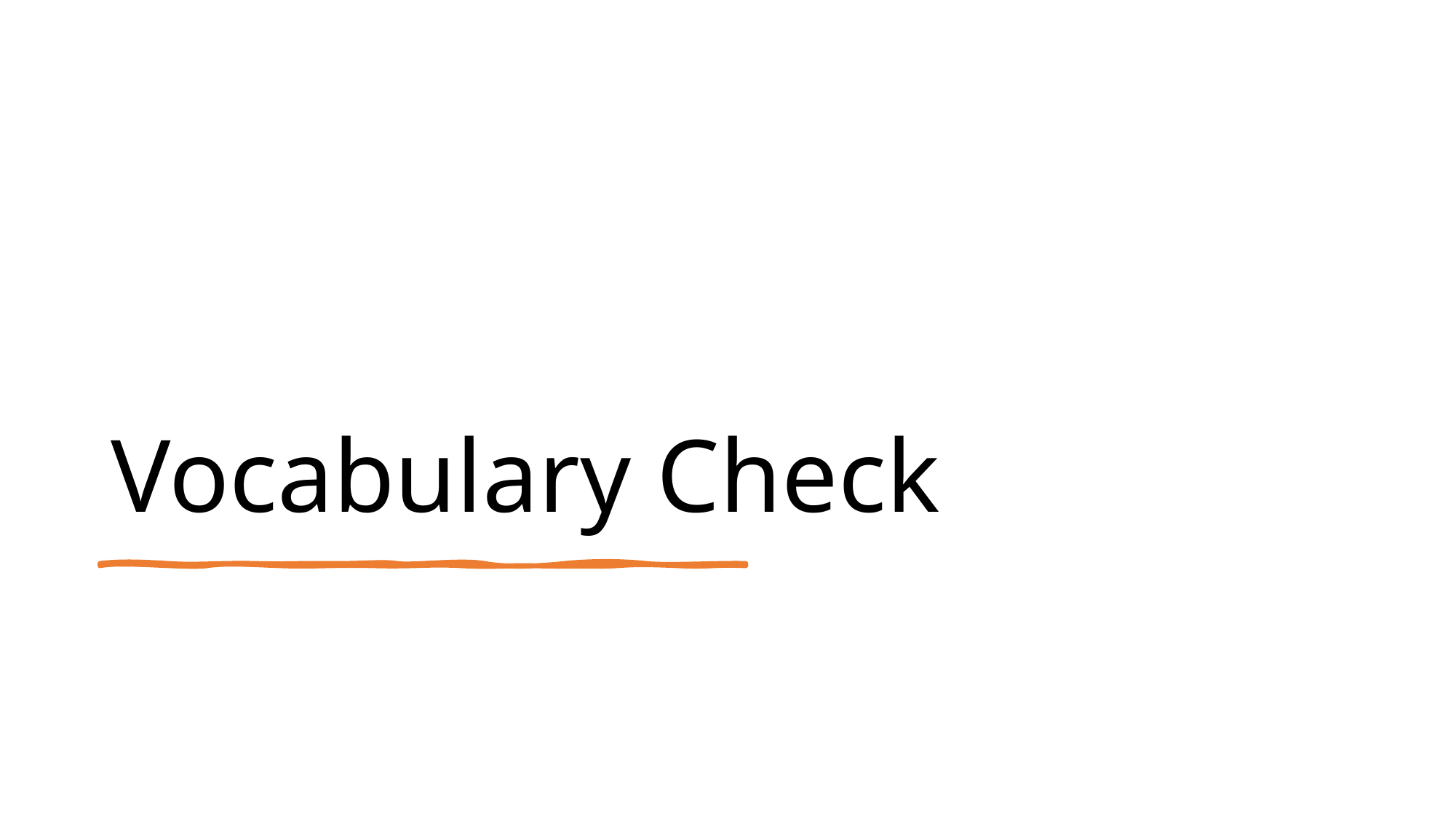

# Vocabulary Check

## Slide 3
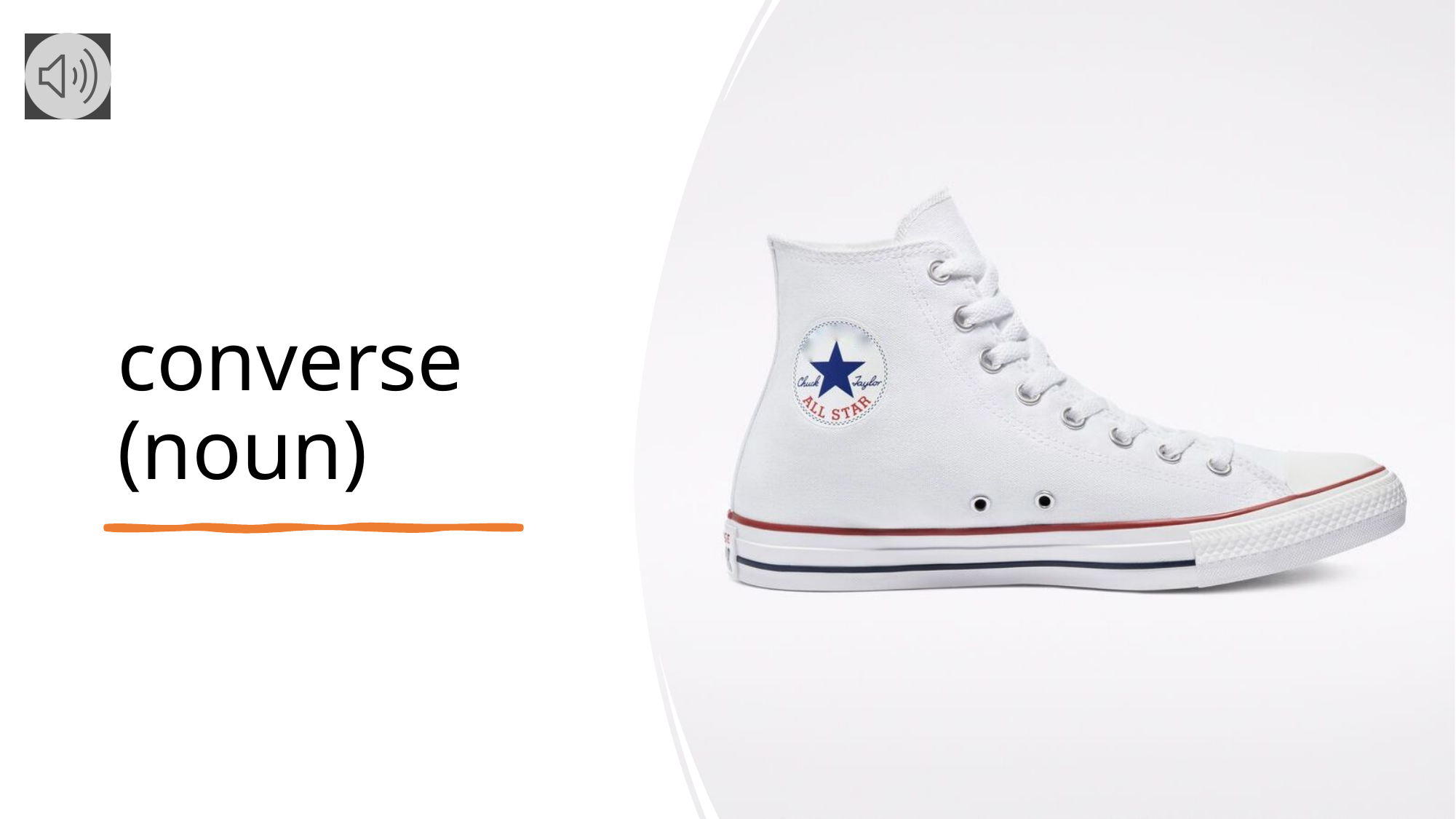

# converse (noun)

## Slide 4
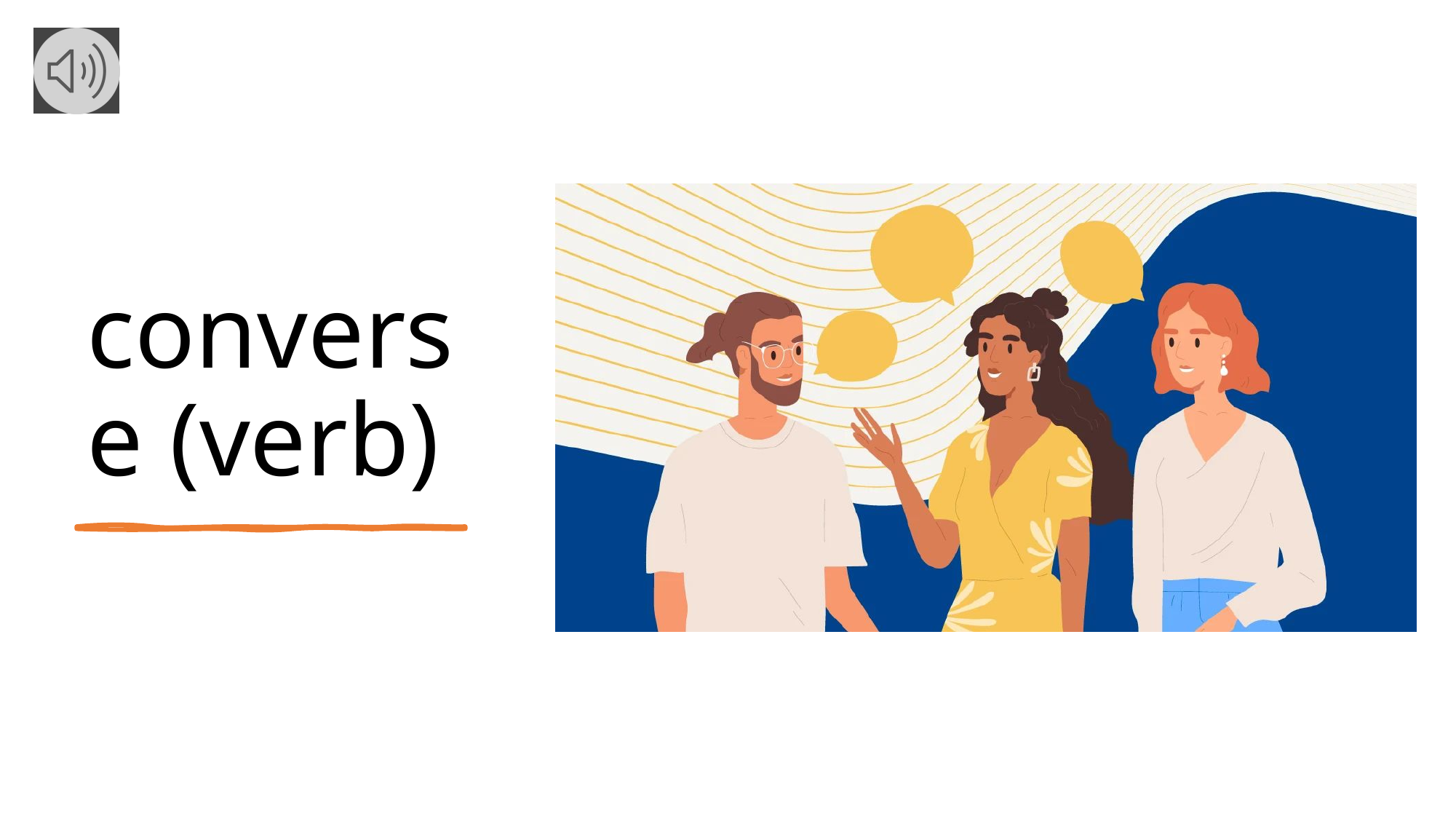

# converse (verb)

## Slide 5
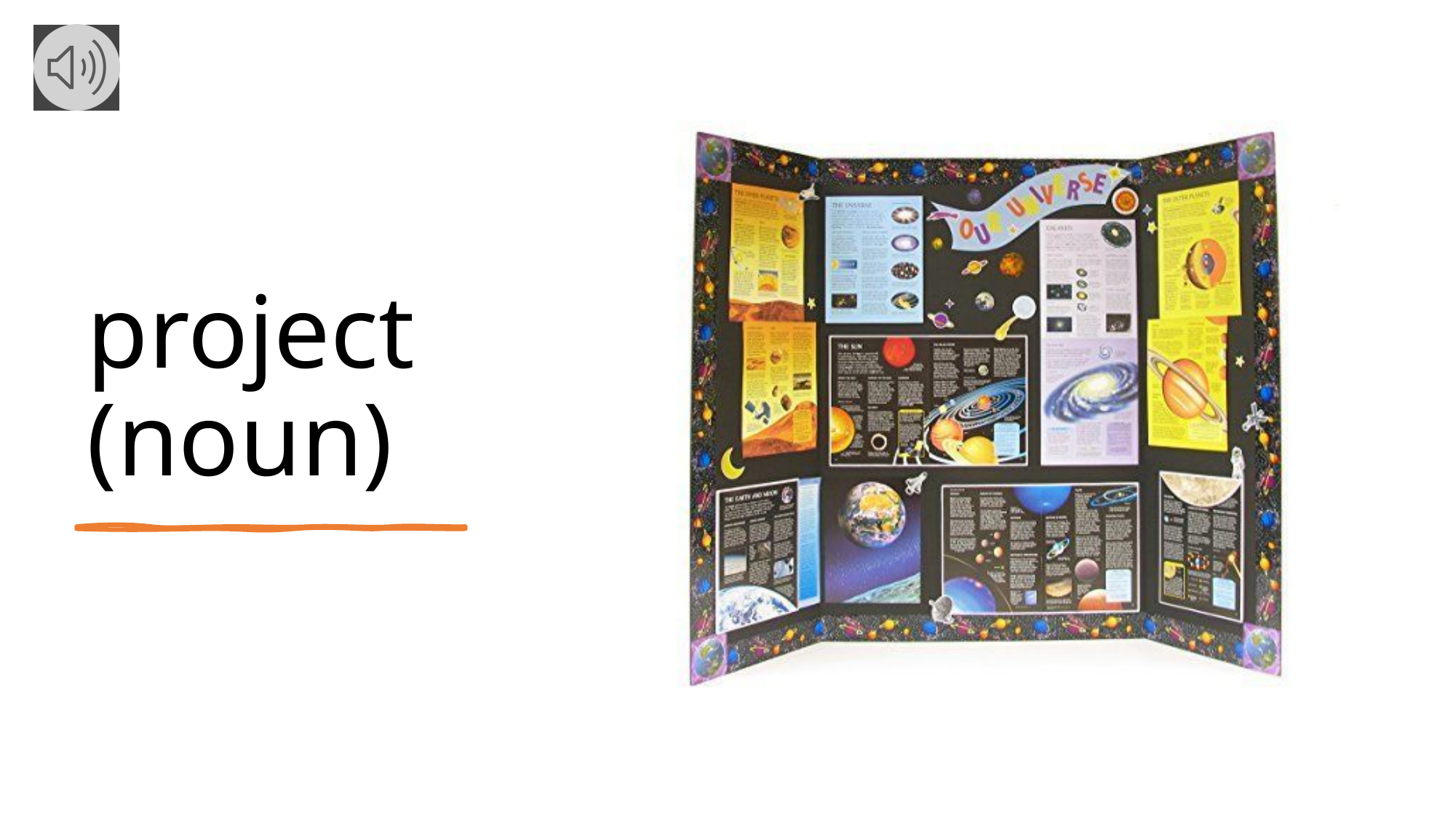

# project (noun)

## Slide 6
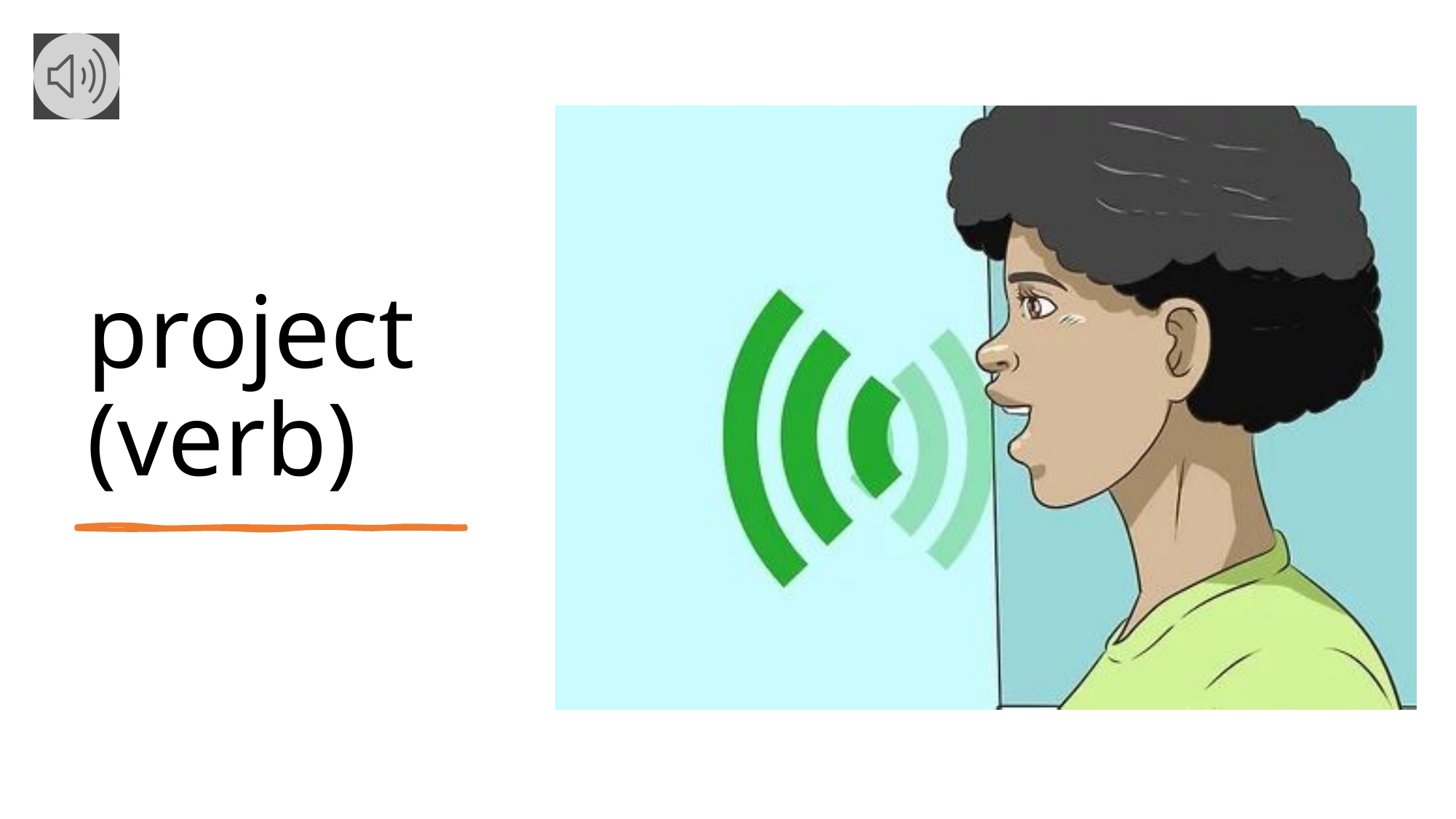

# project (verb)

## Slide 7
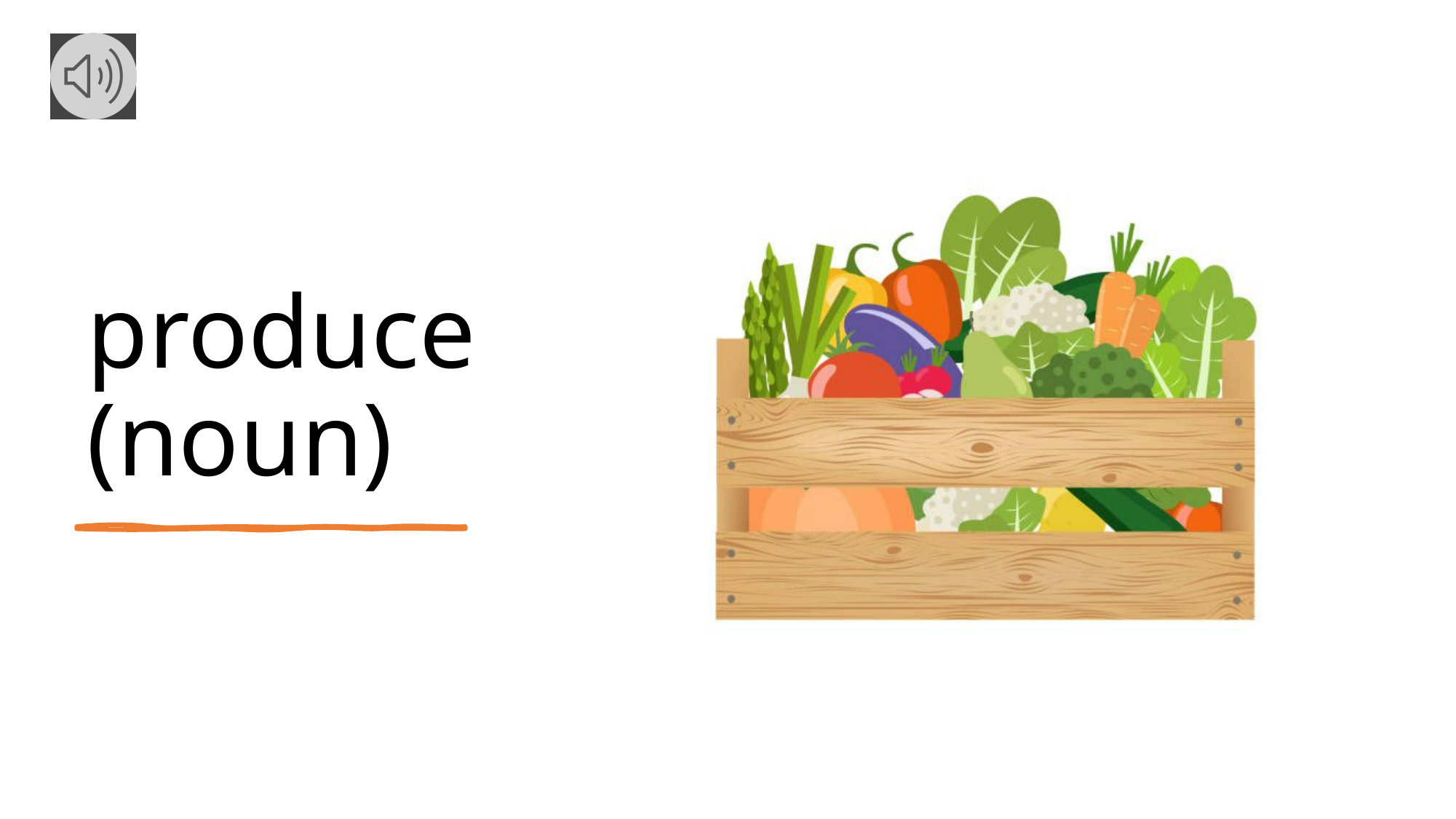

# produce (noun)

## Slide 8
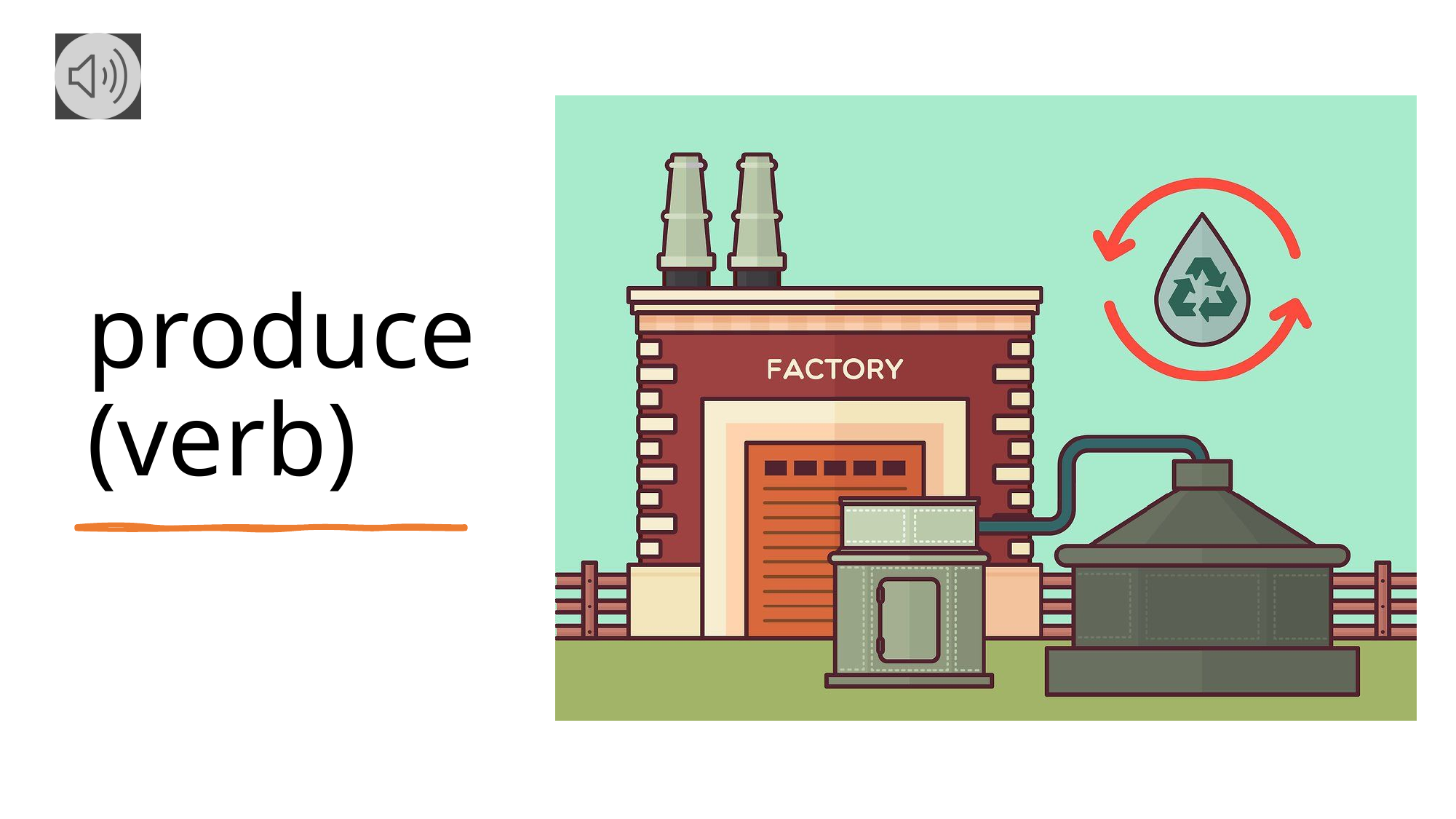

# produce (verb)

## Slide 9
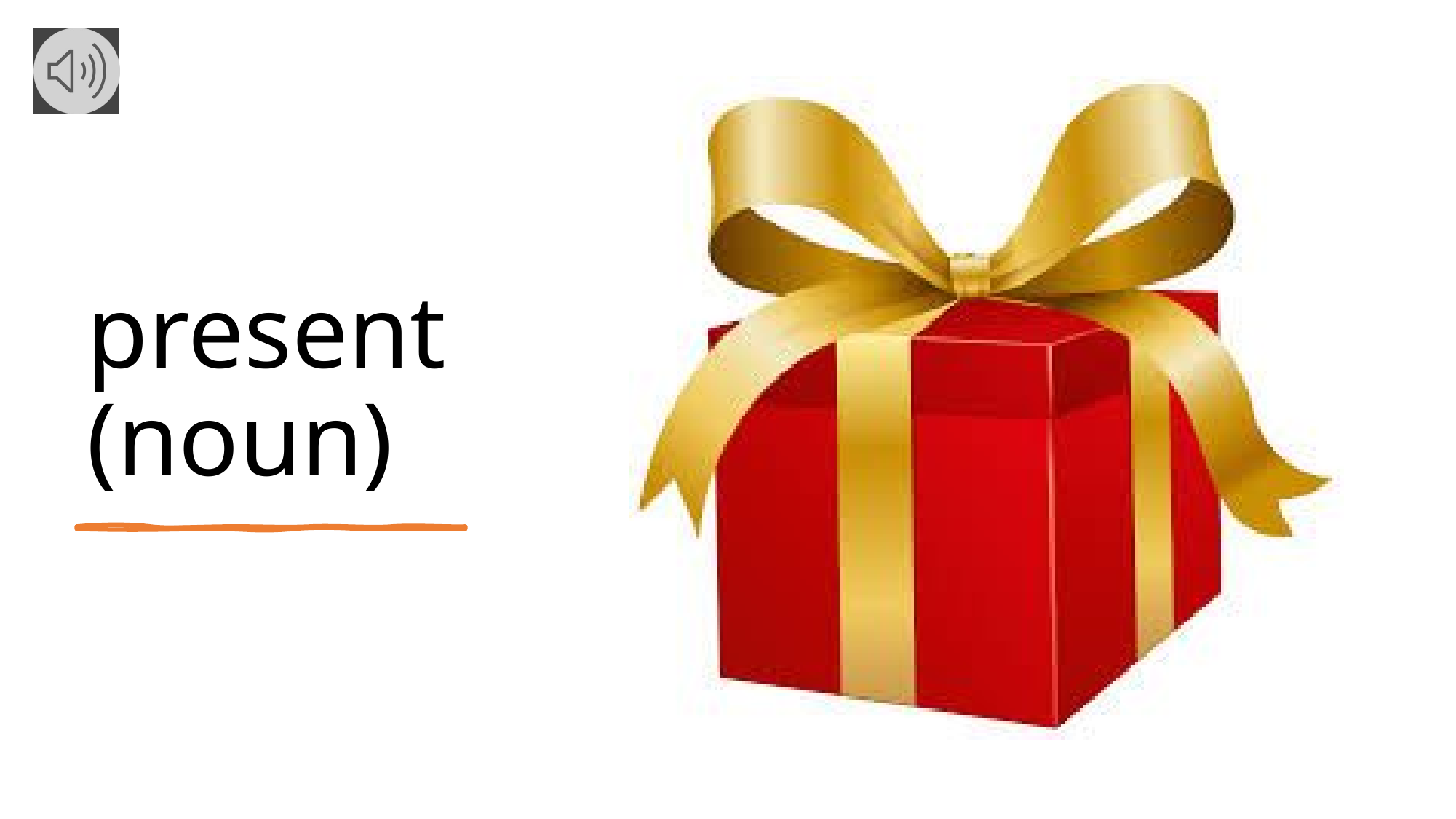

# present (noun)

## Slide 10
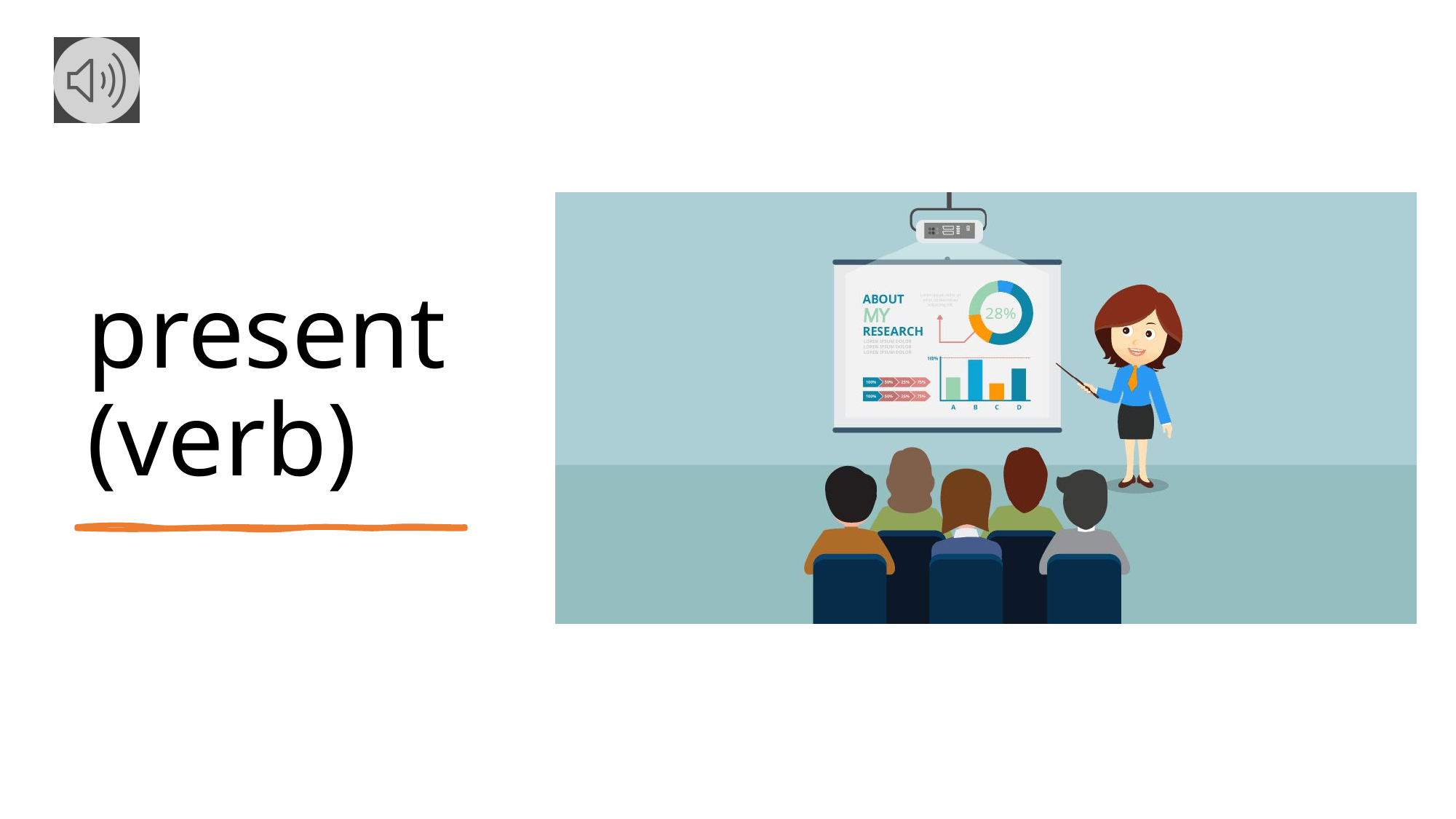

# present (verb)

## Slide 11
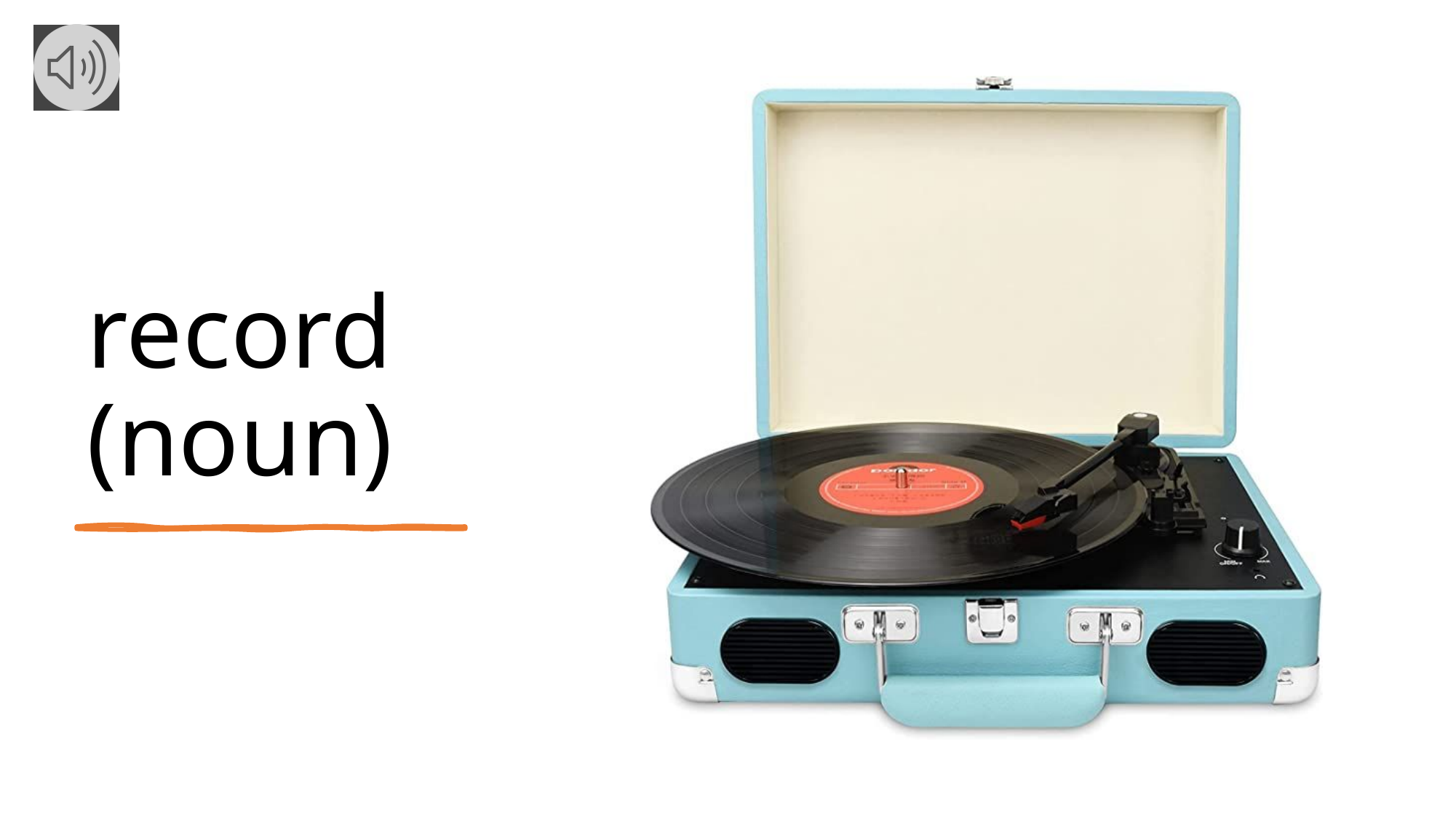

# record (noun)

## Slide 12
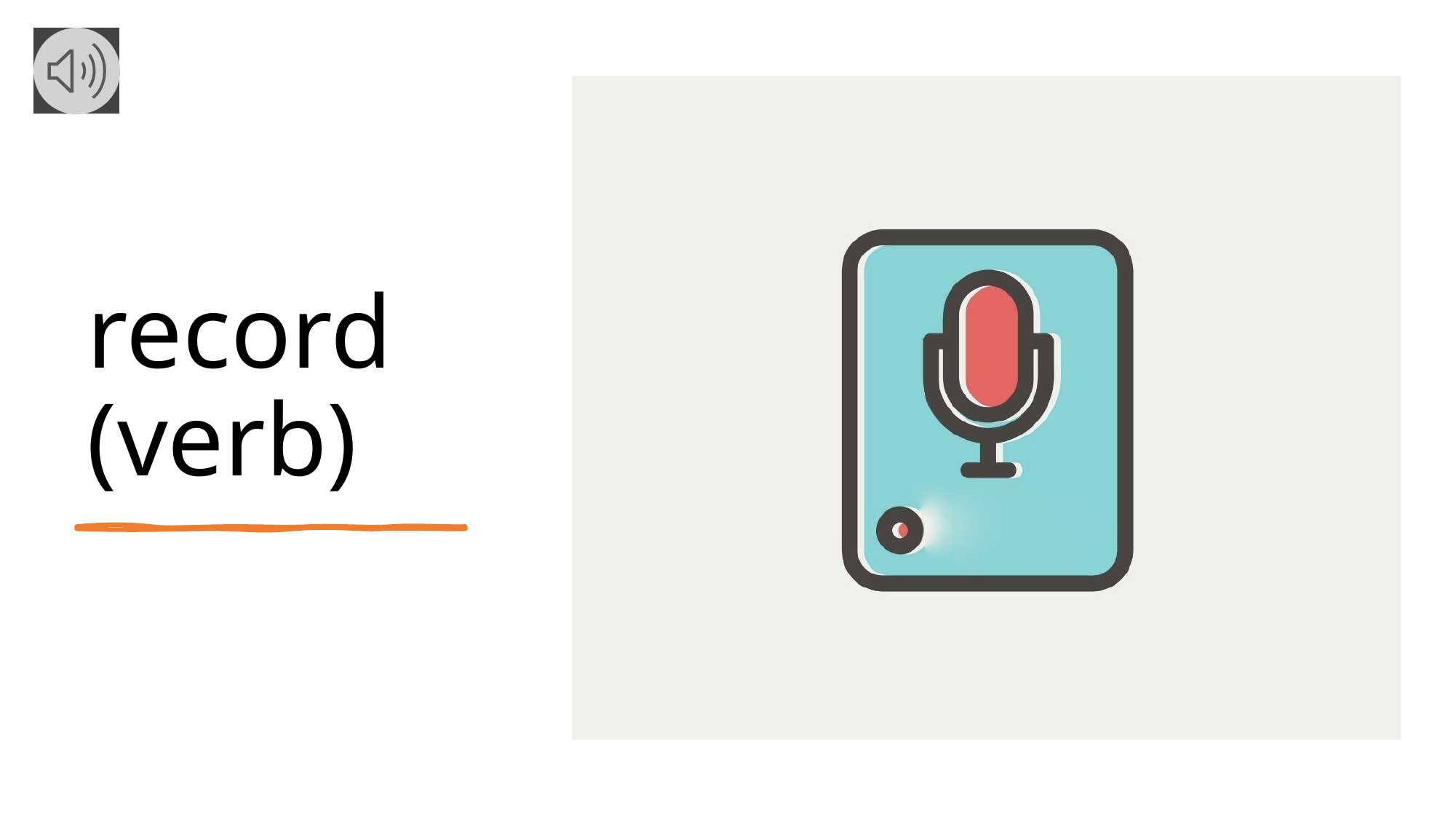

# record (verb)

## Slide 13
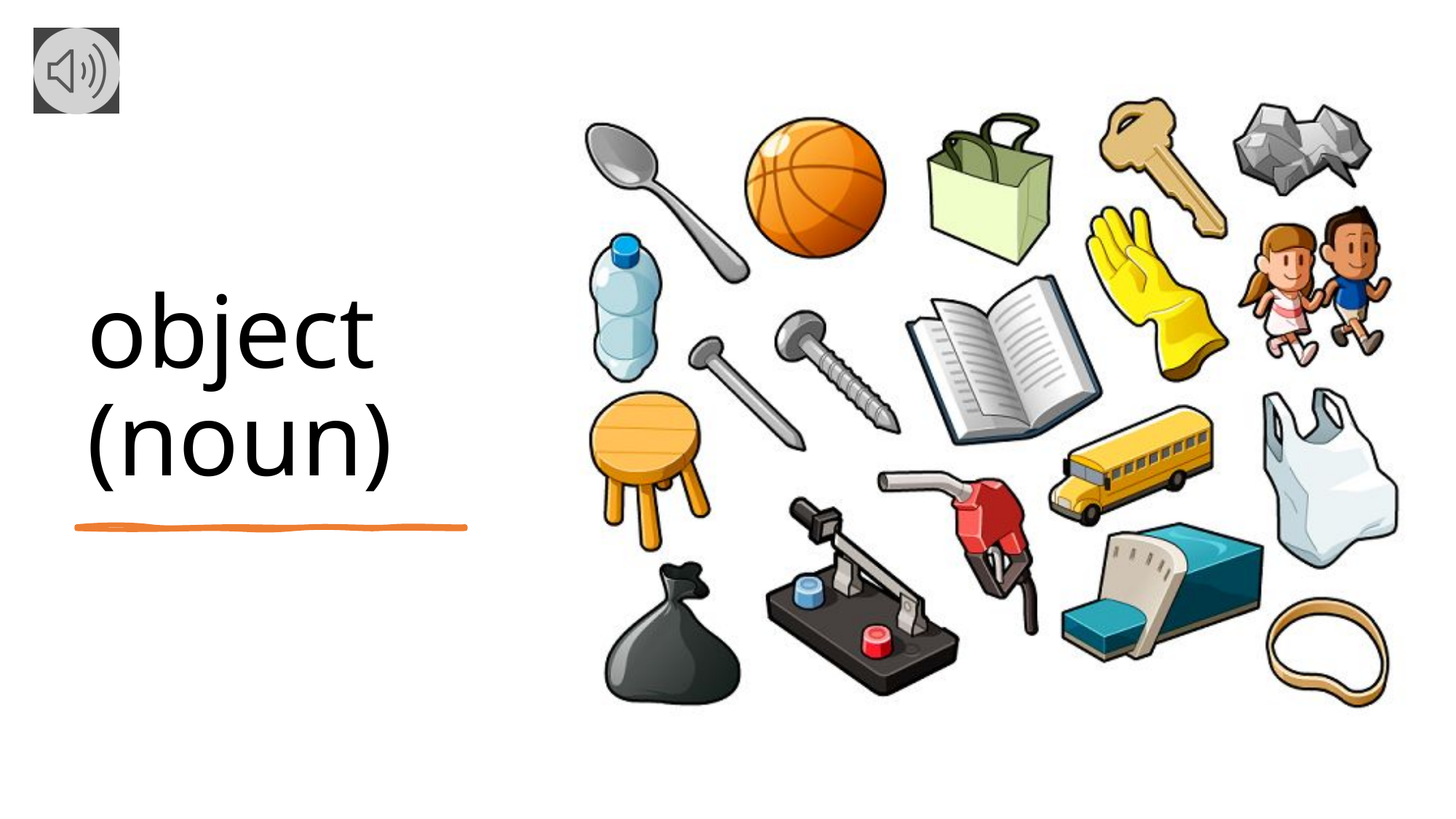

# object (noun)

## Slide 14
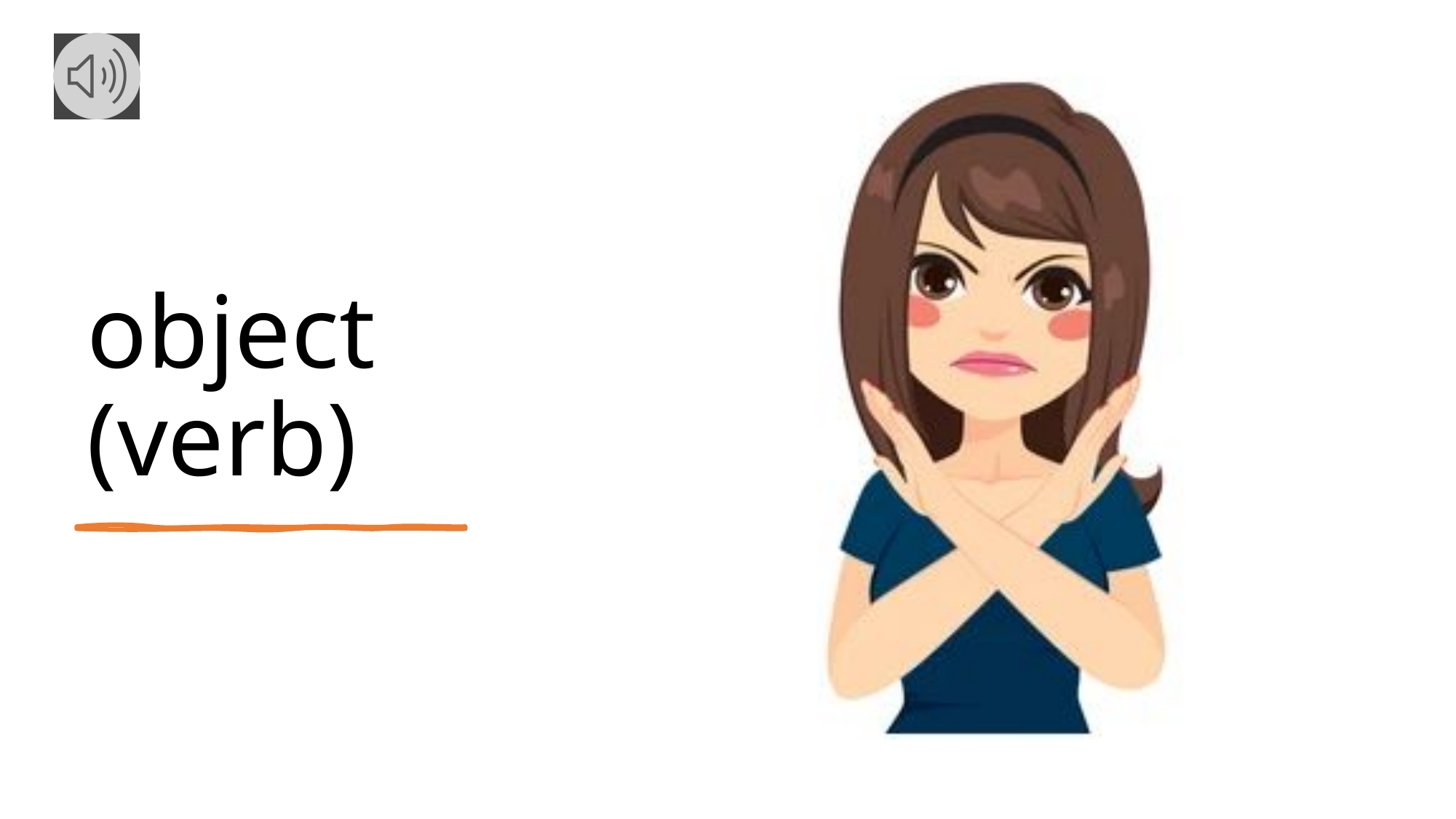

# object (verb)

## Slide 15
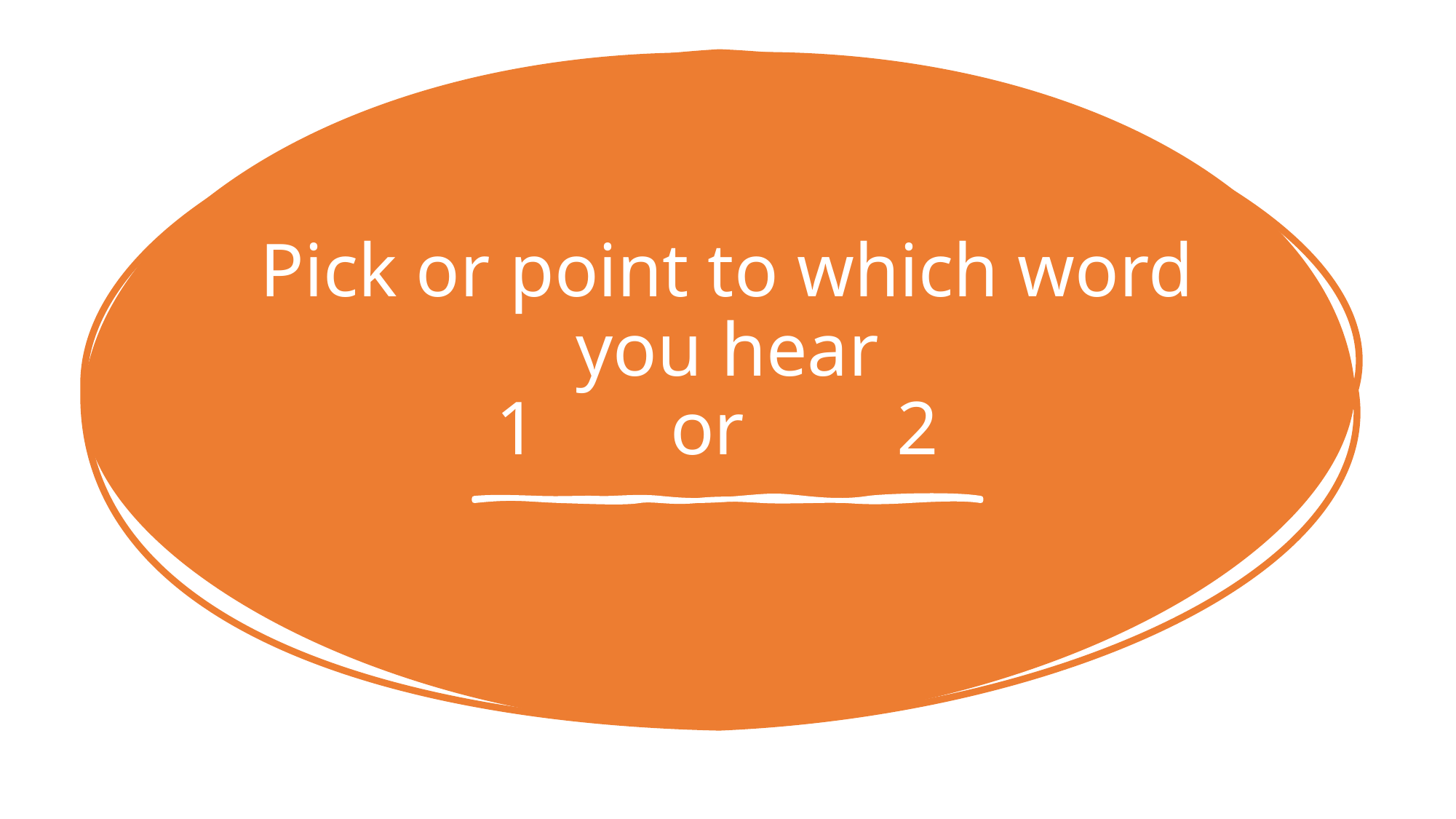

# Pick or point to which word you hear 1       or        2

## Slide 16
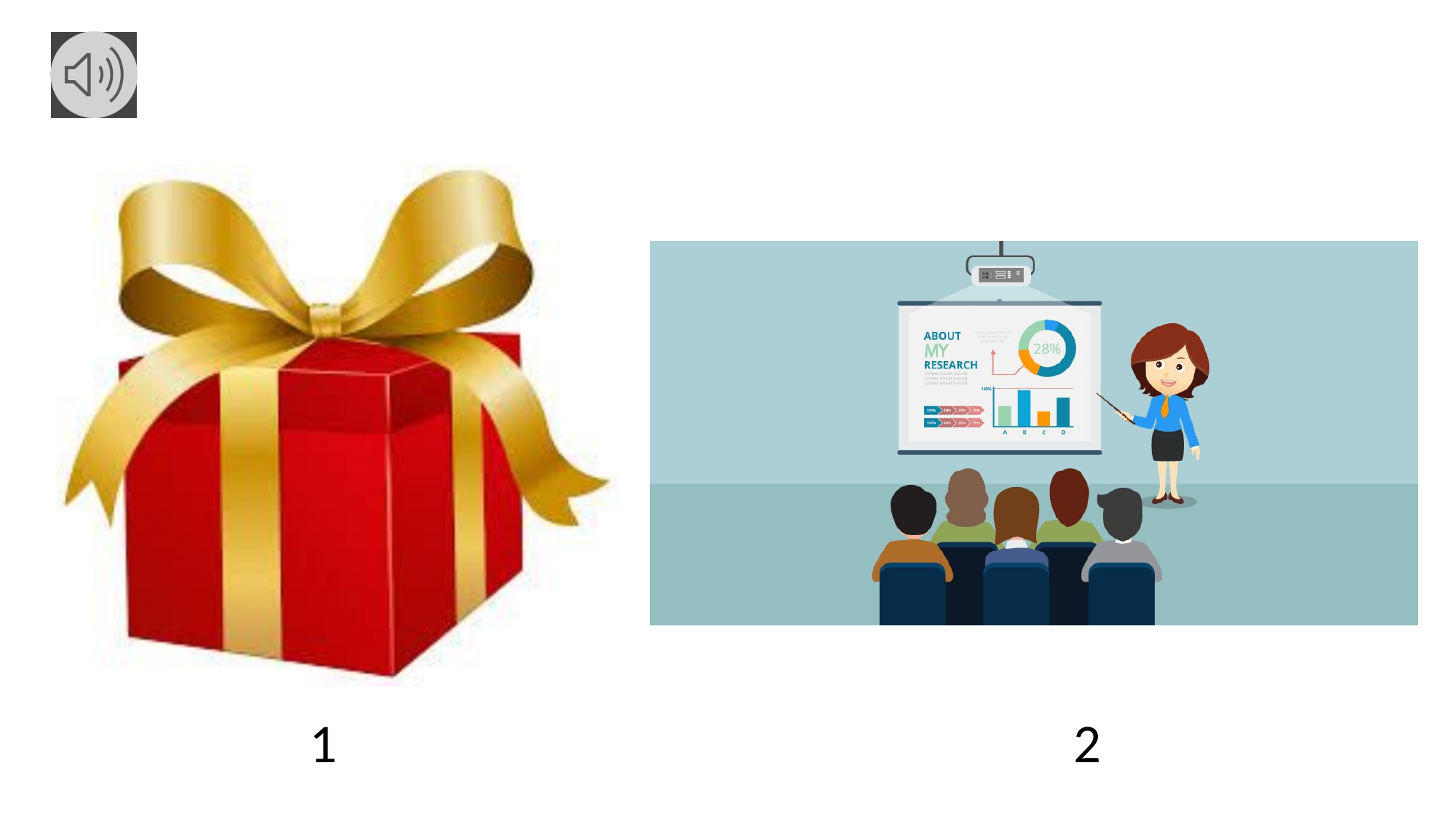

1
2

## Slide 17
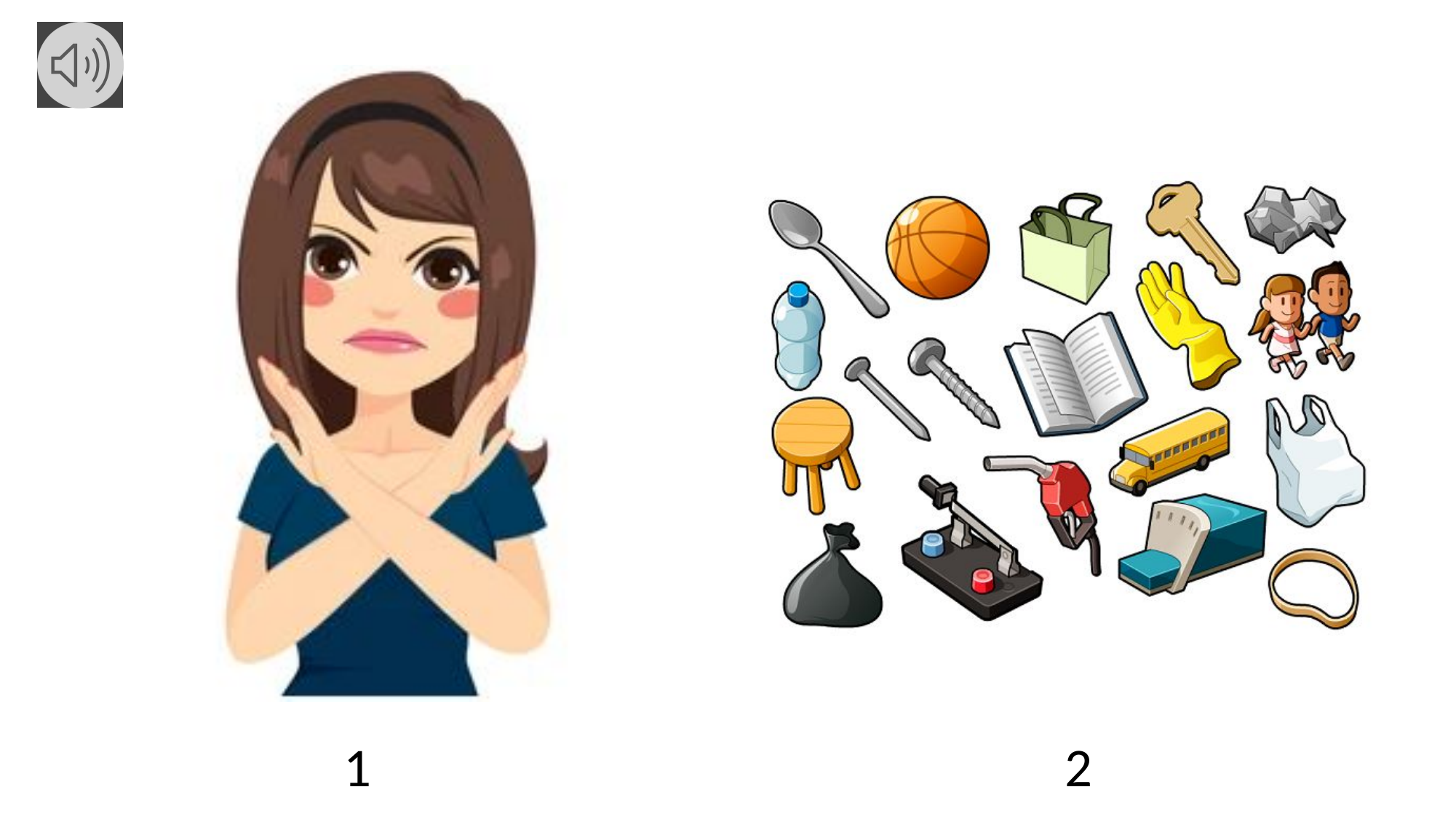

1
2

## Slide 18
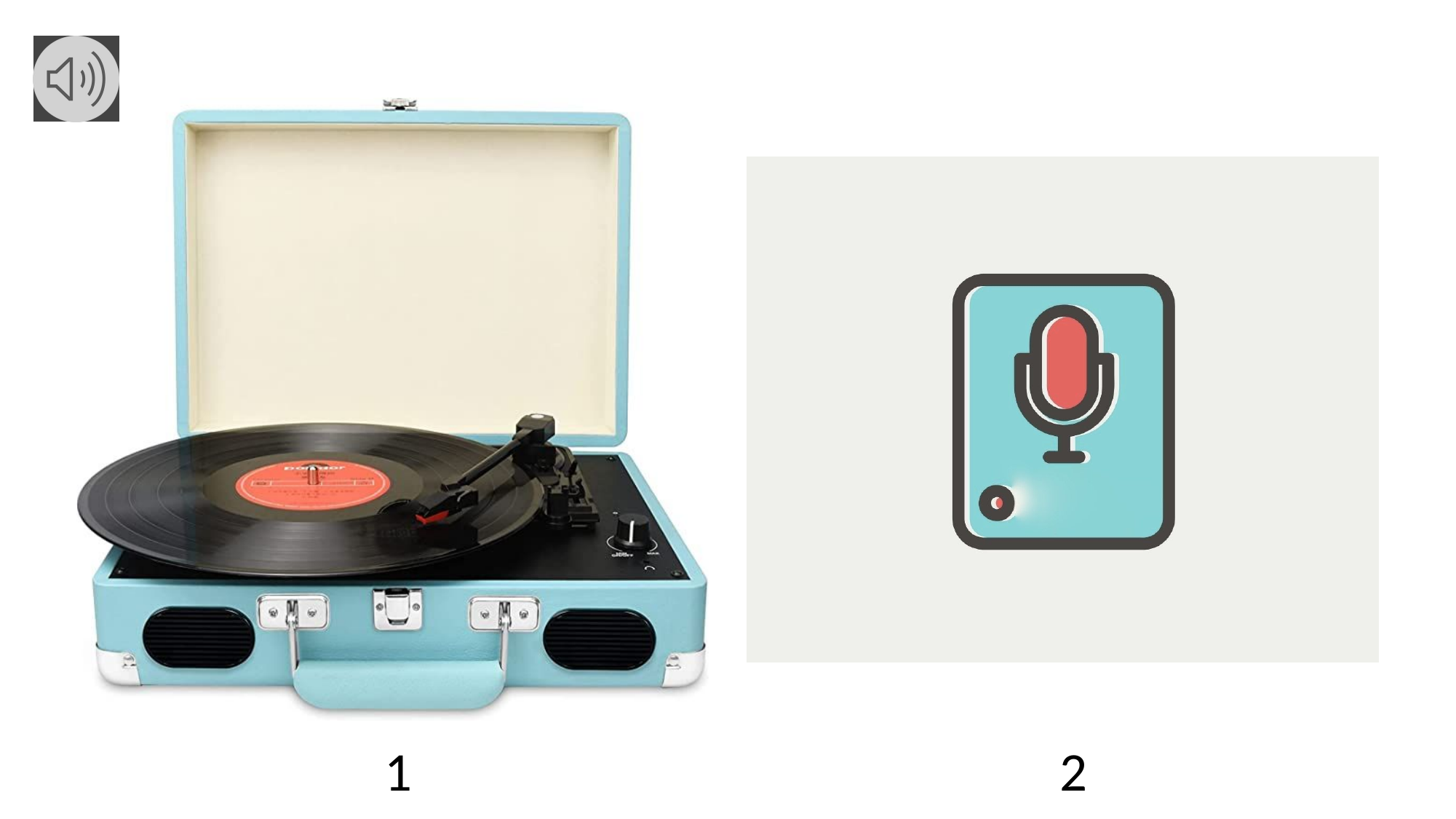

1
2

## Slide 19
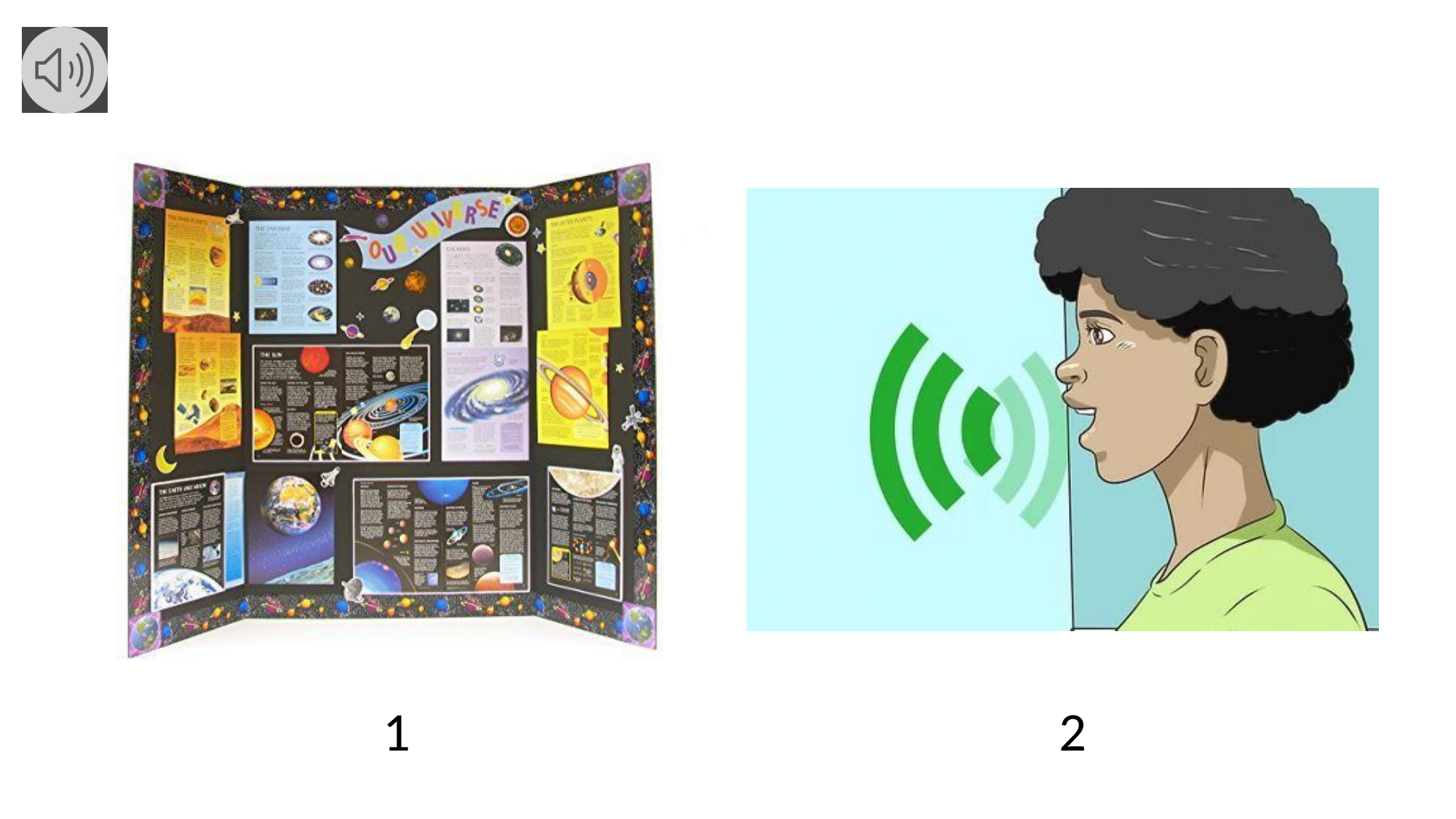

1
2

## Slide 20
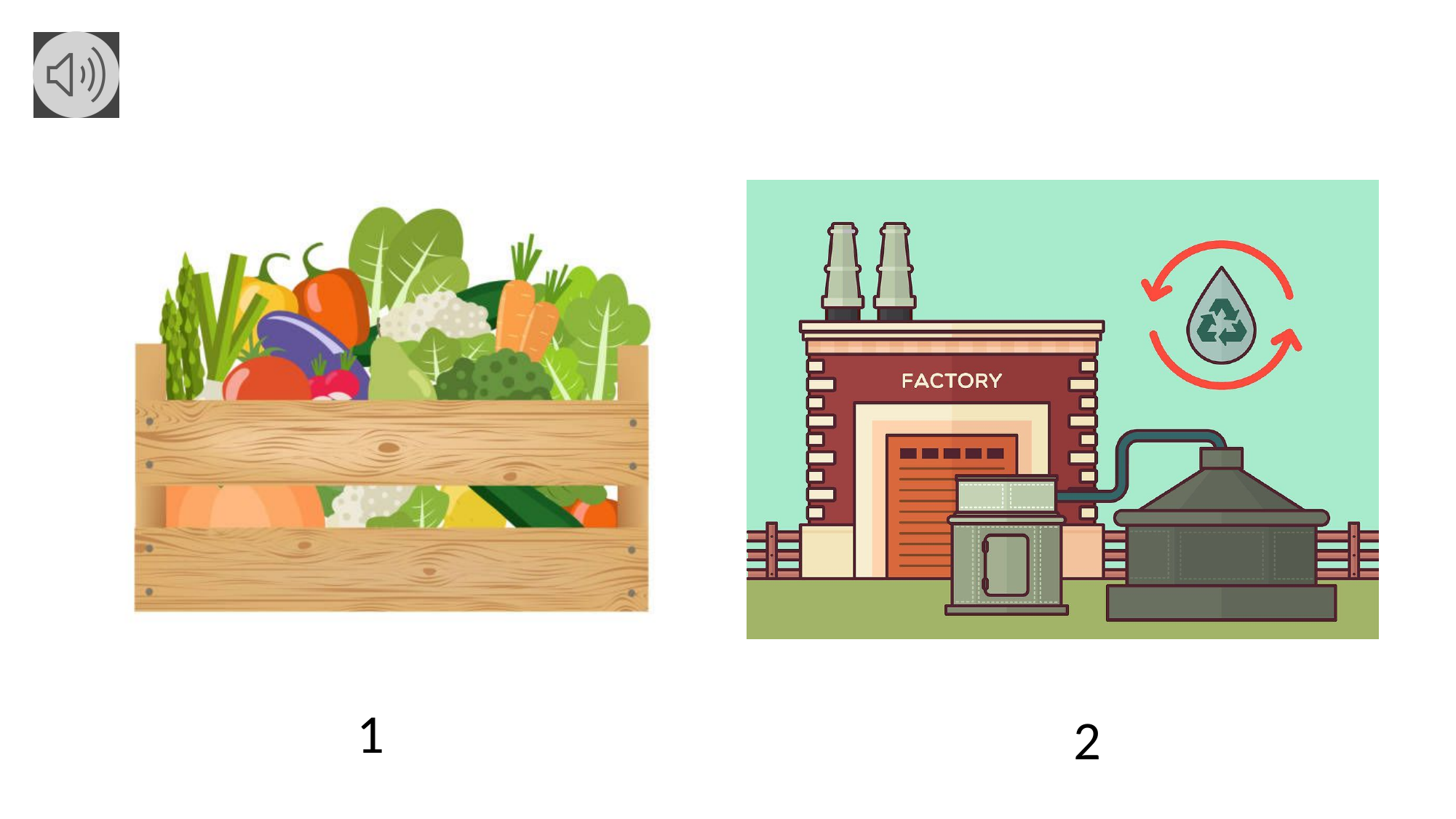

1
2

## Slide 21
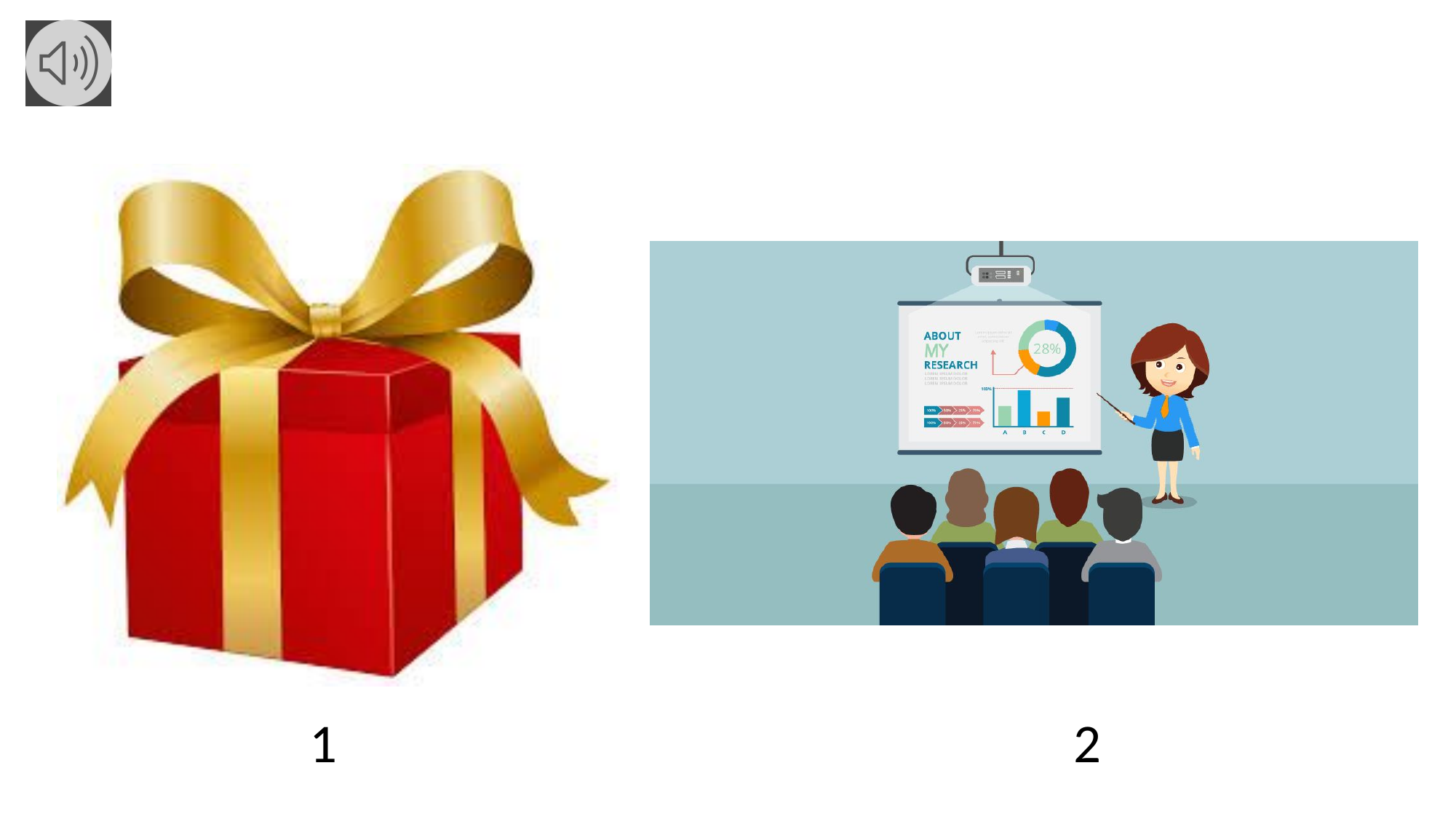

1
2

## Slide 22
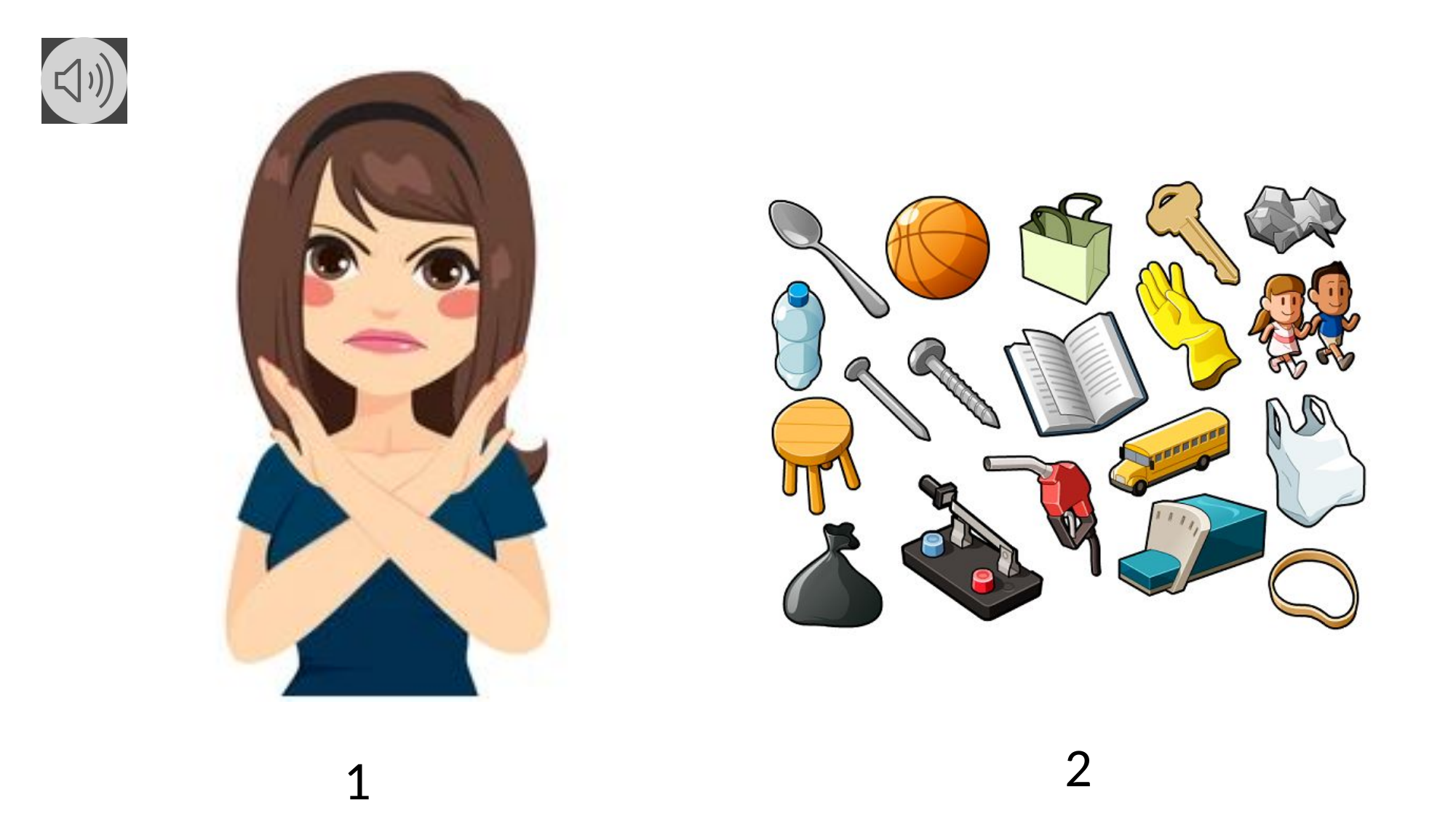

2
1

## Slide 23
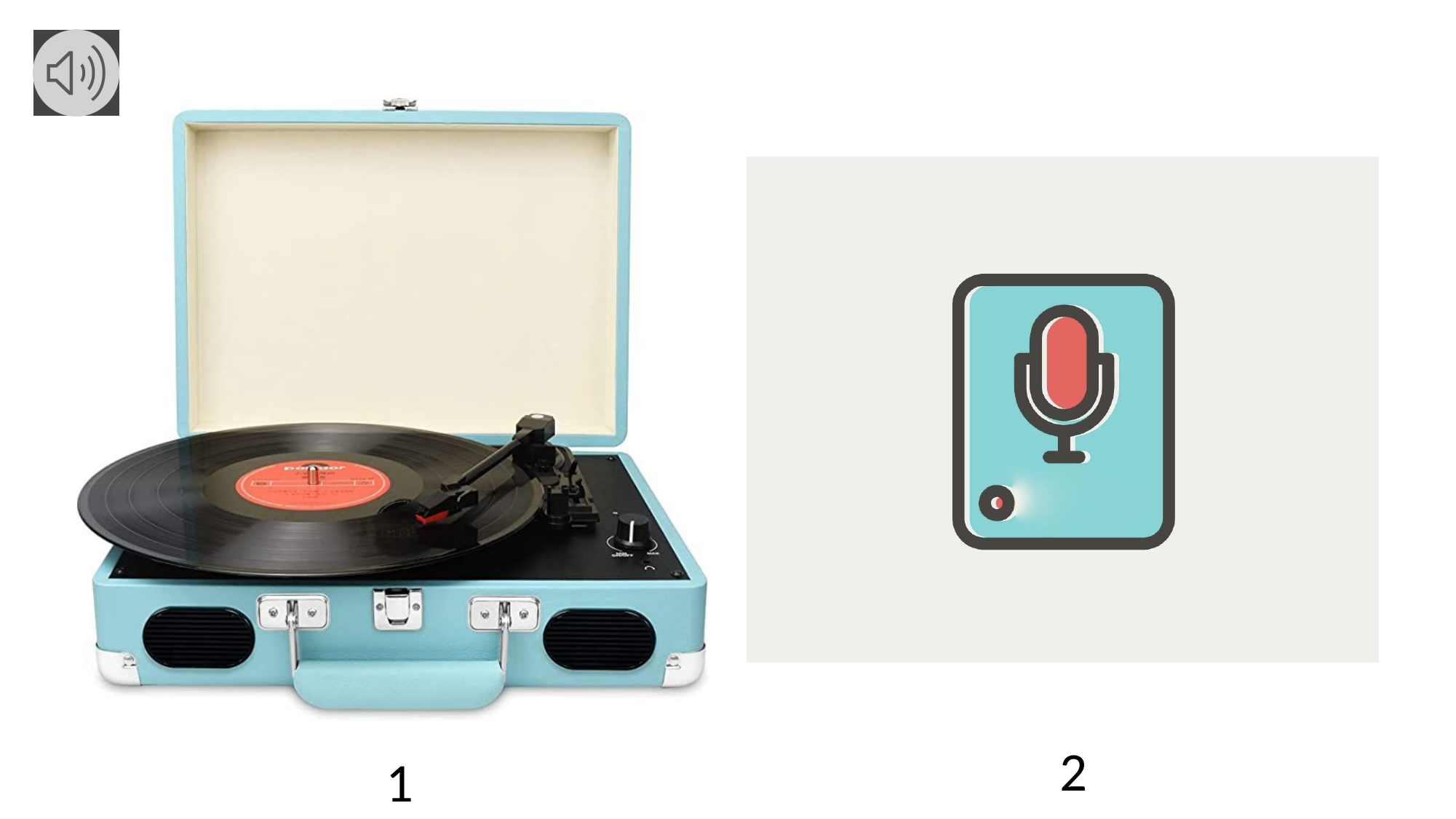

2
1

## Slide 24
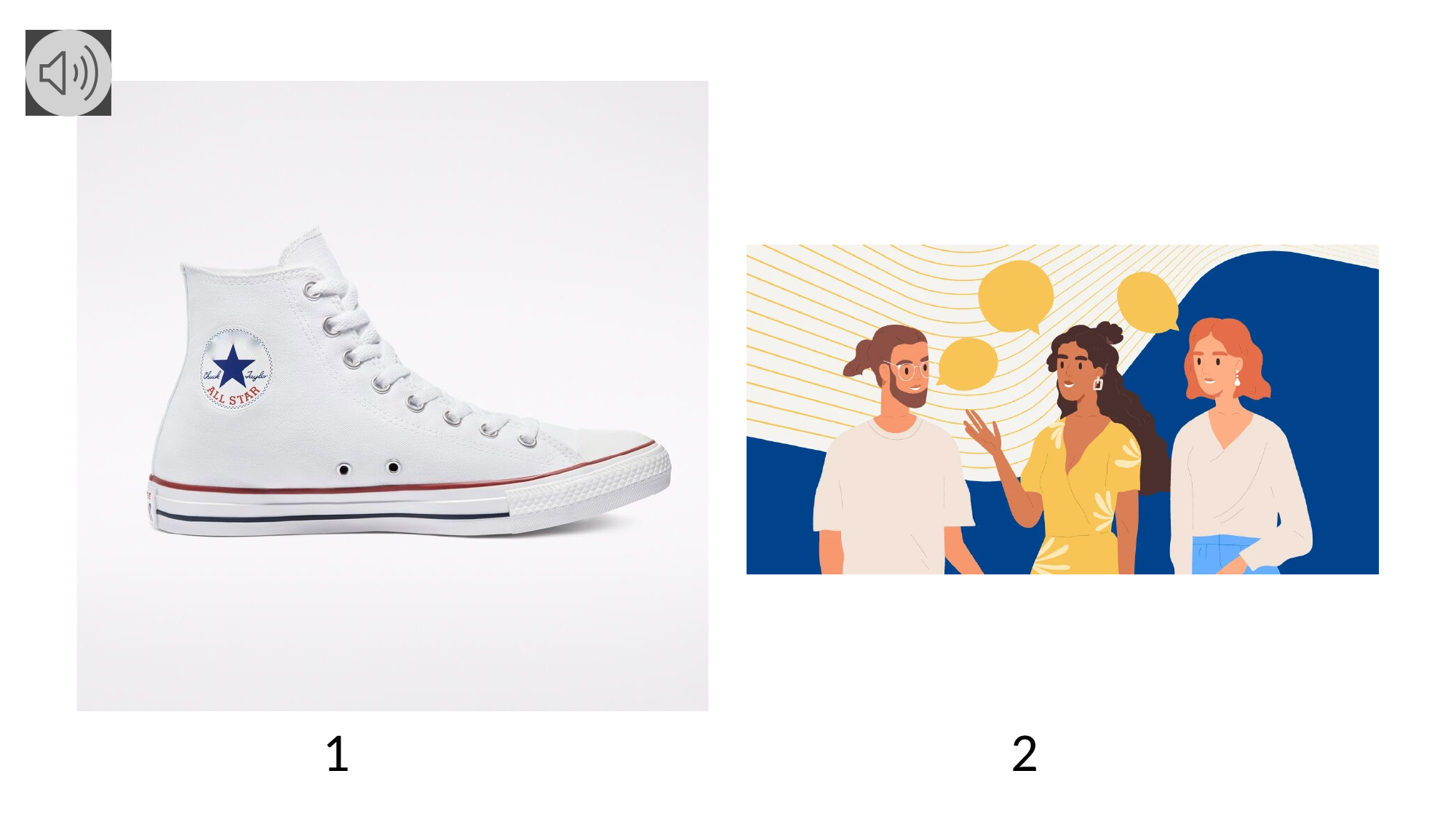

1
2

## Slide 25
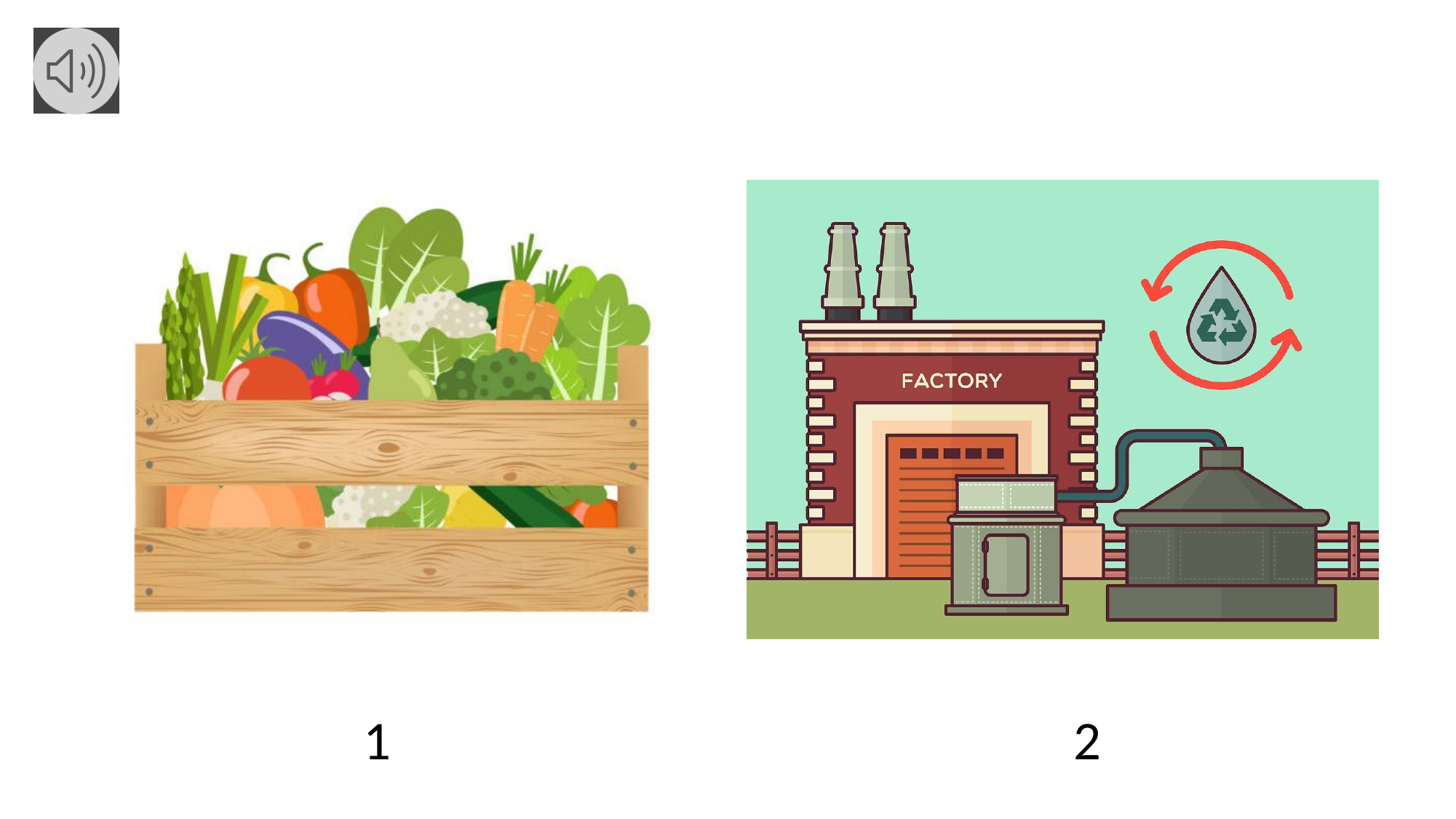

1
2

## Slide 26
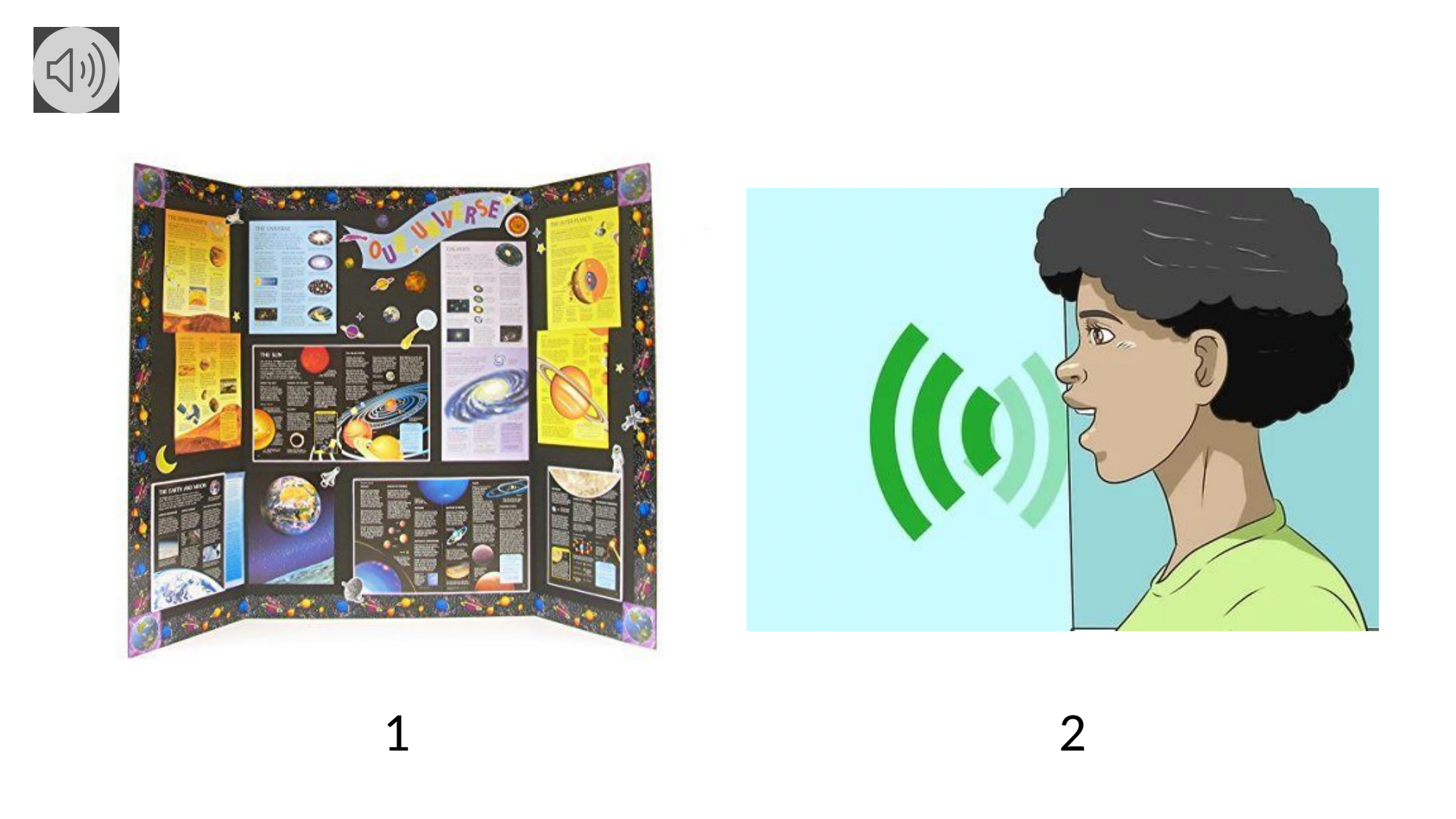

1
2

## Slide 27
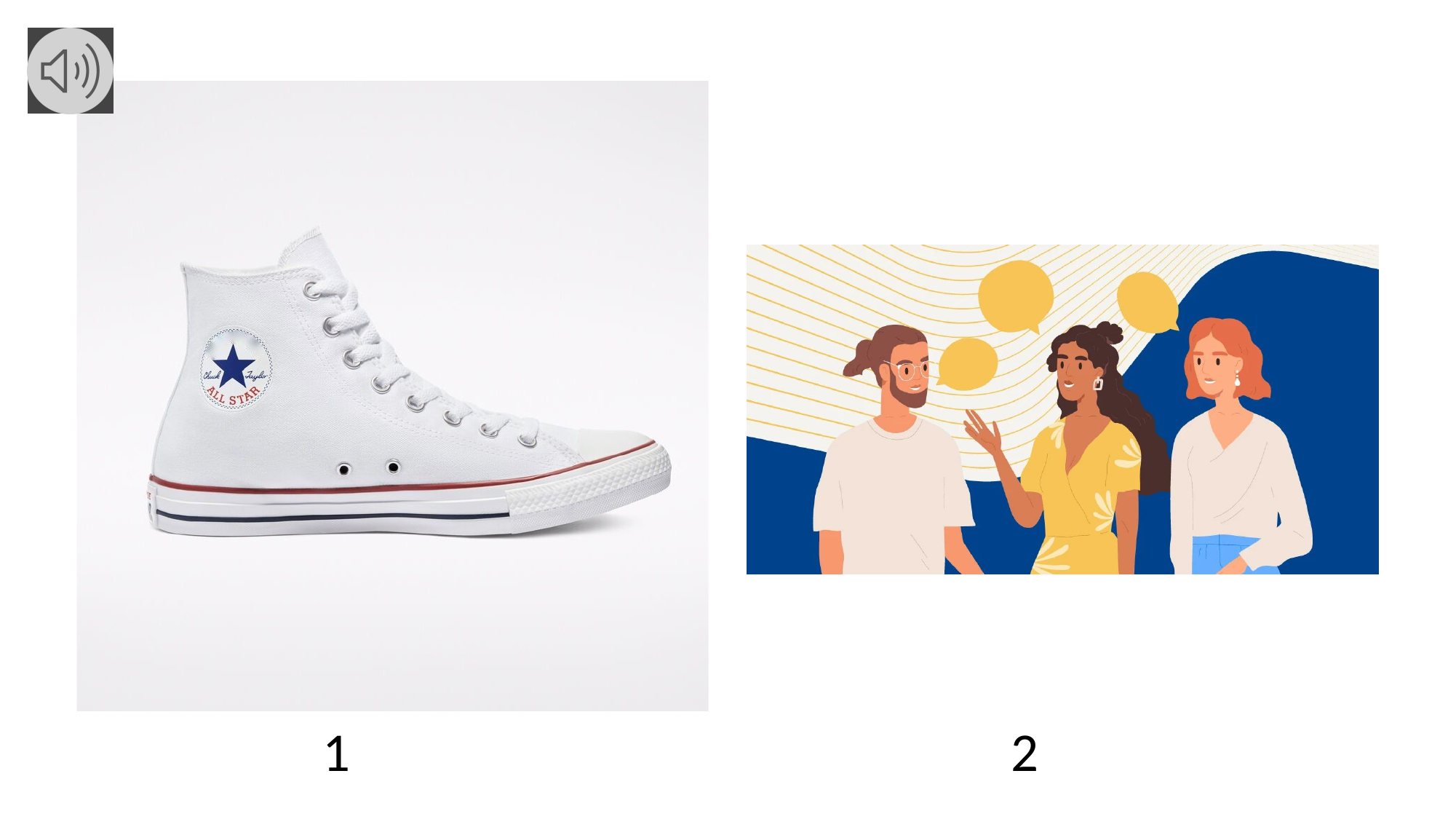

1
2
