# Supplementary material for: Understanding development of Mainstream US English lexical stress using semi-naturalistic stimuli
Source: PLoS One. 2026 Apr 29;21(4):e0345745. doi: 10.1371/journal.pone.0345745 (PMC13128110; doi:10.1371/journal.pone.0345745)
Supplement: S3 Files — (ZIP) [file pone.0345745.s001.zip › S3_NLSTaskFiles/NLS-ExpressiveTaskRecordForm.pdf]

Communication, Acquisition, and Translational Studies Laboratory

NLS Expressive Task Record Form

Participant ID: _____________

Scoring: 1 = first syllable lexical stress; 2 = second syllable stress; A = ambiguous/unscorable

Indicate sentence position when scoring: *initial, medial, or final*

**Stimuli**	**Initial**	**Medial**	**Final**
Canoes			
Monkey			
Guitar			
Bubbles			
Giraffe			
Turtle			
Cookie			
Raccoon			
Chicken			
Balloon			
Elephant			
Butterfly			
Banana			
Spaghetti			

Please reach out to Jill Thorson, University of New Hampshire, with any questions: jill.thorson@unh.edu
