# Supplementary material for: Understanding development of Mainstream US English lexical stress using semi-naturalistic stimuli
Source: PLoS One. 2026 Apr 29;21(4):e0345745. doi: 10.1371/journal.pone.0345745 (PMC13128110; doi:10.1371/journal.pone.0345745)
Supplement: S3 Files — (ZIP) [file pone.0345745.s001.zip › S3_NLSTaskFiles/NLS-ReceptiveTaskPresentation.pdf]

NLS Receptive Task

Vocabulary Check

converse (noun)

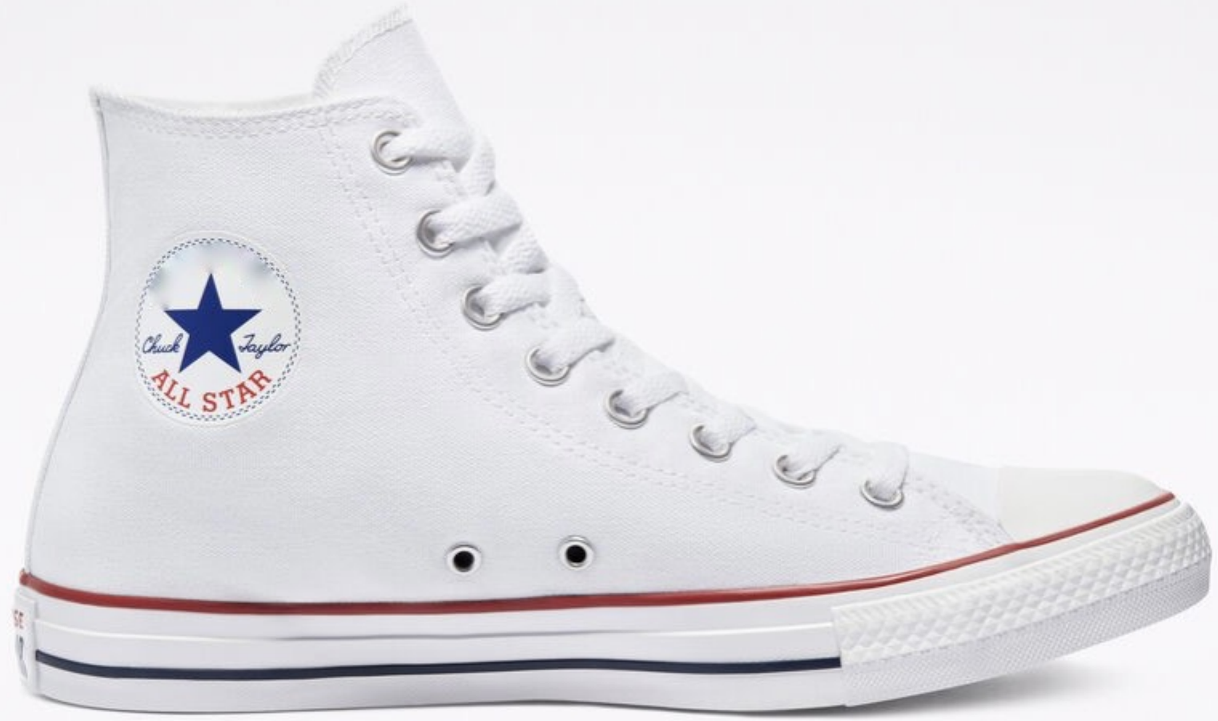

converse (verb)

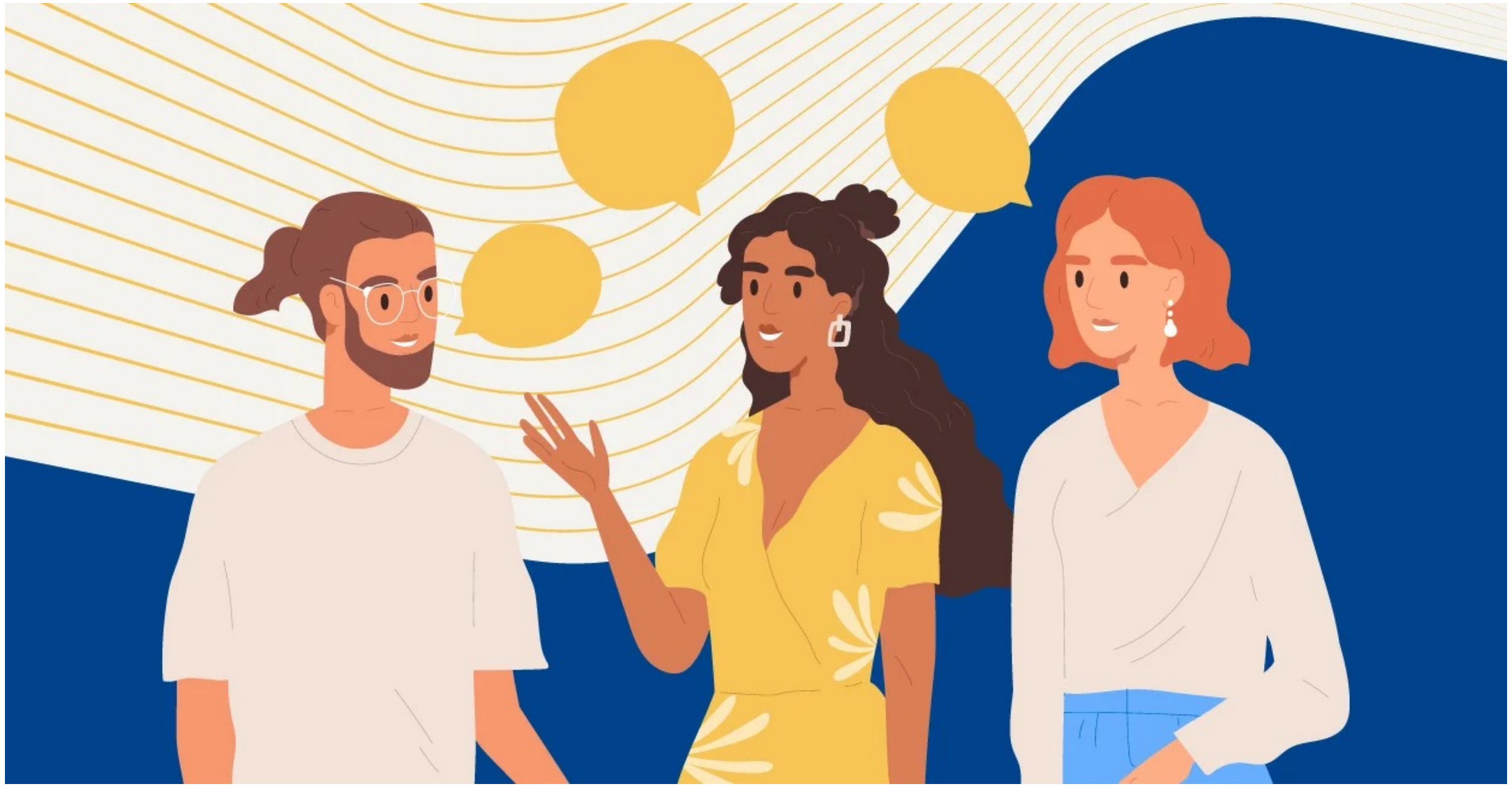

project (noun)

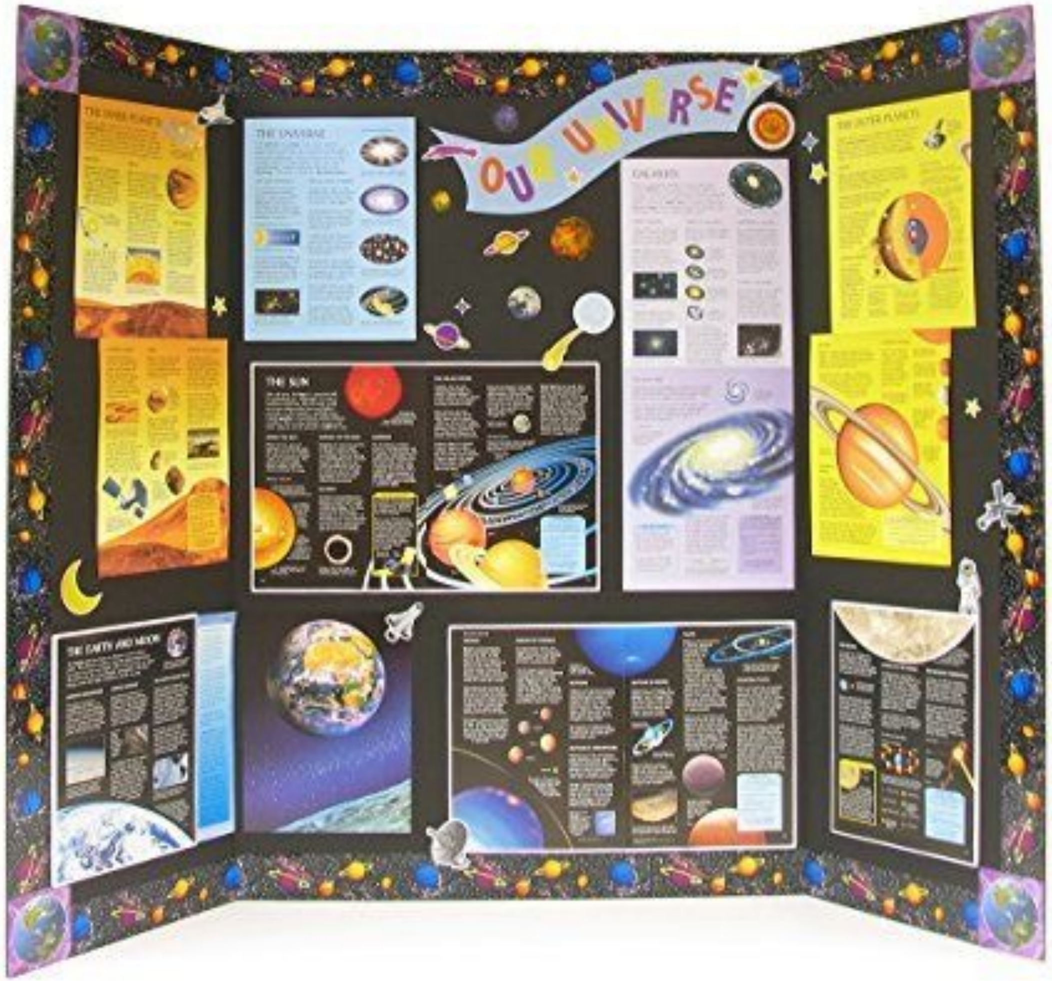

project (verb)

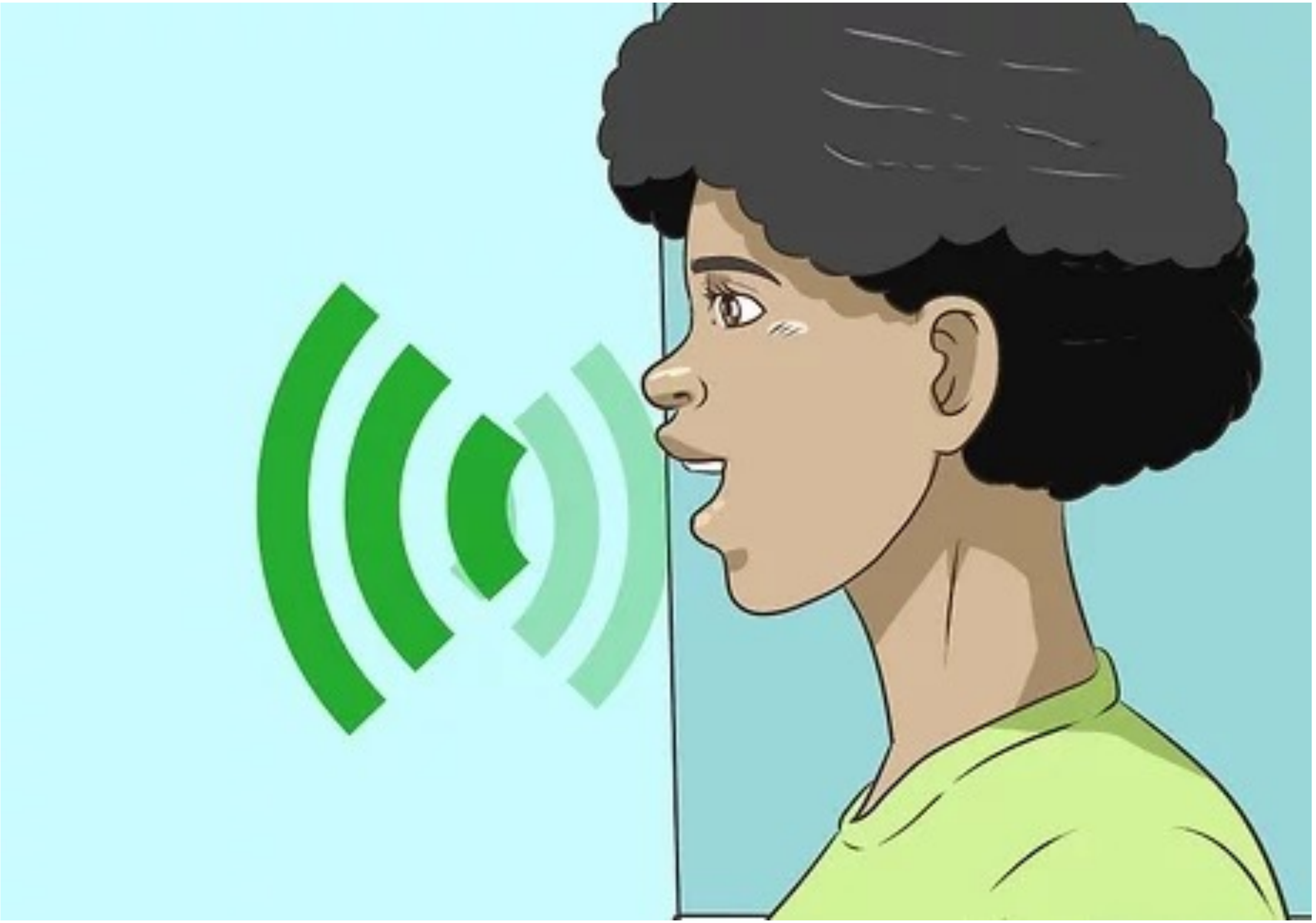

produce
(noun)

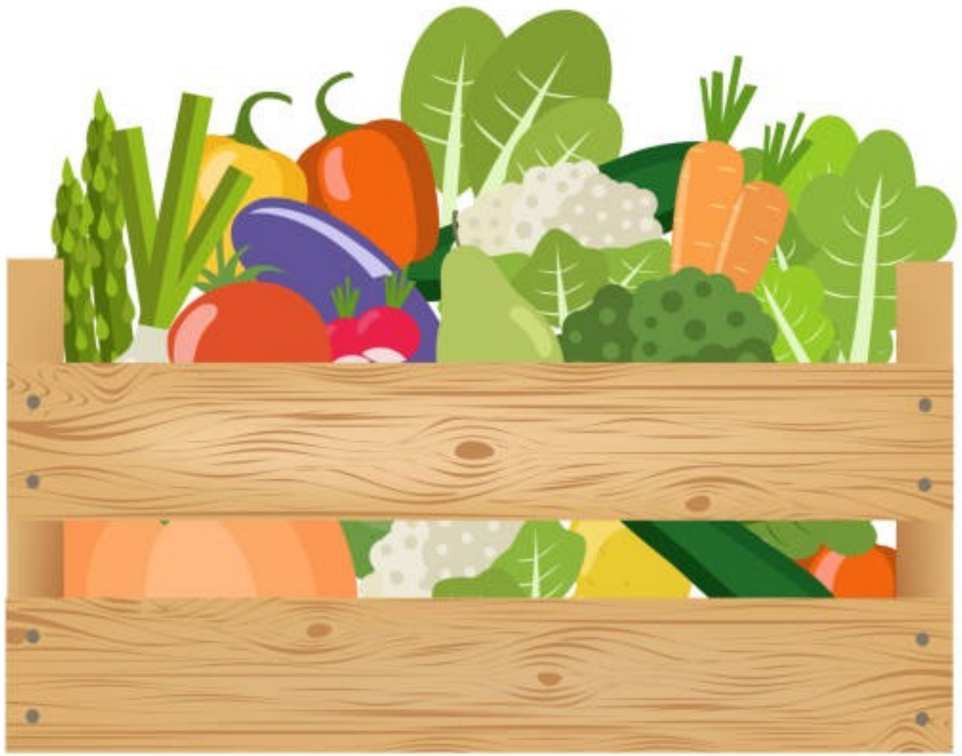

produce
(verb)

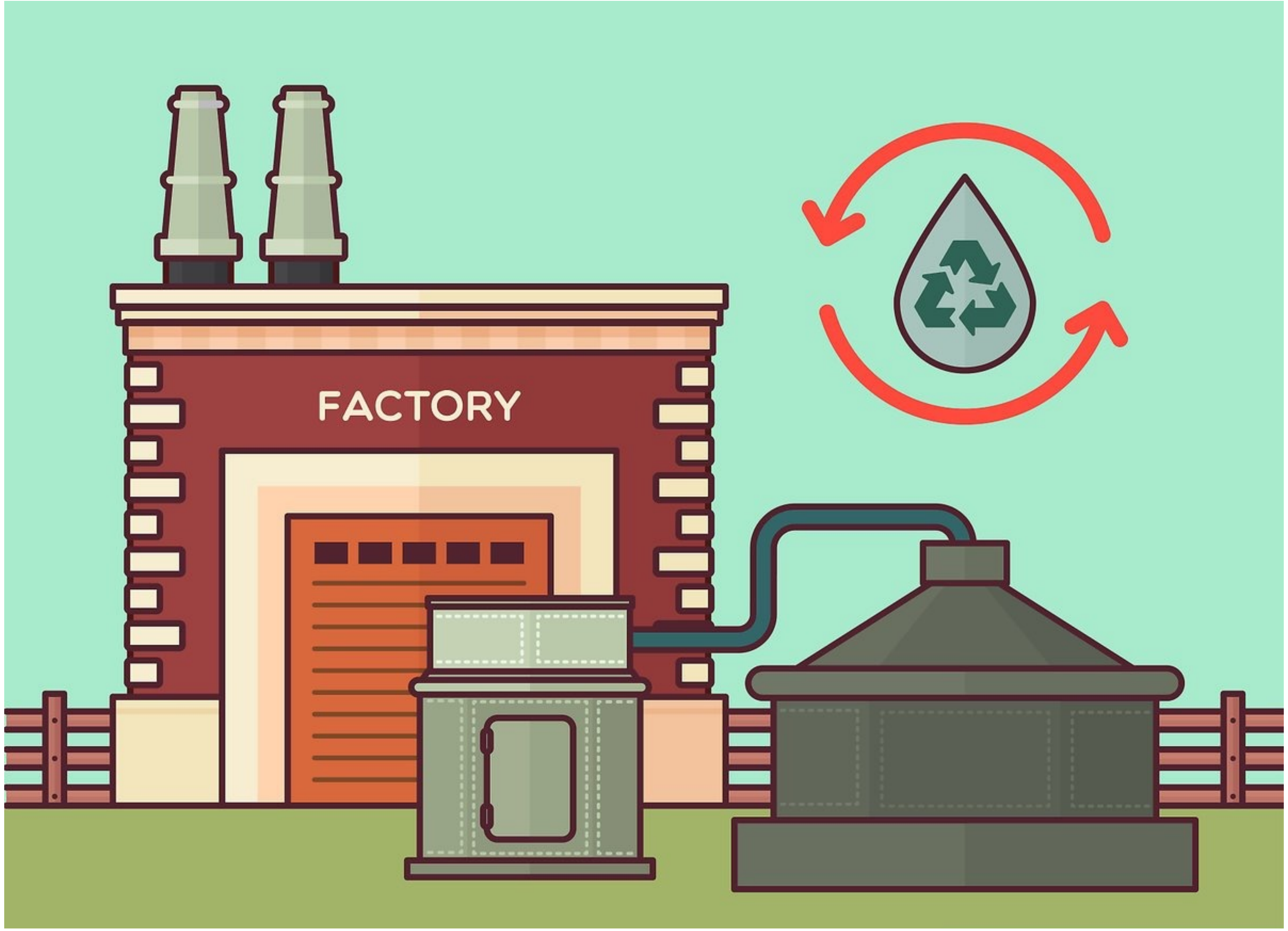

present (noun)

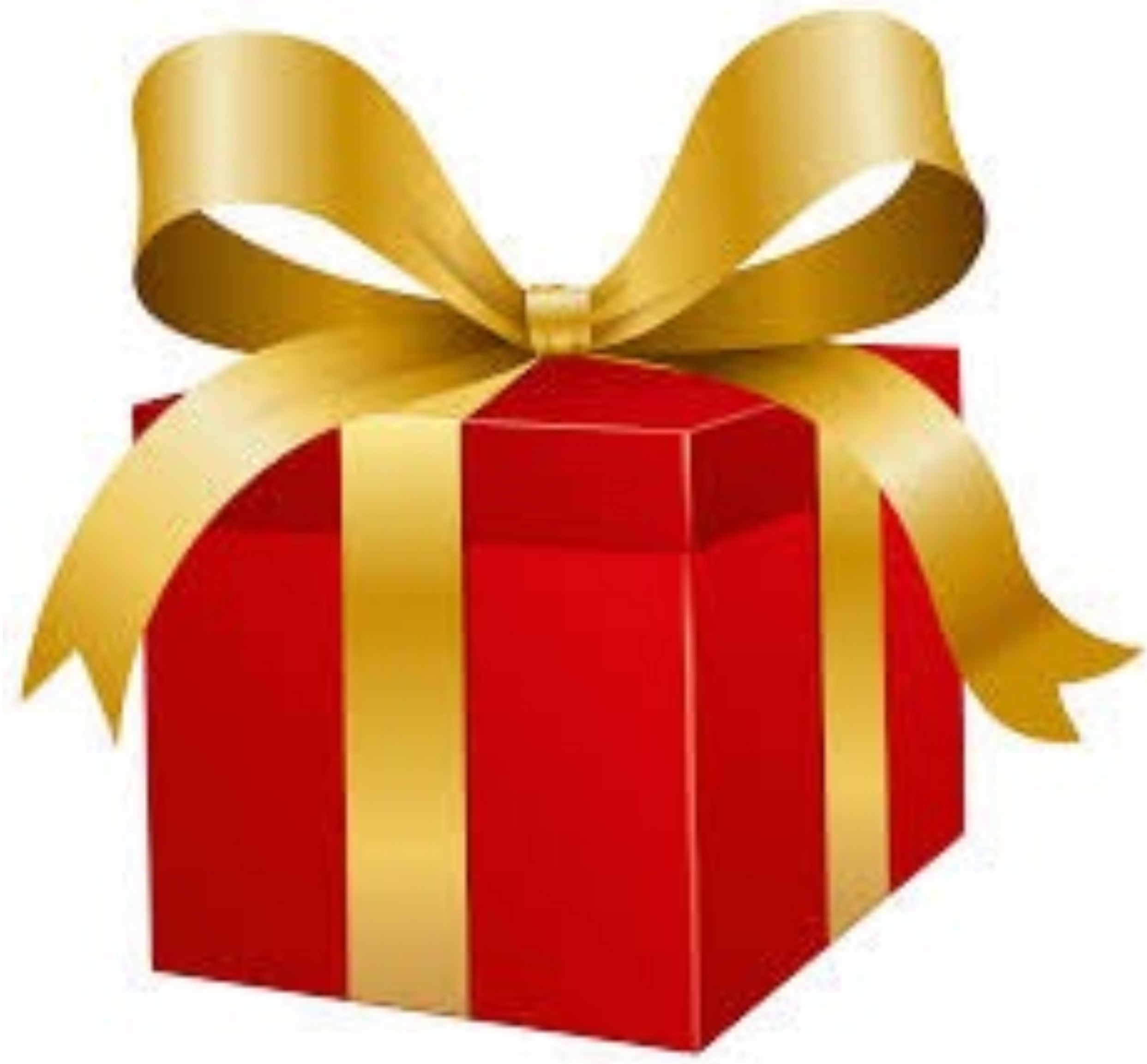

present
(verb)

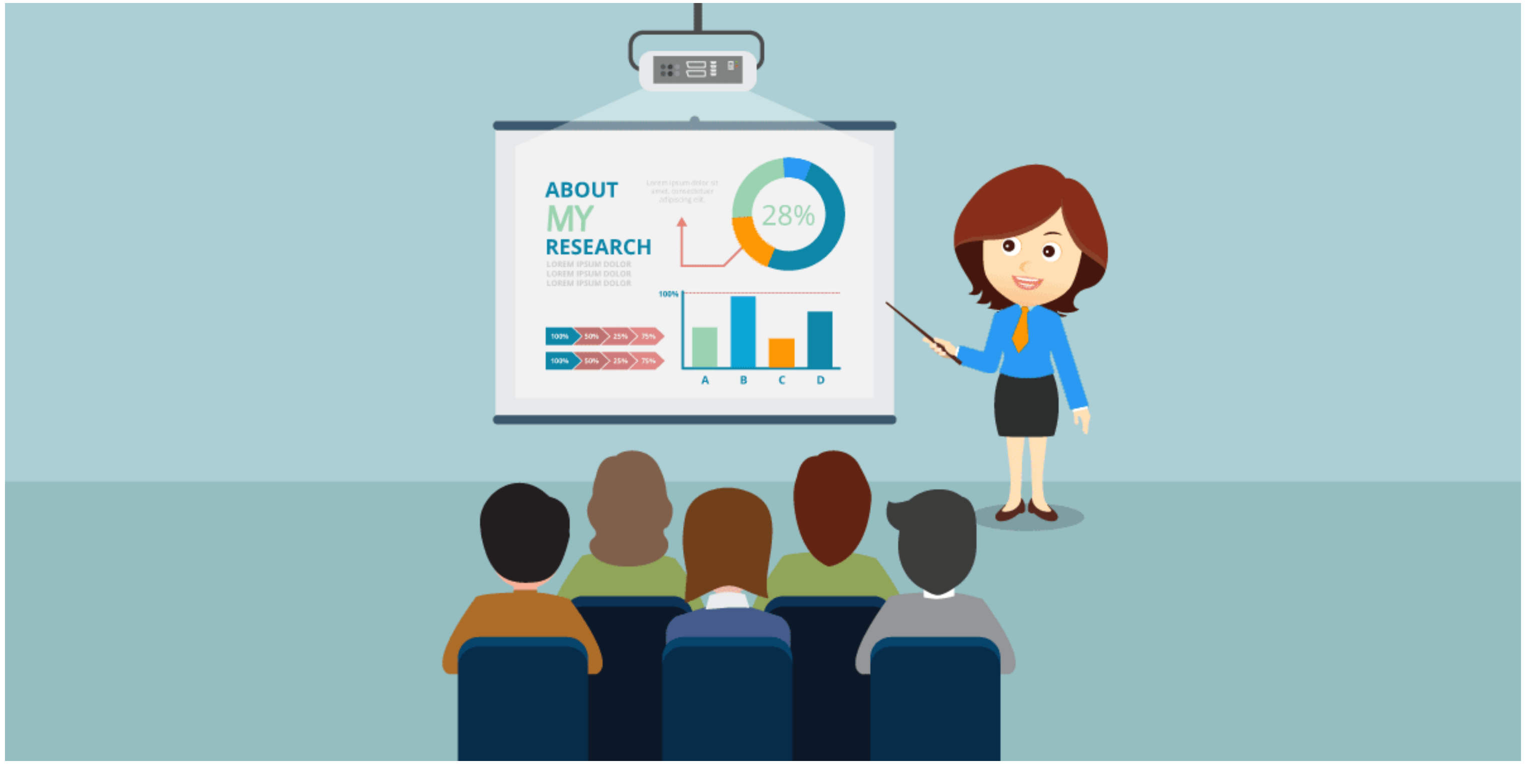

record (noun)

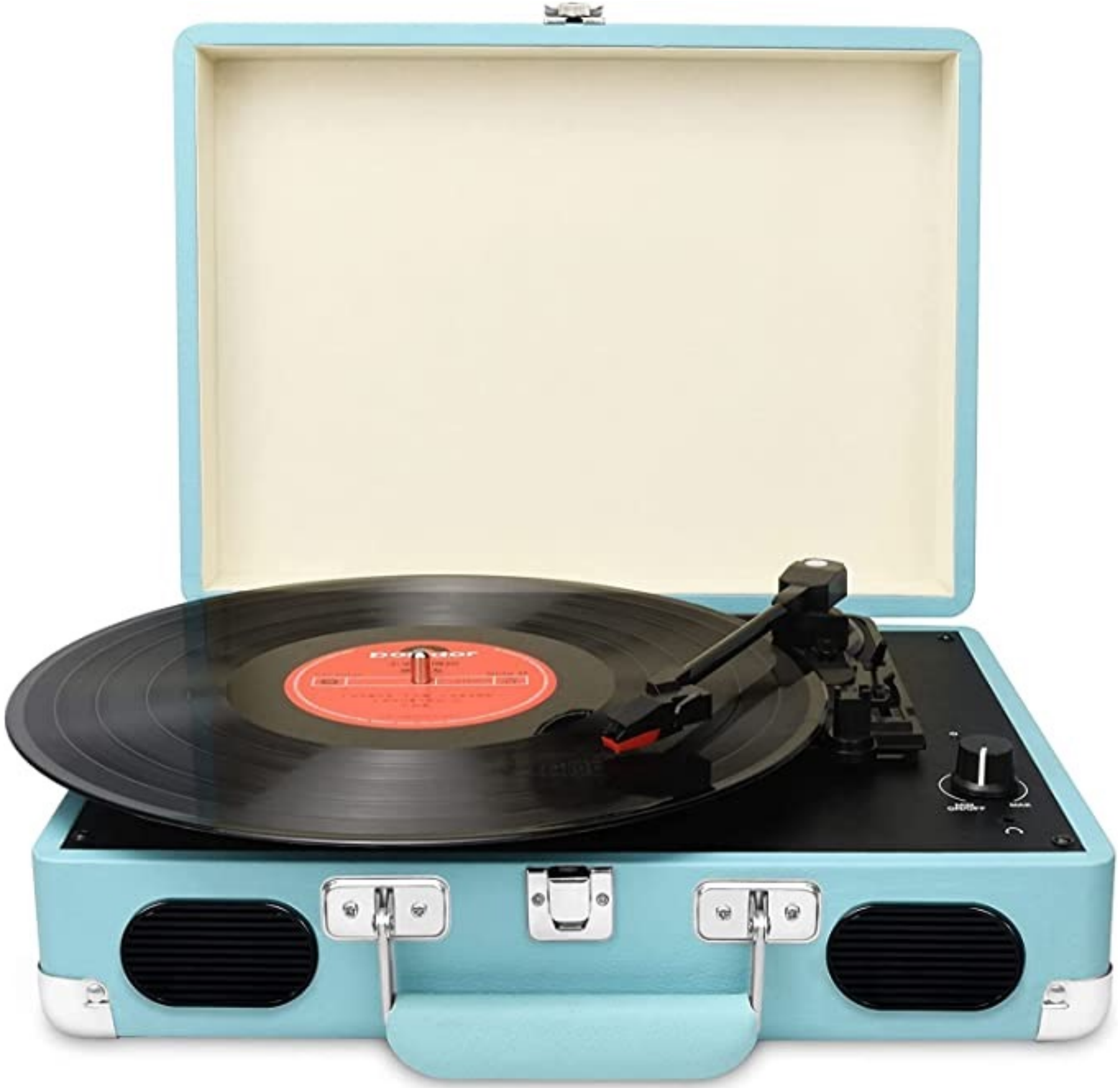

record
(verb)

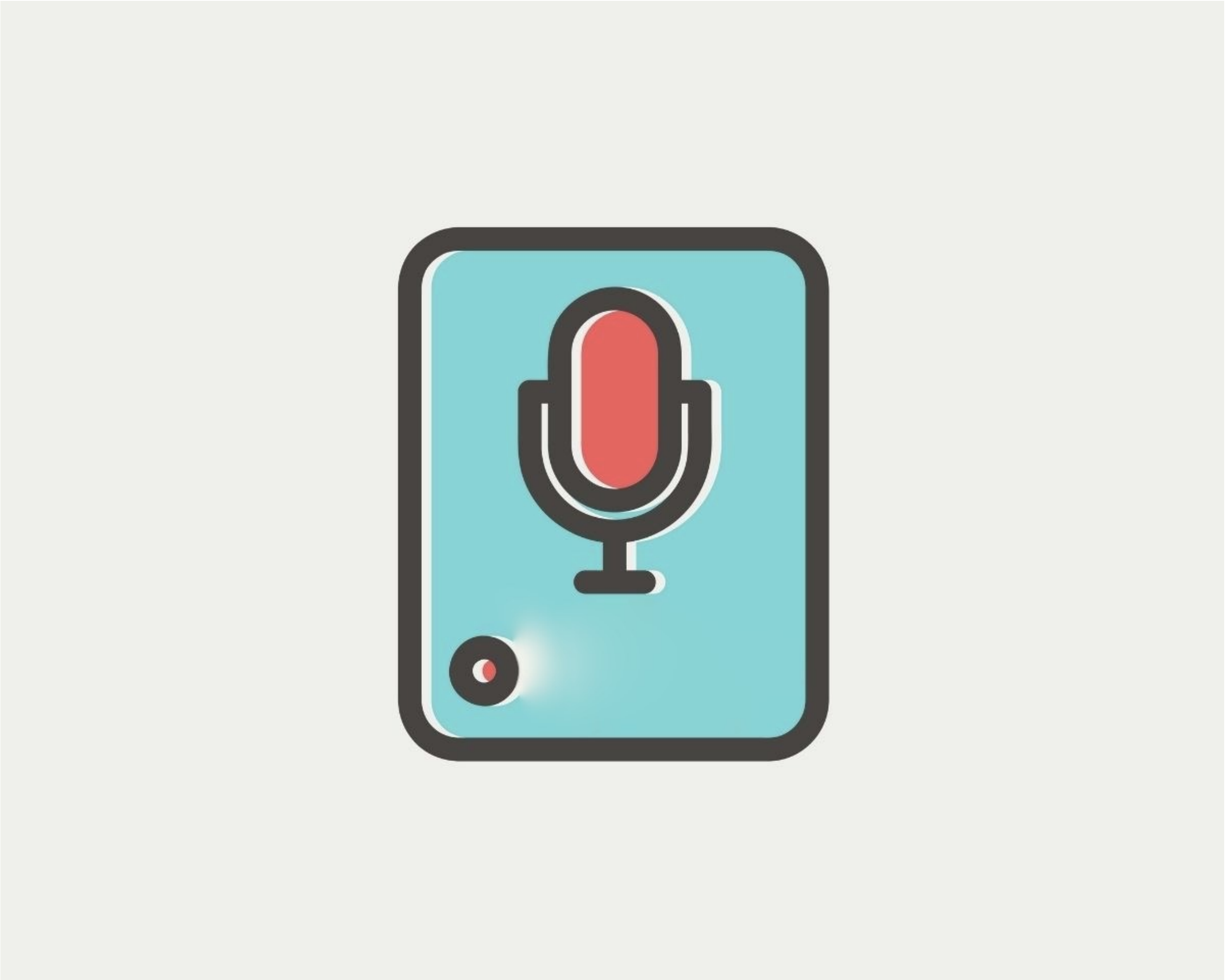

object
(noun)

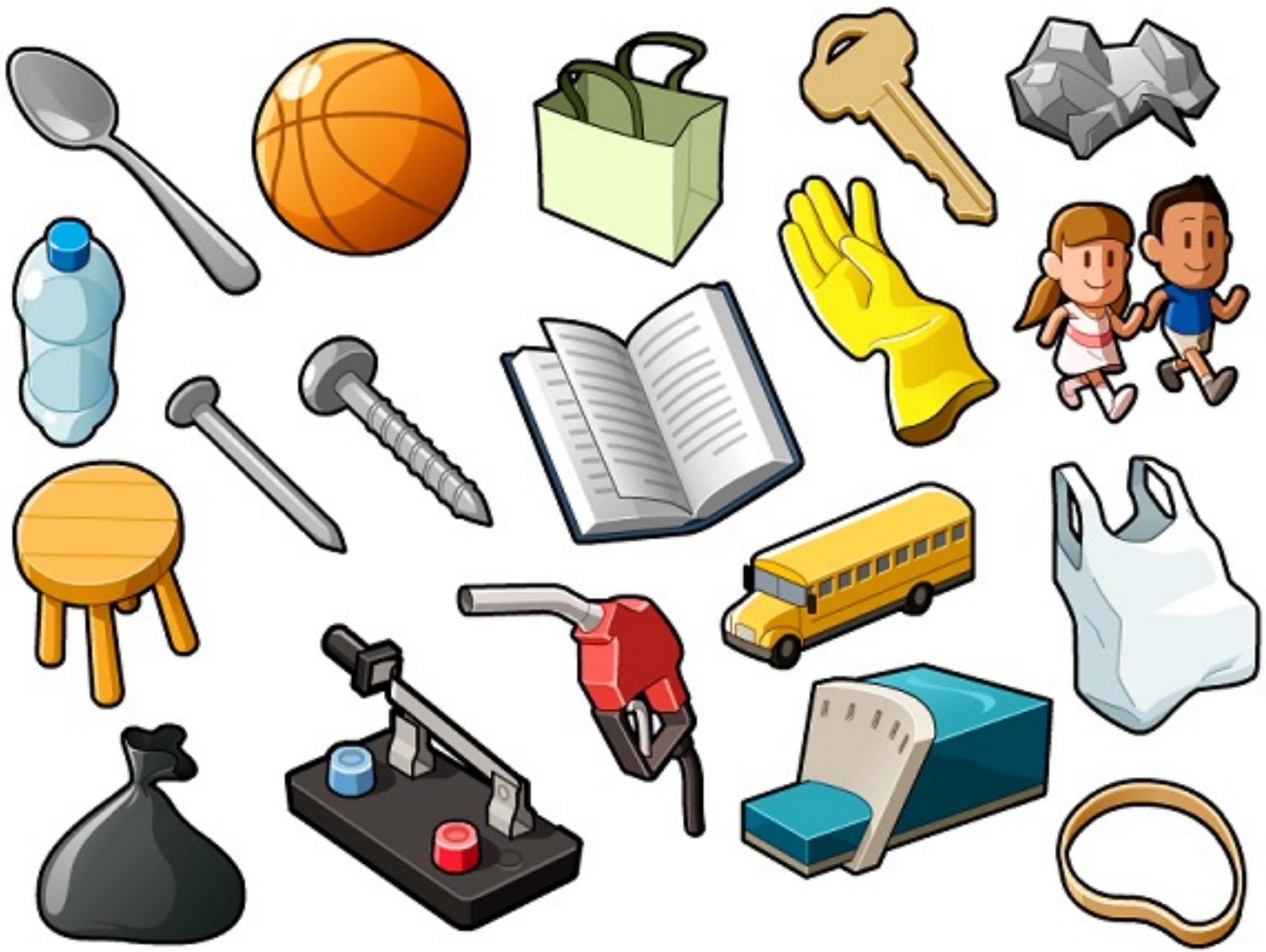

object
(verb)

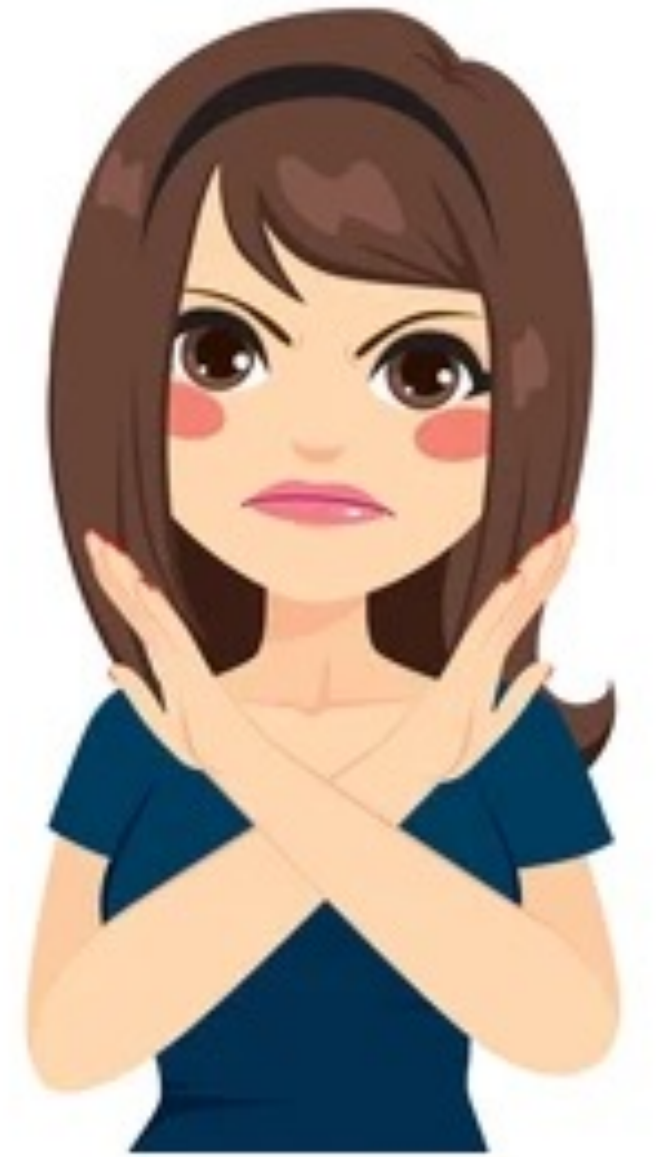

Pick which word you hear

1 or 2

____

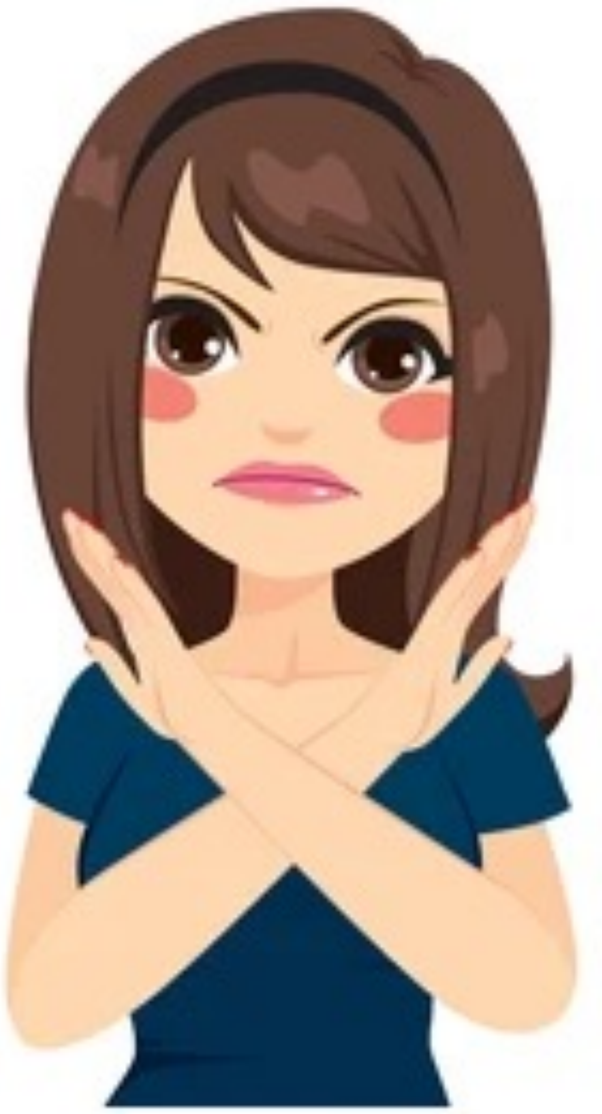

1

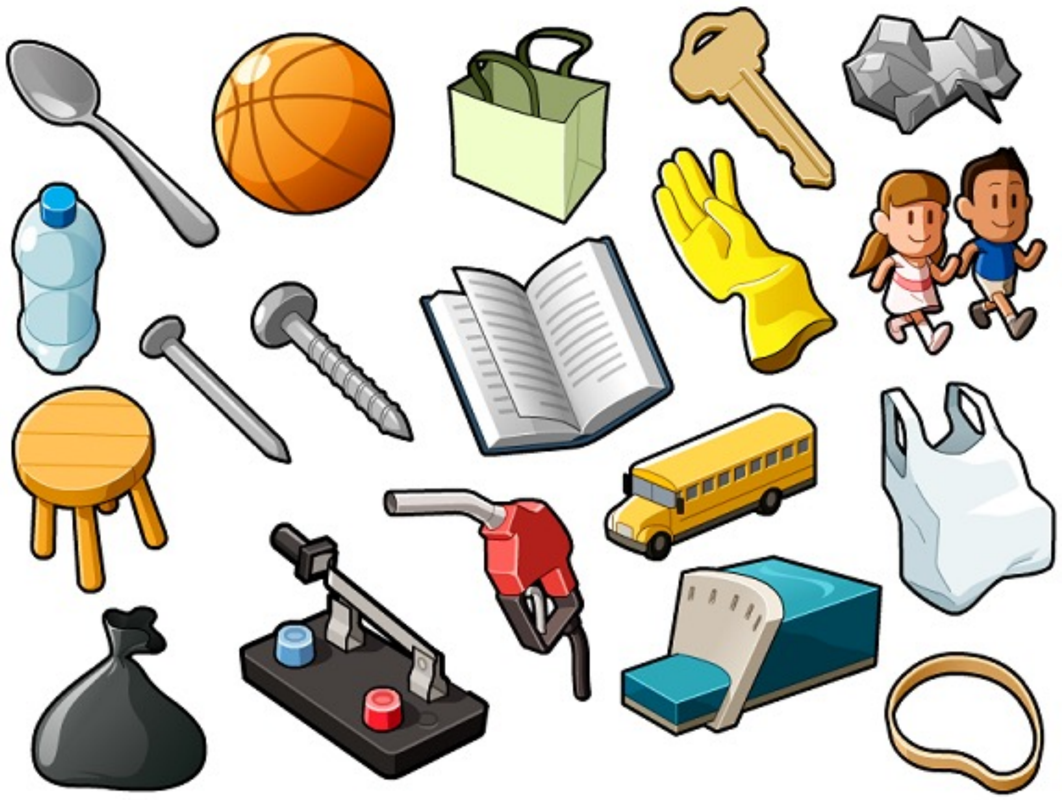

2

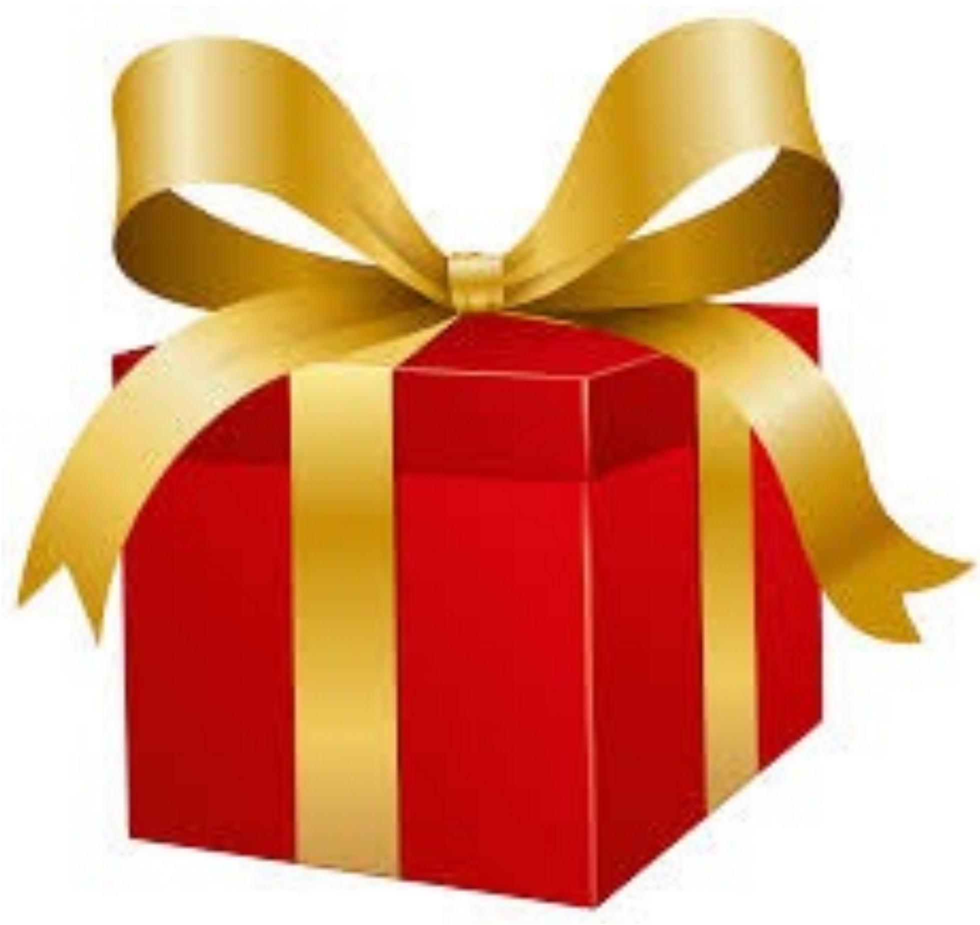

1

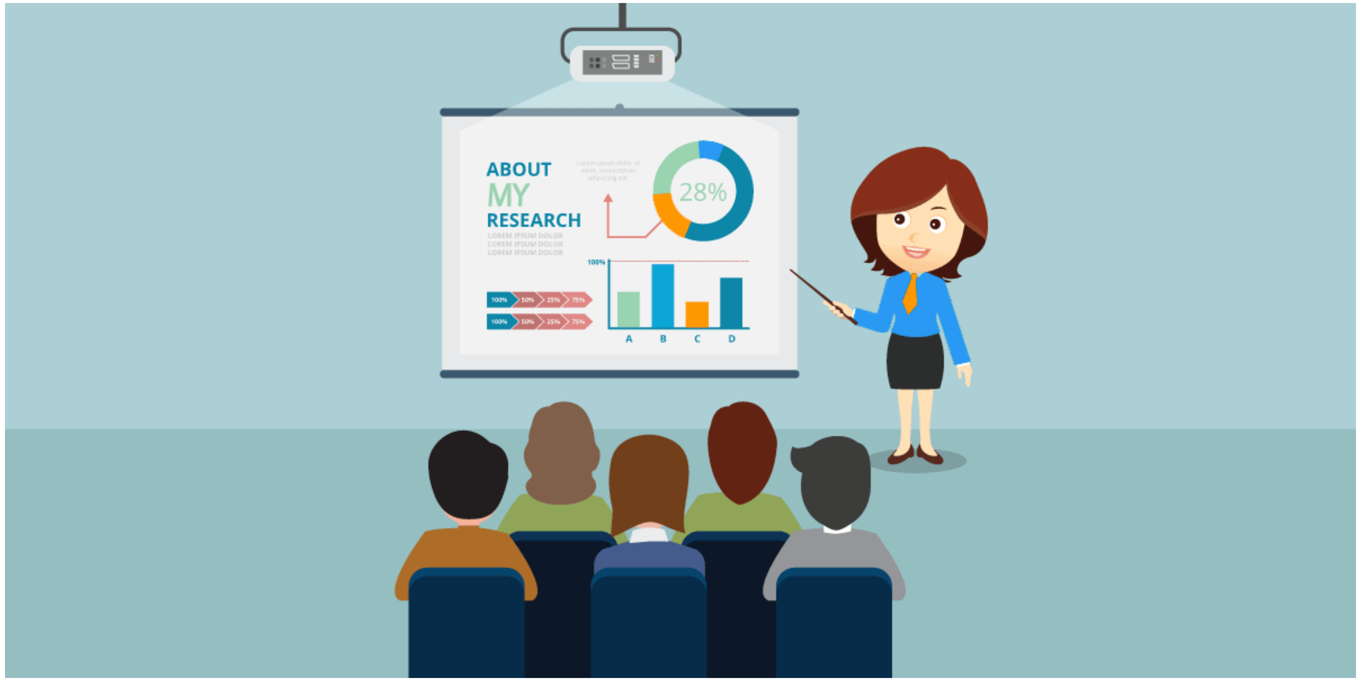

2

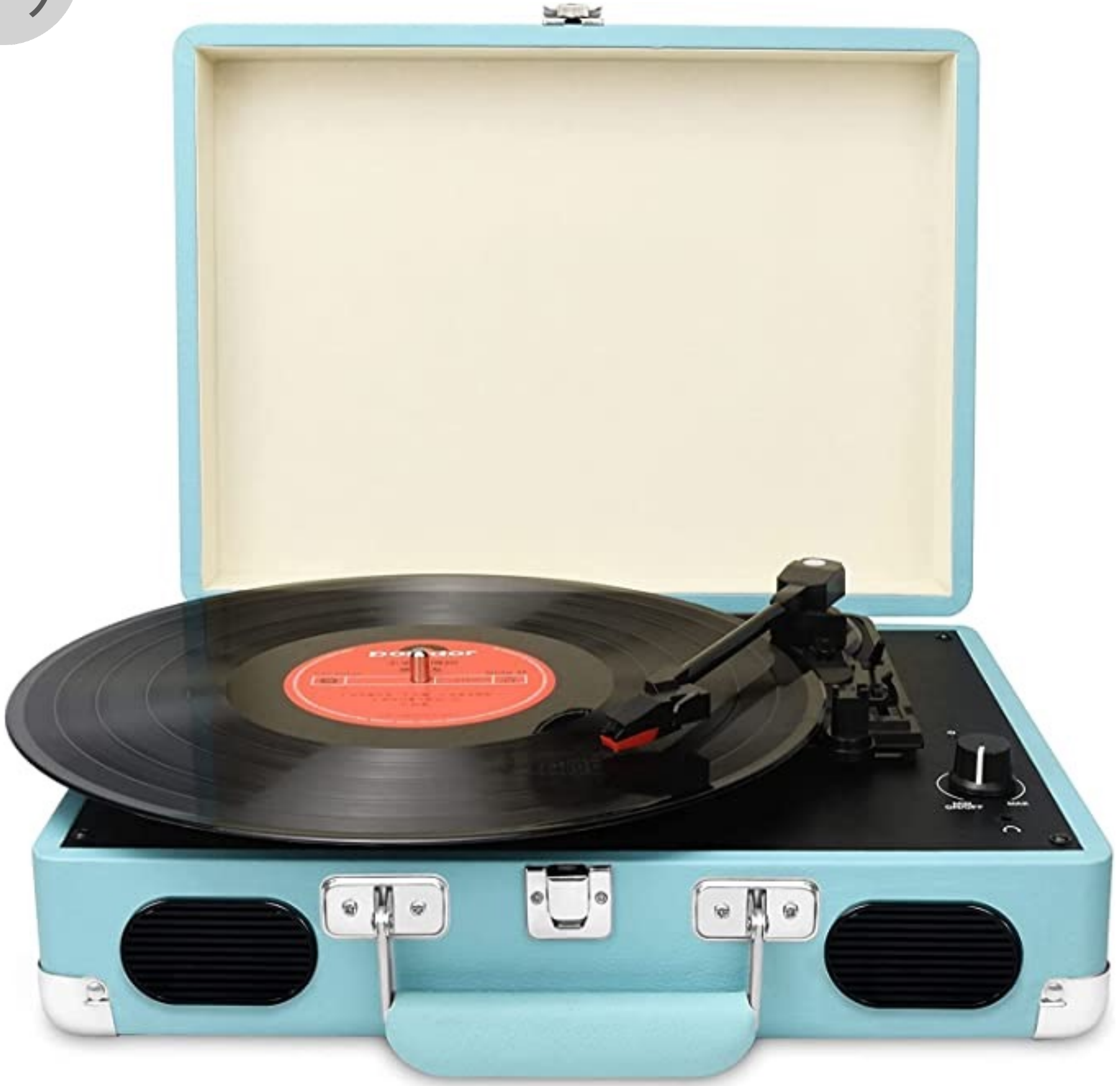

1

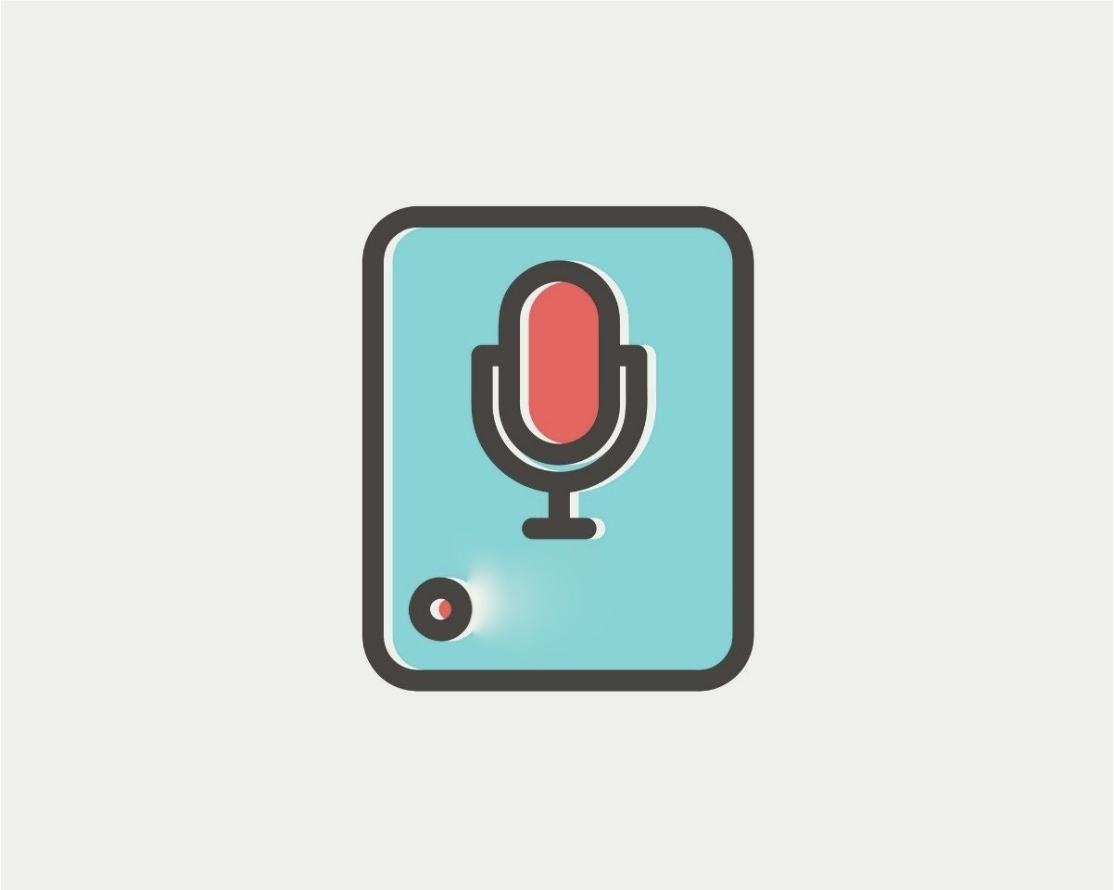

2

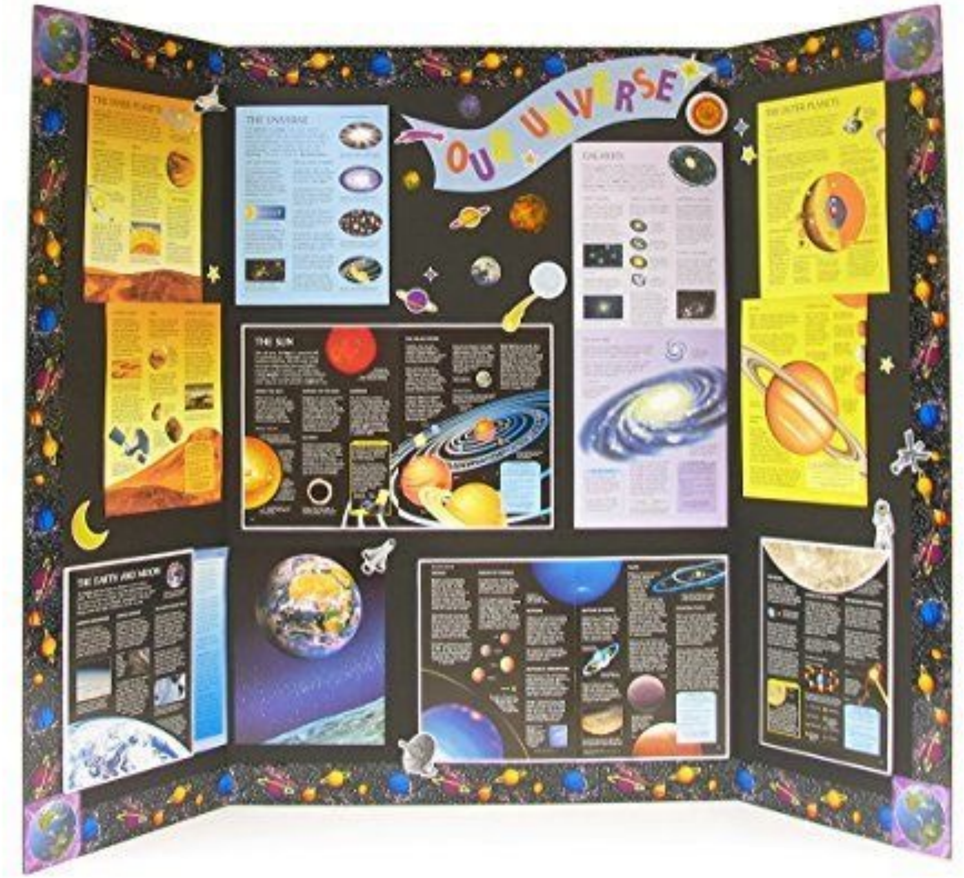

1

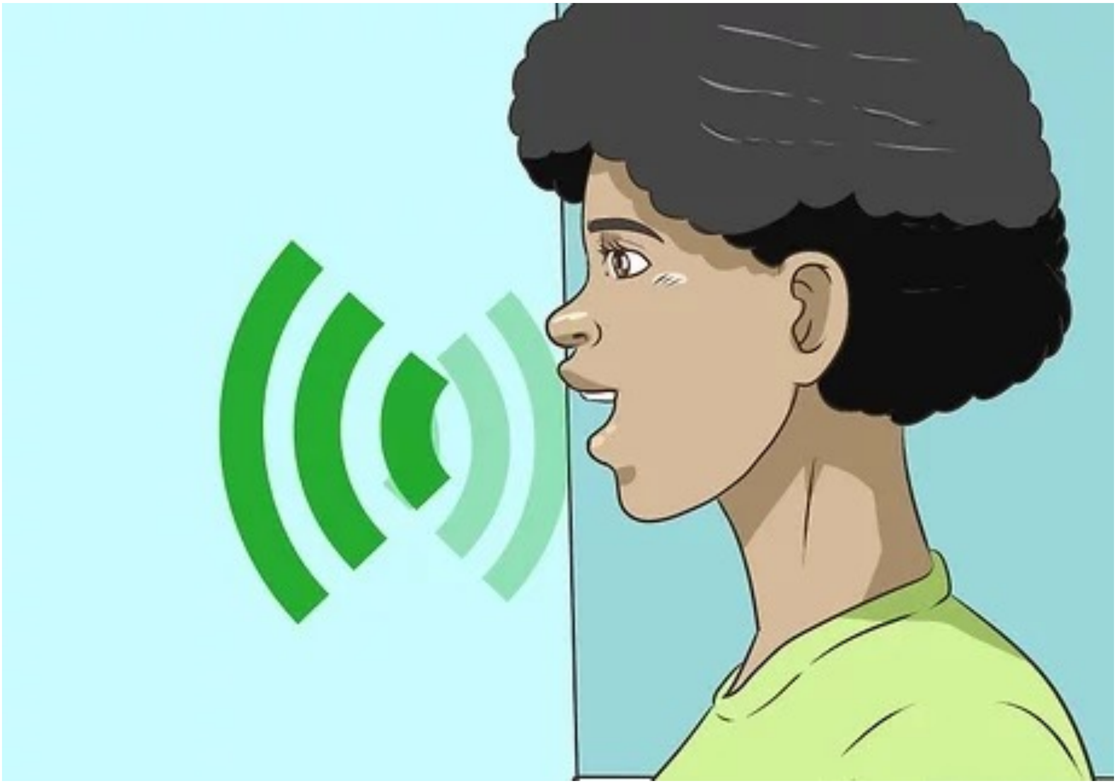

2

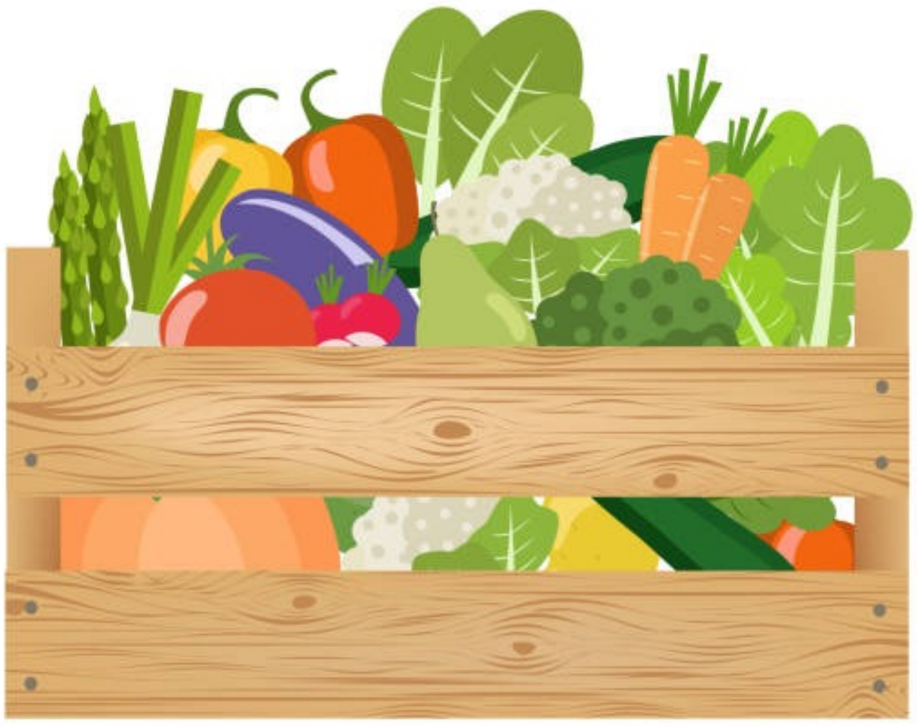

1

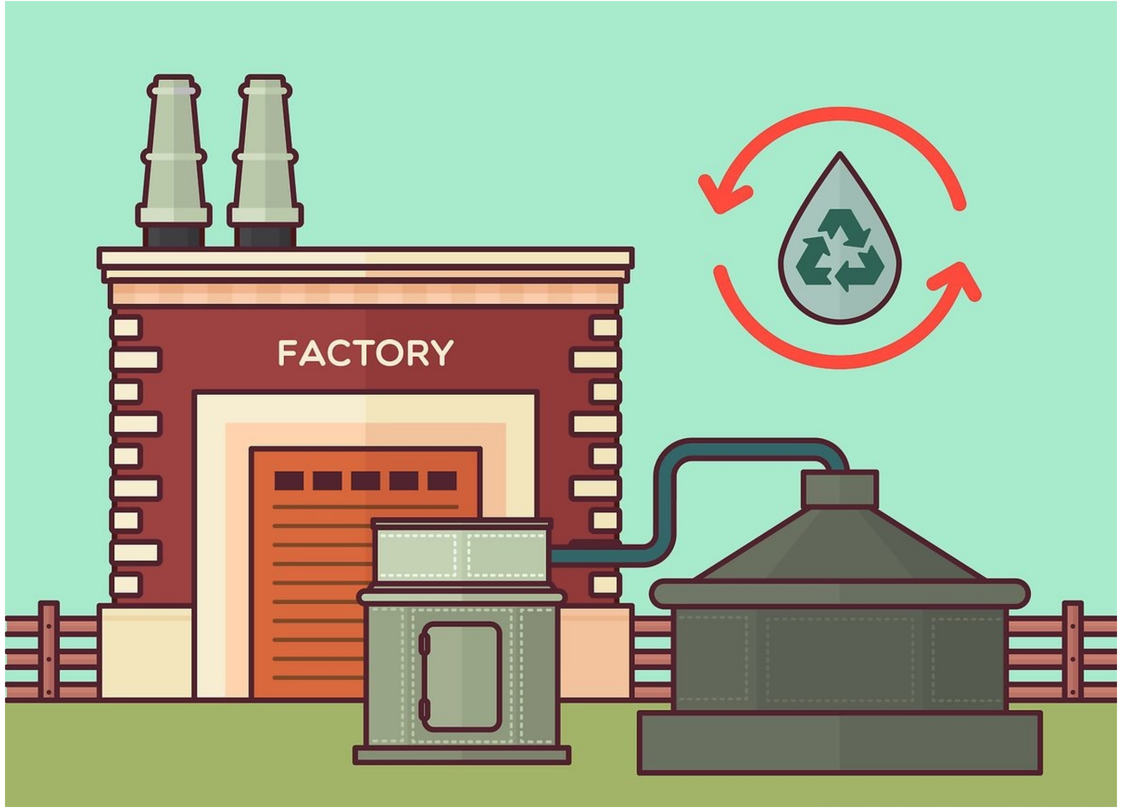

2

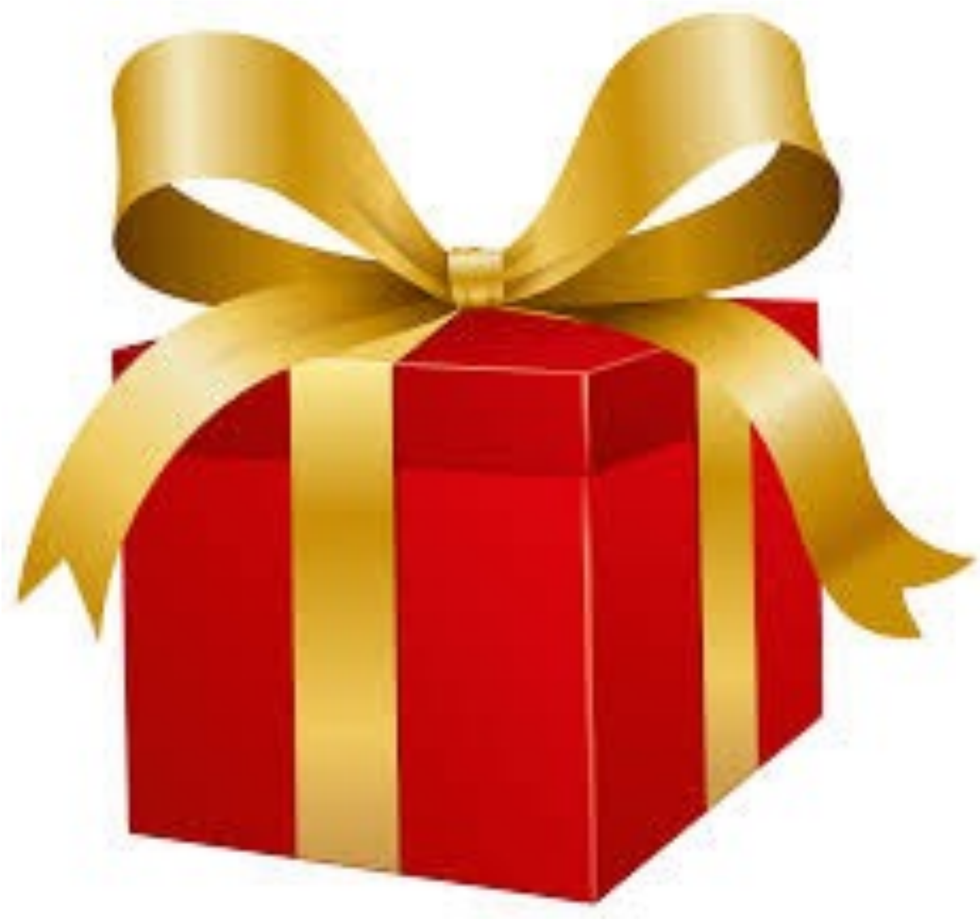

1

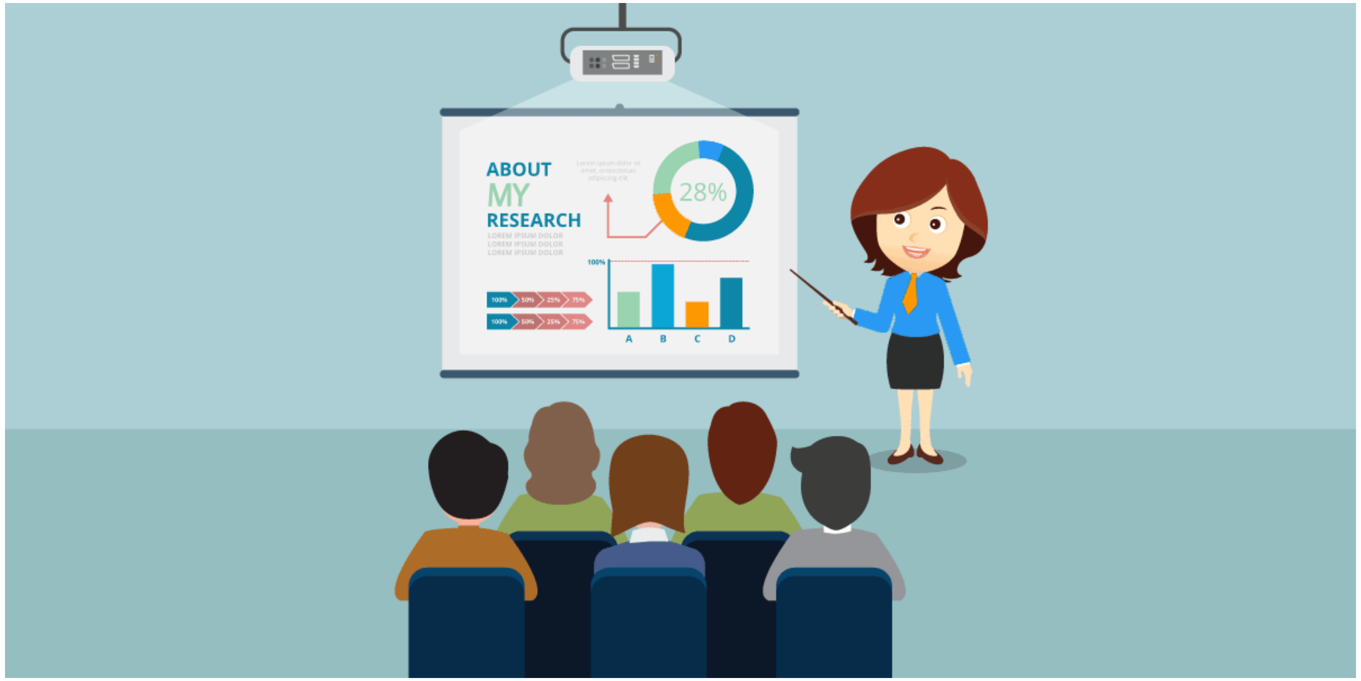

2

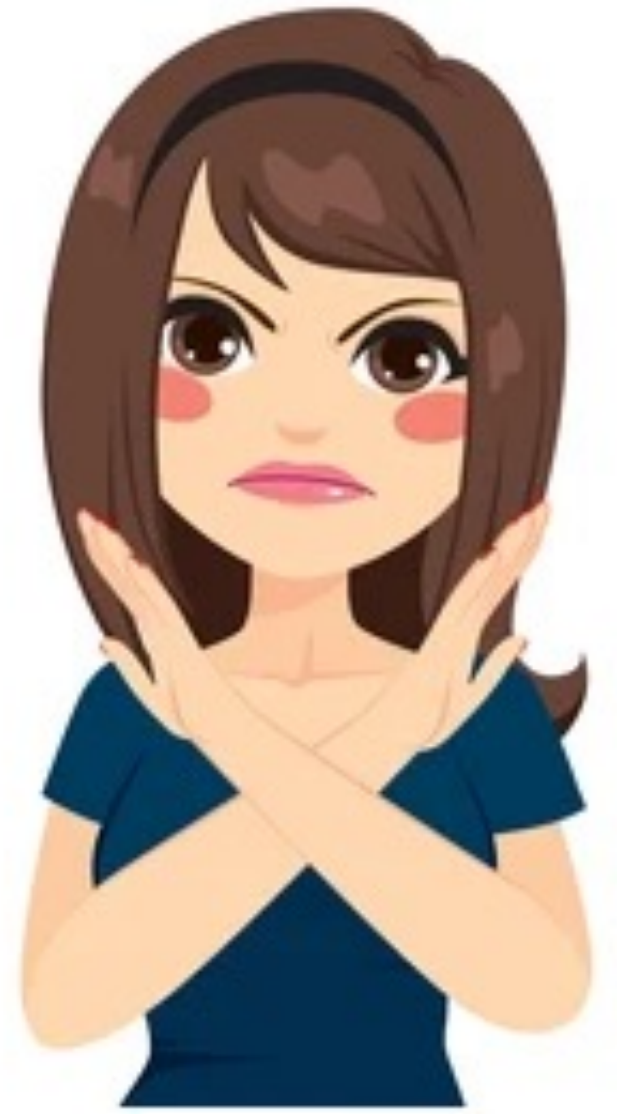

1

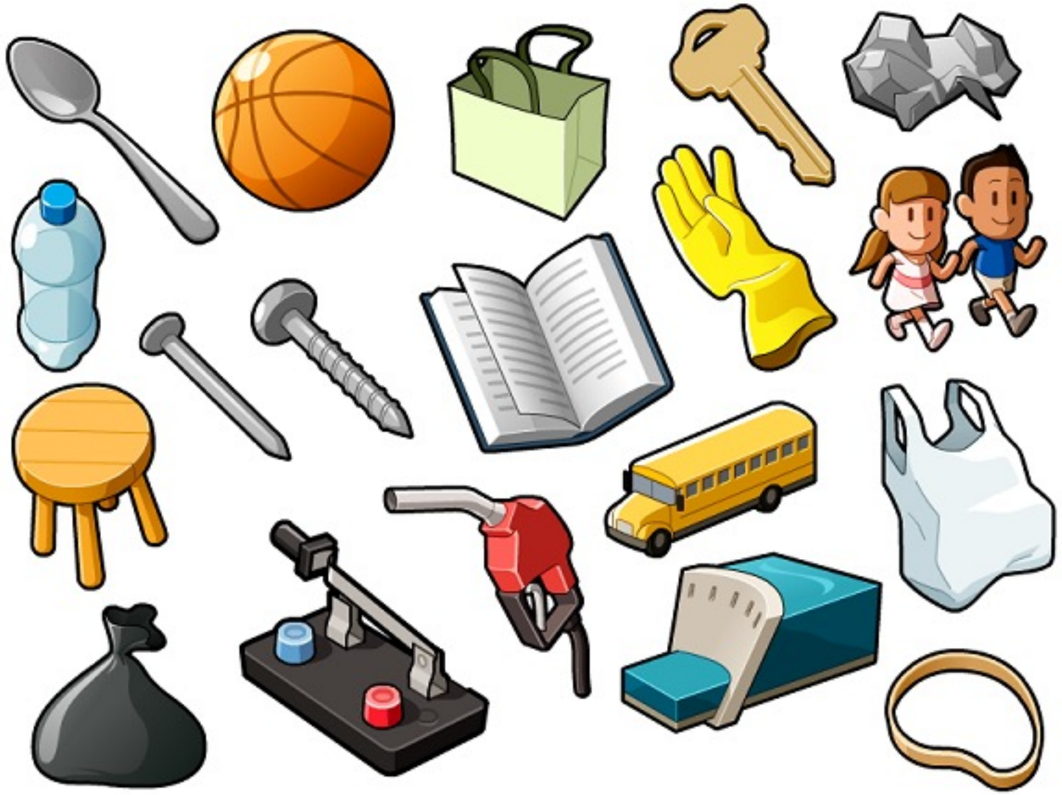

2

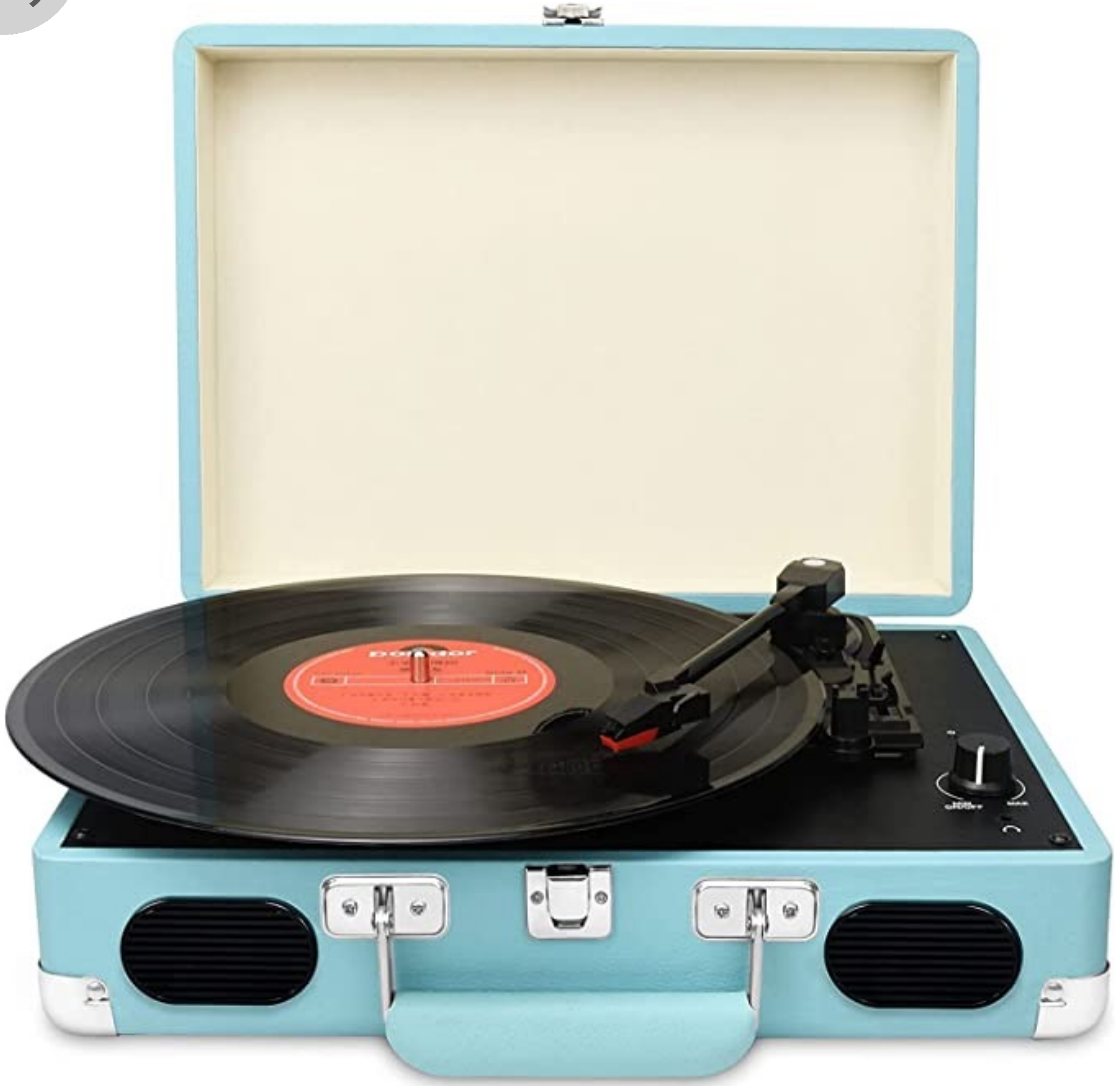

1

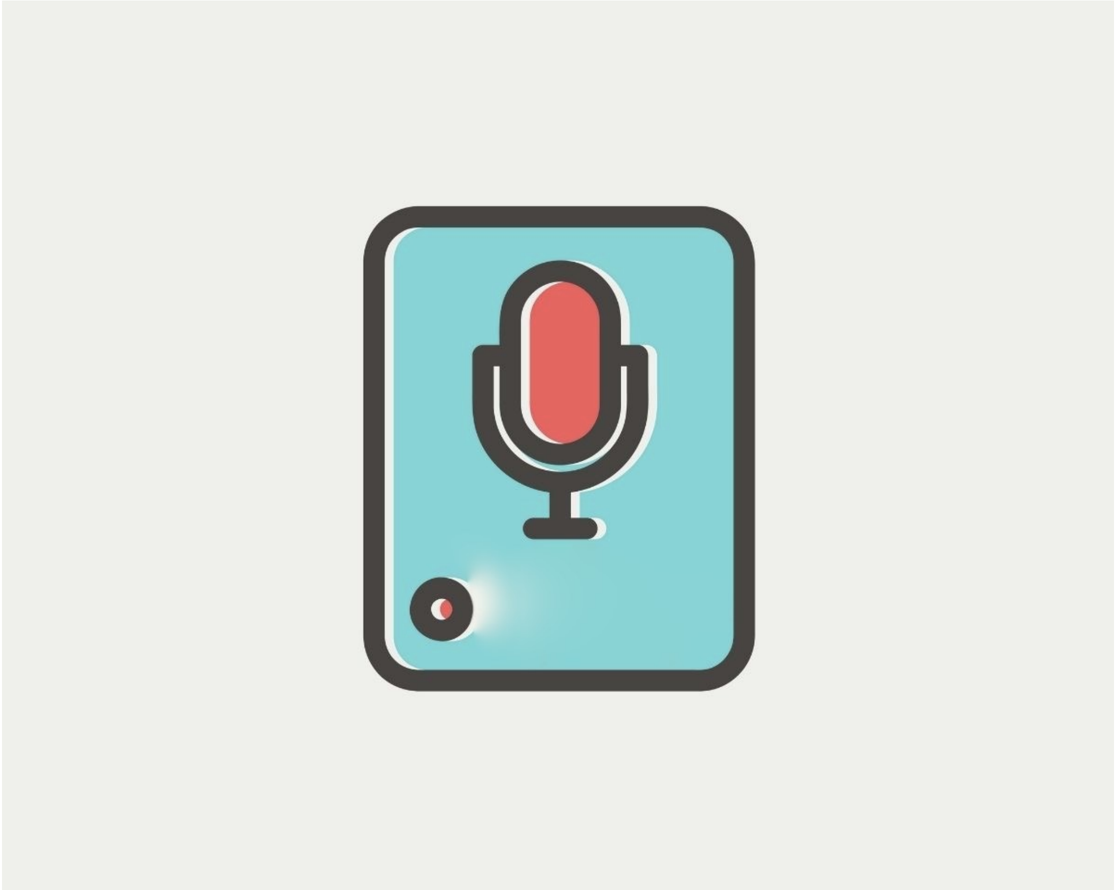

2

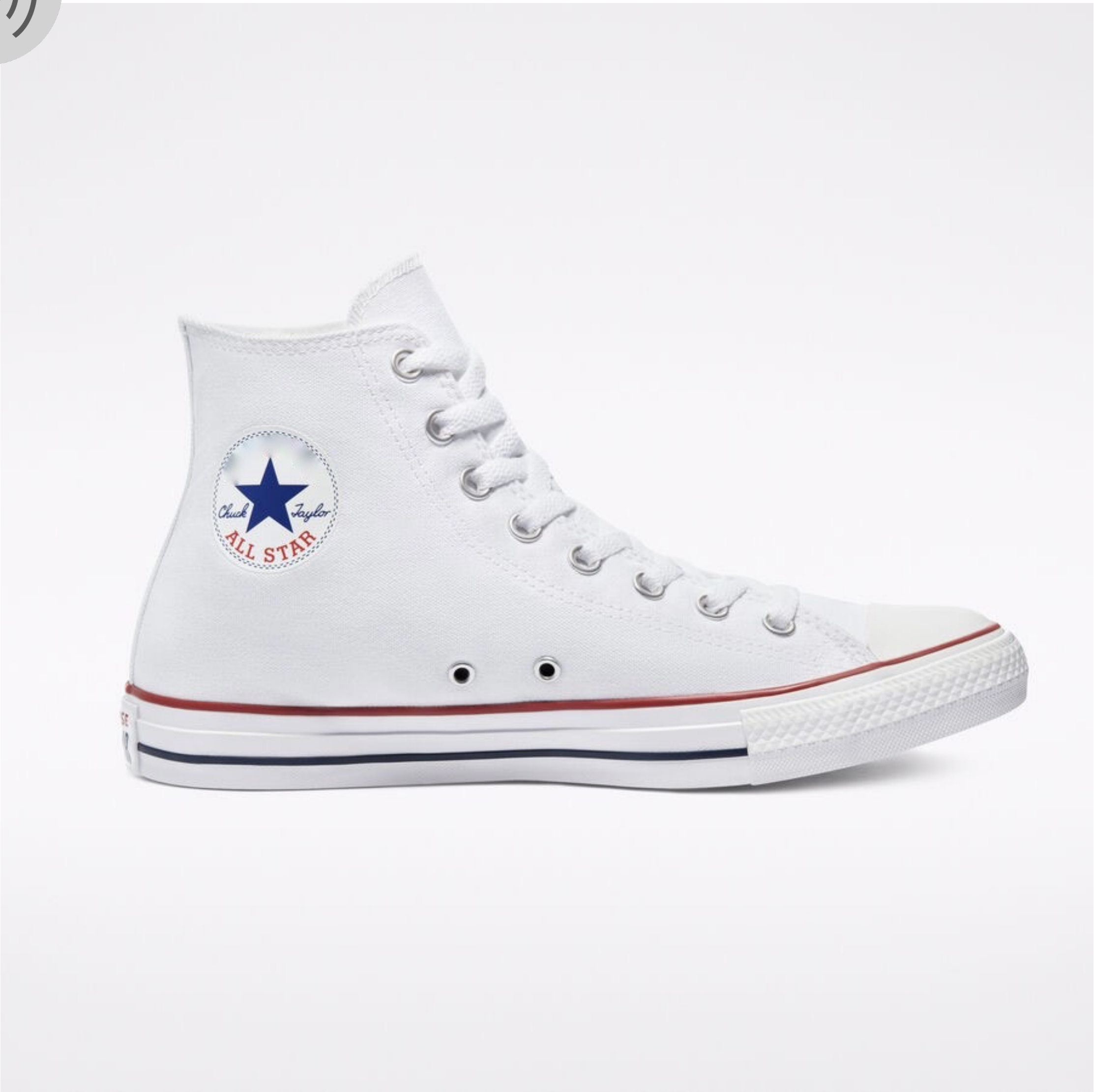

1

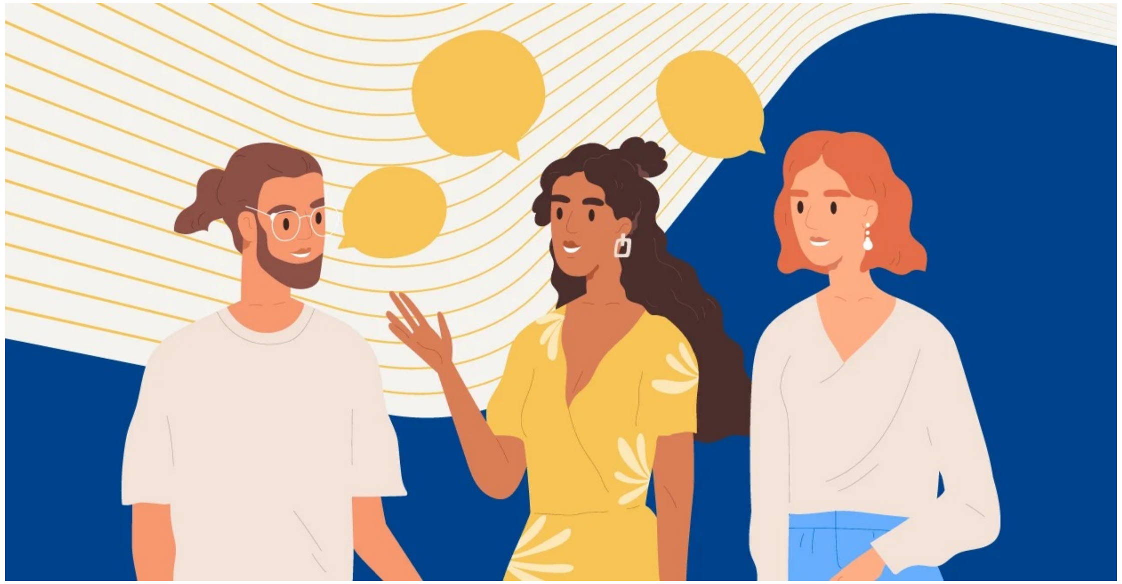

2

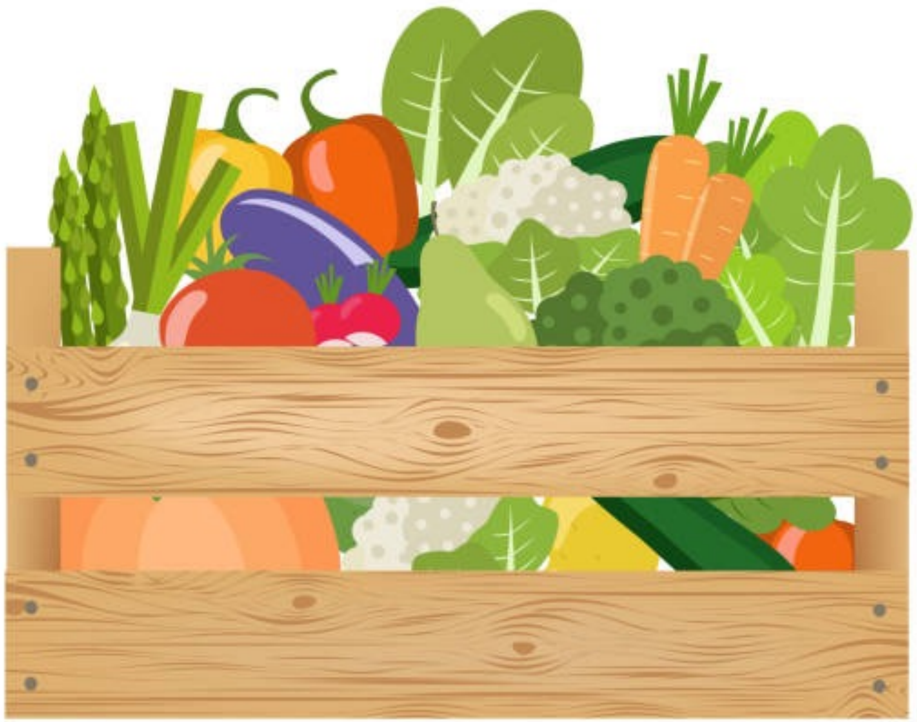

1

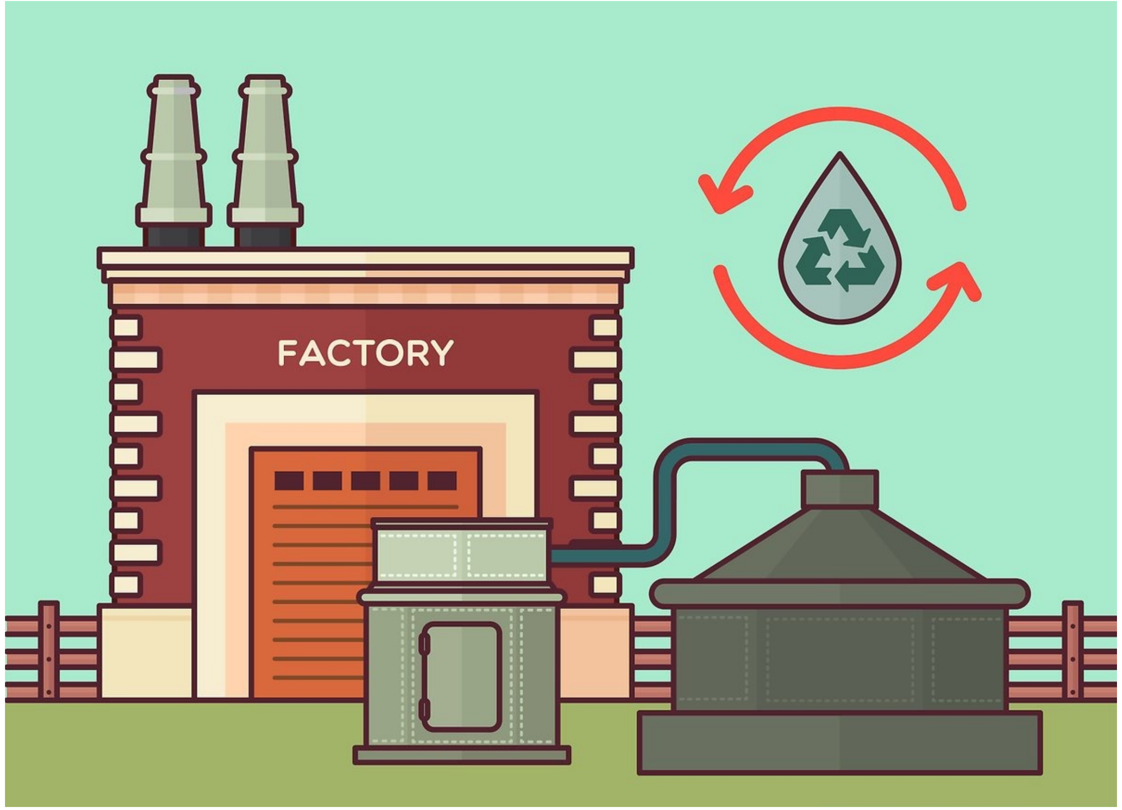

2

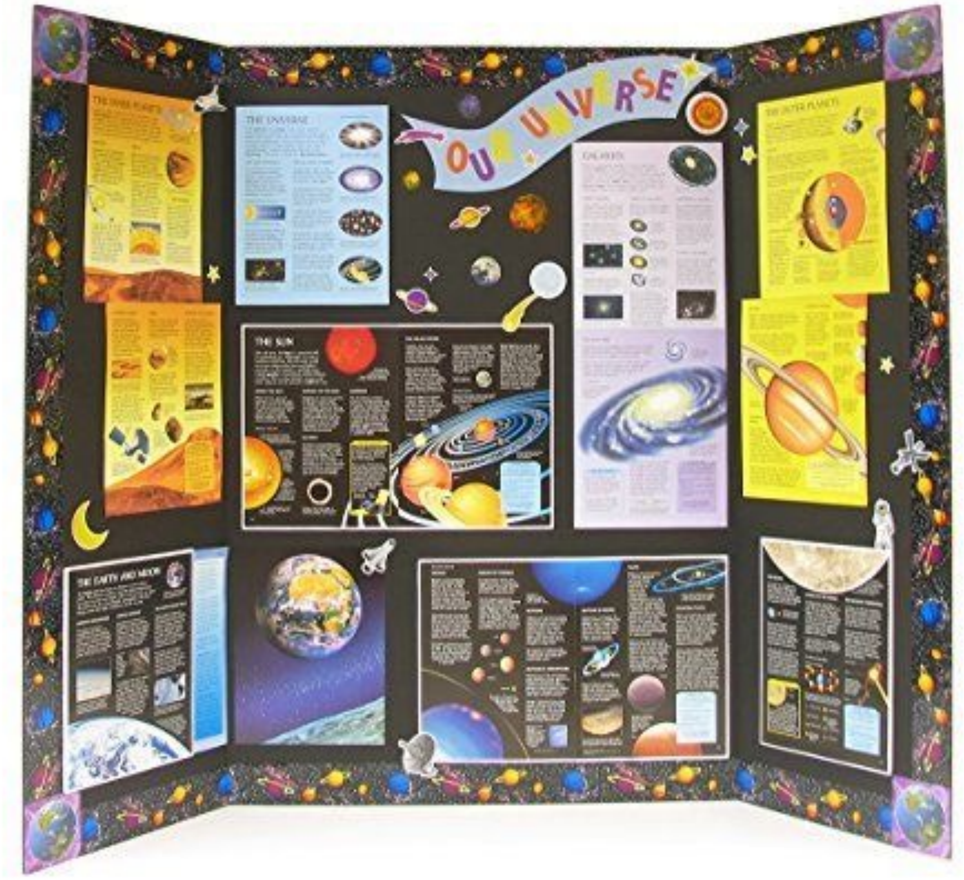

1

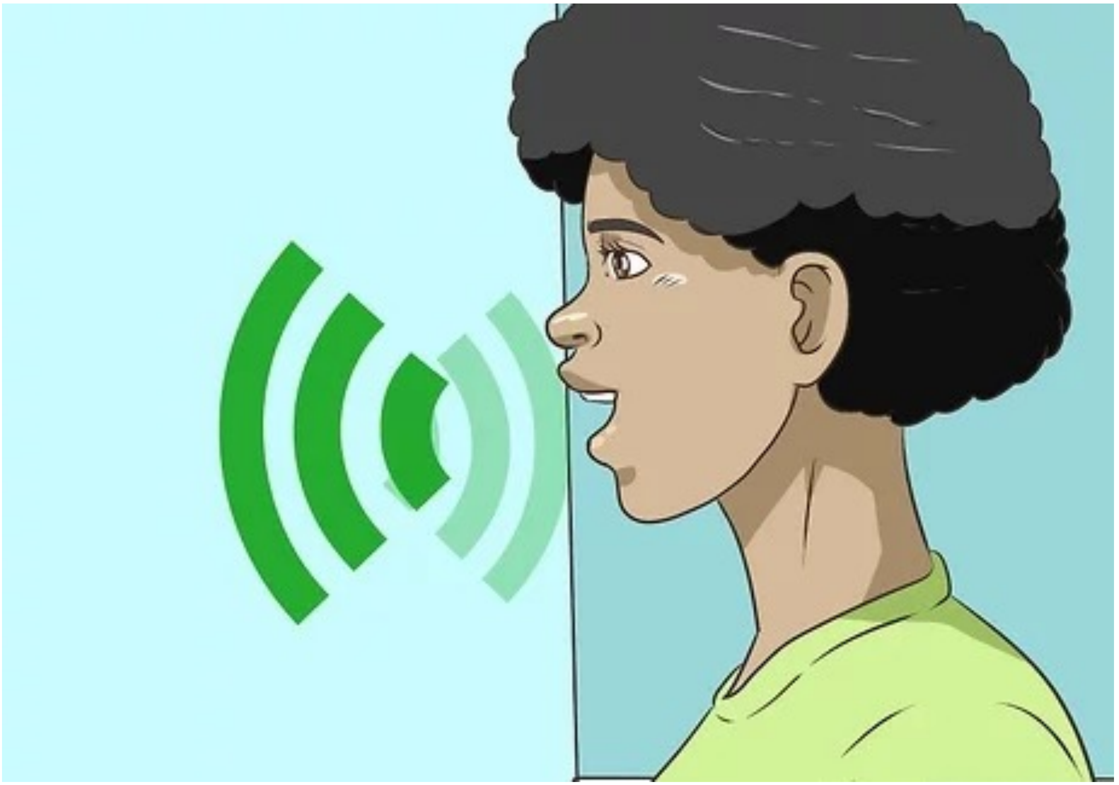

2

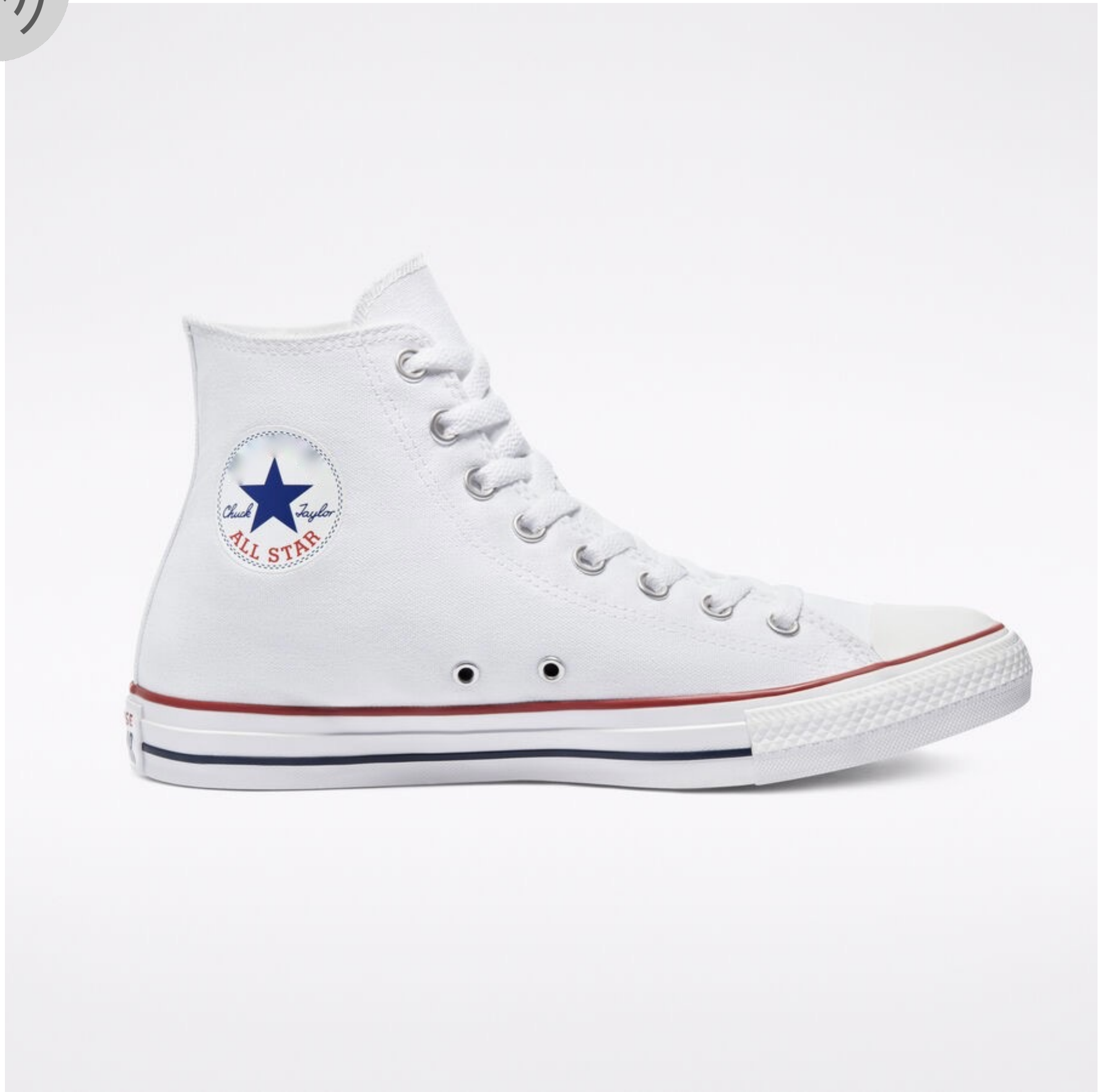

1

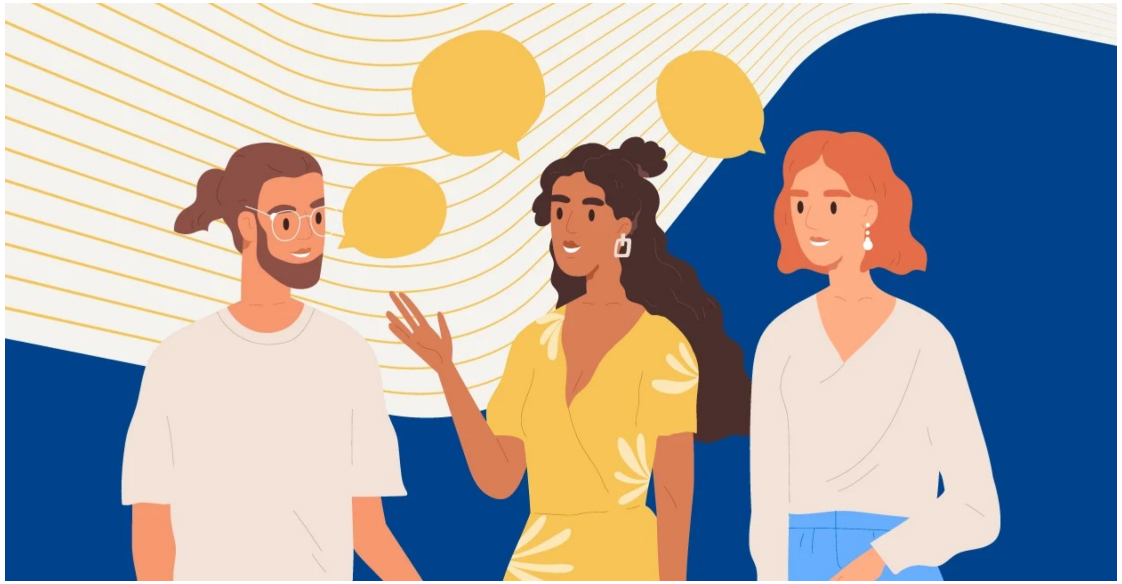

2
