# Supplementary material for: Understanding development of Mainstream US English lexical stress using semi-naturalistic stimuli
Source: PLoS One. 2026 Apr 29;21(4):e0345745. doi: 10.1371/journal.pone.0345745 (PMC13128110; doi:10.1371/journal.pone.0345745)
Supplement: S3 Files — (ZIP) [file pone.0345745.s001.zip › S3_NLSTaskFiles/NLS-ReceptiveTaskRecordForm.pdf]

Communication, Acquisition, and Translational Studies Laboratory

NLS Expressive Task Record Form

Participant ID: _____________

Scoring: Before scoring, indicate if there are distinct dialectal targets for 1st or 2nd syllable stress for each item. Then, score participant response when selecting the 1st or 2nd syllable-stressed word. After you note the response, you can calculate accuracy per item and then overall accuracy for the task.

**Slide #**	**Target** (1st or 2nd syllable)	**Response** (1st or 2nd syllable)	**Accuracy** (= 1 if Target matches Response; =0 if the do not match)
16	PREsent - 1		
17	obJECT - 2		
18	REcord - 1		
19	proJECT - 2		
20	PROduce - 1		
21	preSENT - 2		
22	OBject - 1		
23	reCORD - 2		
24	CONverse - 1		
25	proDUCE - 2		
26	PROject - 1		
27	converse - 2		
**Total Correct**			= _____ / 12 *100 = ______ % accurate

Please reach out to Jill Thorson, University of New Hampshire, with any questions: jill.thorson@unh.edu
